# TranscriptAchilles: a genome-wide platform to predict isoform biomarkers of gene essentiality in cancer

**DOI:** 10.1093/gigascience/giz021

**Published:** 2019-04-03

**Authors:** Fernando Carazo, Lucía Campuzano, Xabier Cendoya, Francisco J Planes, Angel Rubio

**Affiliations:** 1 Tecnun (University of Navarra), Paseo Manuel Lardizábal 15, 20018 San Sebastián, Spain. Department of Biomedical Engineering and Sciences; 2 University of Luxembourg, 2, avenue de l'Université, 4365 Esch-sur-Alzette, Luxembourg

**Keywords:** gene essentiality, RNAi screen, RNA-sequencing, transcriptomics, alternative splicing, cancer, biomarker, web tool, precision medicine

## Abstract

**Background:**

Aberrant alternative splicing plays a key role in cancer development. In recent years, alternative splicing has been used as a prognosis biomarker, a therapy response biomarker, and even as a therapeutic target. Next-generation RNA sequencing has an unprecedented potential to measure the transcriptome. However, due to the complexity of dealing with isoforms, the scientific community has not sufficiently exploited this valuable resource in precision medicine.

**Findings:**

We present TranscriptAchilles, the first large-scale tool to predict transcript biomarkers associated with gene essentiality in cancer. This application integrates 412 loss-of-function RNA interference screens of >17,000 genes, together with their corresponding whole-transcriptome expression profiling. Using this tool, we have studied which are the cancer subtypes for which alternative splicing plays a significant role to state gene essentiality. In addition, we include a case study of renal cell carcinoma that shows the biological soundness of the results. The databases, the source code, and a guide to build the platform within a Docker container are available at GitLab. The application is also available online.

**Conclusions:**

TranscriptAchilles provides a user-friendly web interface to identify transcript or gene biomarkers of gene essentiality, which could be used as a starting point for a drug development project. This approach opens a wide range of translational applications in cancer.

## Introduction

Alternative splicing (AS) is the mechanism by which a single pre–messenger RNA (mRNA) molecule can lead to different mature mRNA molecules, called isoforms or transcripts. Through this process, a gene is capable of encoding different proteins [1]. The number of discovered isoforms increases as the study of an organism improves. In humans, ∼95% of multi-exonic genes present AS events in diverse conditions [2].

AS occurs as a normal process in cells. However, there are some genetic aberrations—such as mutations or expression changes of splicing factor genes [3]—that affect AS and may result in the expression of less standard isoforms that produce an anomalous gain or loss of protein function. AS has been shown to play a pivotal role in the development of several diseases, including cancer. Specifically, all the hallmarks of cancer (e.g., angiogenesis, cell immortality, avoiding immune system response) are found to have a counterpart in aberrant splicing of key genes [4–6]. In recent years, AS is being used as a prognosis biomarker, a therapy response biomarker, and even as a therapeutic target in cancer [7, 8].

Several studies have analyzed the influence of AS in different contexts, as reviewed in Carazo et al [9]. These studies are usually based on the study of the relative or absolute concentration of transcripts looking for isoform changes across different conditions [10, 11]. Because the best biomarkers for a certain condition can be either genes or isoforms, it would be desirable to develop a methodology that integrated transcript and gene expression to provide the best biomarkers regardless of whether they are a gene or a transcript.

In the context of cancer, identifying genes that are essential to cellular viability is a potential source of drug targets. Analyzing mutant phenotypes and gene repression is especially relevant to this aim. One selective and efficient way to post-transcriptionally suppress gene expression is RNA interference. Project Achilles [12] performed genome-wide RNA interference screening in different cohorts of cancer cell lines, aiming to establish cancer dependencies and essential genes. Analyzing the biological output data of these experiments has been a challenge, mainly as a result of the off-target hybridizations of the RNA interference (RNAi) seed sequences. The DEMETER score [13] is a statistical summarization of essentiality scores that quantizes the competitive proliferation of the cell lines and minimizes the effect of off-target hybridizations by using a statistical model. DEMETER outperforms other summarizations such as the ATARiS score [14] or Bayes factors [15]. Recently, the authors of DEMETER have published an improved estimation of the essentiality score [16].

Different studies have successfully used Project Achilles data in combination with other omics data to define novel personalized treatments, mainly based on mutations and copy number variations [13, 14, 17]. Moreover, several web tools allow the visualization of Project Achilles data, such as Depmap [18]. However, little work has been done to relate Project Achilles with AS.

Here, we present TranscriptAchilles [19], a computational genome-wide tool that exploits gene and isoform expression as biomarkers of gene essentiality in the context of cancer. It integrates loss-of-function RNAi screening with whole-transcriptome expression profiling of 412 cancer cell lines. Using this tool, we have studied which are the cancer subtypes for which AS plays a significant role to identify gene essentiality. In addition, we include a case study of renal cell carcinoma that shows the biological soundness of the results. This approach opens a wide range of translational applications in cancer.

## Findings

### TranscriptAchilles pipeline

We have developed a statistical pipeline to predict the best biomarkers (genes or transcripts) of gene essentiality. The model is based on *limma* [20] to state the probability of a gene/transcript to be differentially expressed in cell lines that are sensitive to gene silencing.

TranscriptAchilles uses the essentiality score of DEMETER. The DEMETER score quantizes the competitive proliferation of the cell lines and minimizes the effect of off-target hybridizations by using a statistical model. The more negative the DEMETER score is, the more essential the gene is for a cell line. Authors of the DEMETER score established a cutoff of –2 as a threshold of essentiality. Genes with a DEMETER score lower than this threshold can be considered essentials for a cell line.

Although DEMETER's authors performed some validations of their essentiality score, we did 2 simple tests to confirm its reliability. First, we checked that genes are expressed when they are essentials (DEMETER score <–2). We found that genes are expressed >1 transcript per million (TPM) in 85% of the cases when they are essential, versus 70% when they are nonessential (Wilcoxon test *P* < 2.2e–16).

Second, we checked the essentiality scores of some well-known driver oncogenes related to their mutational state. Figs. S8–S13 show the DEMETER score for different cell lines grouped by their mutation status in *KRAS*, *BRAF*, *NRAS*, and *PIK2CA*. We found that mutated cell lines are sensitive to the knock-down (KD) of the activated oncogenes; this effect is known as “oncogene addiction” [21]. We also checked that the mutation status of *TP53* affects the essentiality of *MDM2* and *MDM4* as expected, because *MDM4* and *MDM2* regulate the activity and the stability of *TP53*, respectively [22]. We confirmed, in all the cases, that the relationships between DEMETER and mutation status are in accordance with the bibliography.

**Figure 1:**
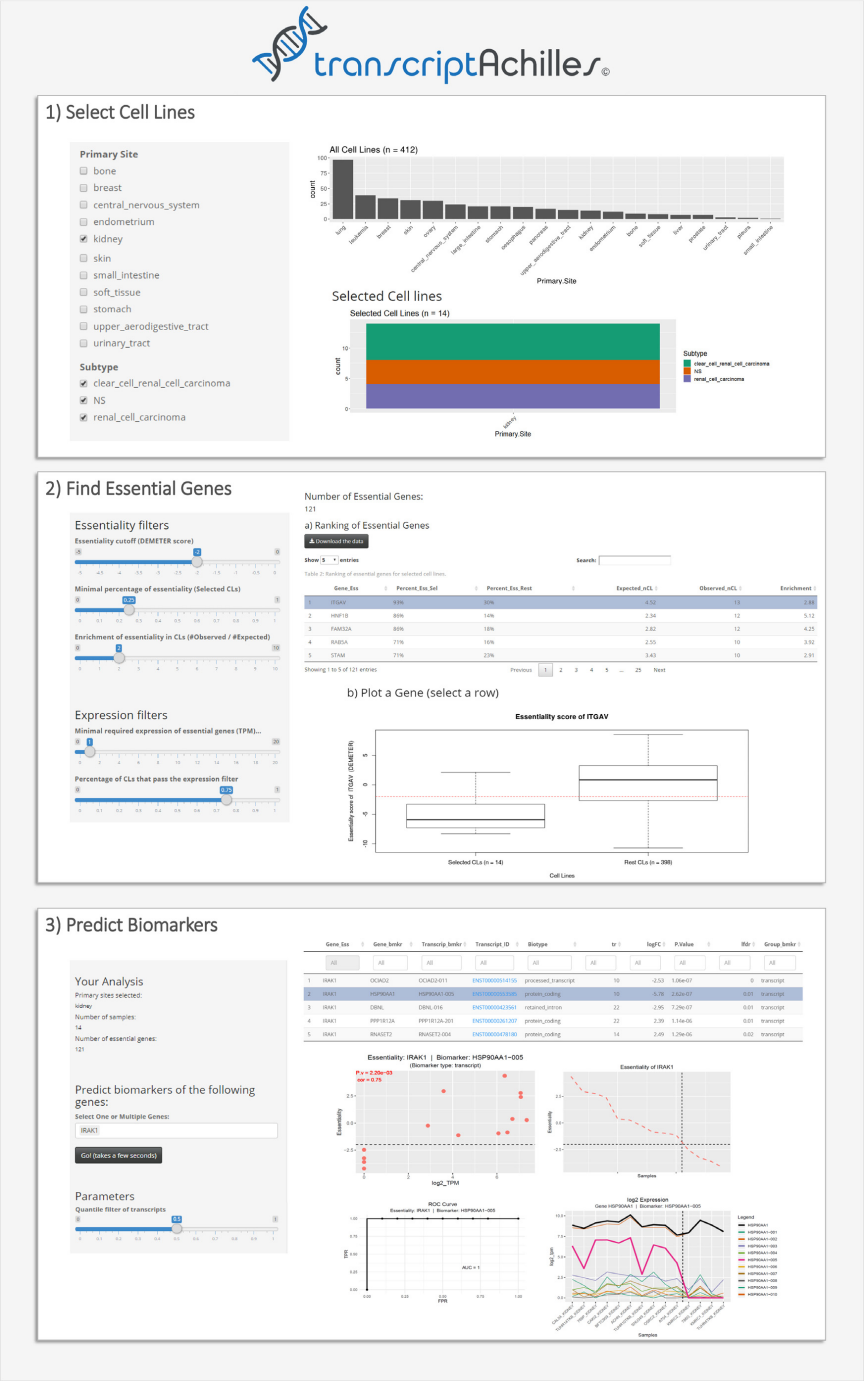
Screenshots of the 3 main tabs of TranscriptAchilles. (1) Selection of cell lines. Both primary site and subtypes can be selected. Two histograms summarize the number of all (up) and selected (down) cell lines. (2) Find Essential Genes. This functionality finds genes whose inhibition reduces the proliferation of the selected cohort. The returned genes are essential, specific, and expressed in the selected cell lines. All the parameters can be tuned with the sliders. A ranking of essential genes and a box plot of essentiality (DEMETER score) for the selected cohort (left) and the rest of cell lines (right) are shown. The red dotted line marks the default essentiality score of –2 dividing the samples into resistant (up) and sensitive (down) to the KD. In this case, the essential gene selected in the ranking table is *ITGAV*. (3) Predict biomarkers (both transcripts and genes) for the essential genes selected by the user. This analysis can be run for every essential gene in the other tab. The ranking of biomarkers has the following columns: Gene_Ess: essential gene; Gene_bmkr and Transcript_bmkr: gene/transcript expression biomarker; tr: number of transcripts of the corresponding gene; logFC: log2 Fold change of expression; Lfdr: local false discovery rate; Group_bmkr: indicates whether the best biomarker is a gene or a transcript. See legend of Fig. 2 for a more detailed explanation of the plots.

In addition, we have developed an open and intuitive visual platform to allow researchers to perform their own analysis following simple steps. The platform is presented in 3 main panels, as shown in Fig. 1.

The main panels of the platform are as follows:

#### Select cell lines

The user can select the cohort of cell lines to be analyzed. Several primary sites and subtypes can be selected at the same time. The application is preloaded with all the necessary data, so that the user does not need to upload any file.

#### Find essential genes

Based on the Achilles Project data, TranscriptAchilles identifies essential genes for the selected cell lines. These genes are required to meet several criteria: (i) they must be essential for a minimum percentage of samples in the selected subtype, (ii) they must be specific for the subtype under study, and (iii) they must be expressed. To achieve these 3 requirements, the user can tune several thresholds. The first one is the percentage of cell lines that are sensitive to the gene KD of interest. The second one is an odds ratio, which can be illustrated with an example: if the enrichment is set to 2, the percentage of cell lines sensitive to the gene KD must be 2 times larger for the cell lines under study than for the rest of the cell lines in the DEMETER data set. Finally, a threshold on expression can be set to ensure that the genes are expressed when they are essential.

#### Predict biomarkers for a target gene

In this section, the user can select ≥1 genes from the previous step and predict putative biomarkers of their essentiality. The statistical model estimates the local false discovery rate (local FDR) for both genes and transcripts and decides whether genes or transcripts are the best markers for each case (see Methods section). The user can also find biomarkers for all the essential genes identified by running the panel “Find Essential Genes" in the tab "Predict Genome-Wide Biomarkers”.

### Implementation and availability

TranscriptAchilles (SciCrunch.org RRID:SCR_016849) has been fully developed using R [23] and Shiny [24]. The databases and source code are available at GitLab [25]. Once the git repository is cloned, TranscriptAchilles can be run locally following the instructions included in the repository. The application can also be run locally within Docker to avoid installation problems and to facilitate reproducibility. TranscriptAchilles is hosted using the Amazon Web Services cloud environment service on the server [26]. The security of the app is managed by using the ShinyProxy framework [27].

### Splice-based overview of tumor subtypes

We conducted several comparisons throughout 20 tumor subtypes to quantify the potential of genes and transcripts to be used as biomarkers of essentiality. We ran our pipeline for every tumor subtype with ≥7 samples (20 tumor subtypes). For each of them, we identified a set of genes that are essential in the selected cohort of cell lines by running the Find Essential Genes tab (essential: DEMETER score <–2; specific: enrichment of essentiality ≥ 1; expressed: TPM > 1).

Using our statistical pipeline, we predicted which genes or transcripts are potential biomarkers of gene essentiality. A condition affected by splicing is more likely to have more transcript biomarkers than one with no splicing changes. To estimate this characteristic, we compared the proportion of genes/transcripts relative to the total number of predicted biomarkers (i.e., if a certain essential gene has 10 potential biomarkers, 9 of which are “transcripts”, we would say that 90% of its biomarkers are transcripts) (Fig. 3).

**Figure 2:**
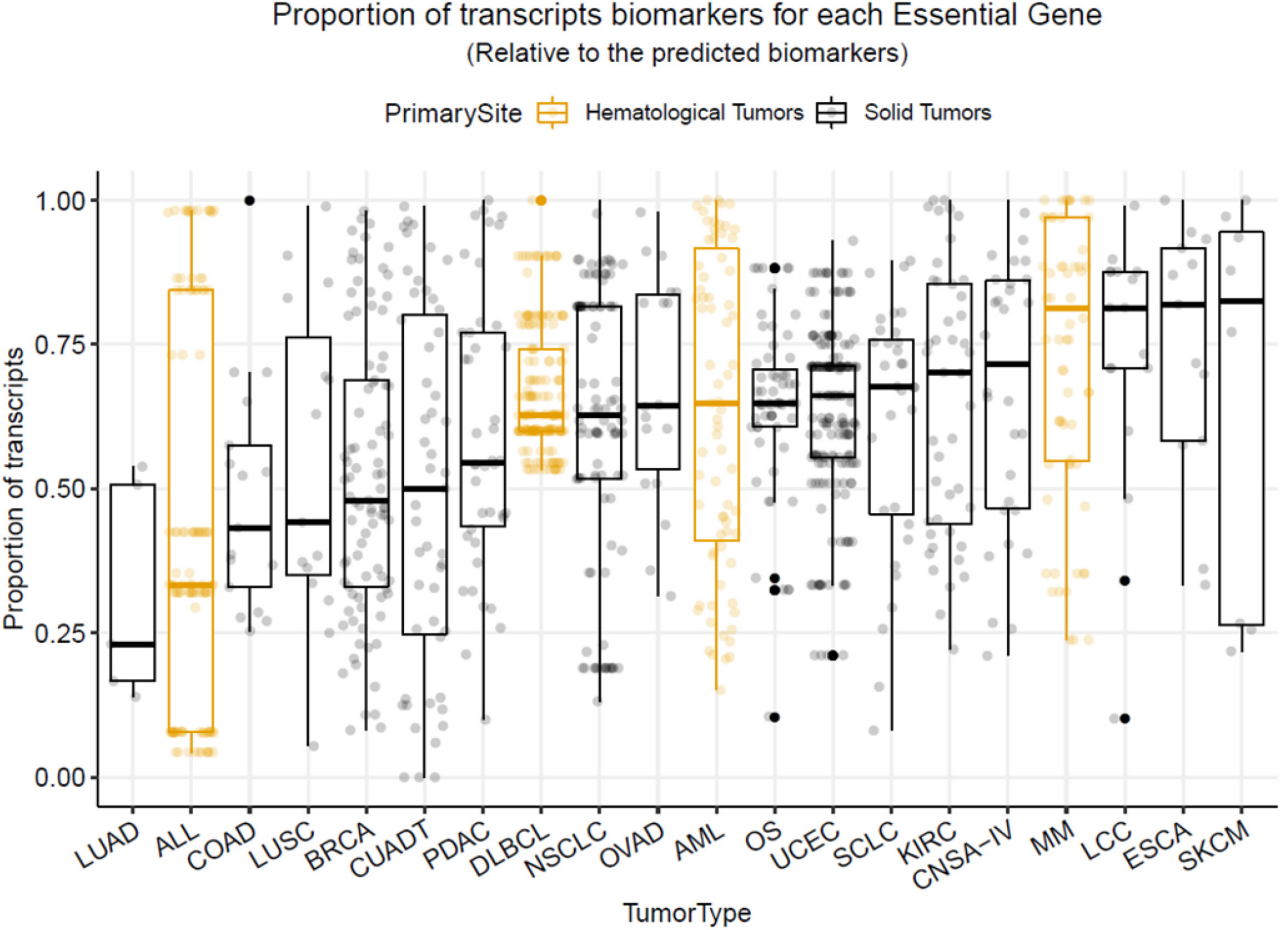
Percentage of transcripts predicted to be biomarkers of essential genes in 20 tumor types. Each essential gene has different biomarkers: some of them are genes and others are transcripts. Each point of the box plots represents the proportion of transcript biomarkers for an essential gene for a given tumor type. ALL: acute lymphoblastic leukemia; AML: acute myeloid leukemia; BRCA: breast ductal carcinoma; CNSA-IV: central nervous system astrocytoma grade IV; COAD: colon adenocarcinoma; CUADT: upper aerodigestive tract squamous cell carcinoma; DLBCL: diffuse large B-cell lymphoma; ESCA: esophagus squamous cell carcinoma; KIRC: kidney renal clear cell carcinoma; LCC: lung large cell carcinoma; LUAD: lung adenocarcinoma; LUSC: lung squamous cell carcinoma; MM: multiple myeloma; NSCLC: non–small cell lung carcinoma; OS: osteosarcoma; OVAD: ovary adenocarcinoma; PDAC: pancreas ductal carcinoma; SCLC: small cell lung carcinoma; SKCM: skin carcinoma; UCEC: endometrium adenocarcinoma.

Differences found within the tumor types were strongly significant (Kruskal-Wallis *P* = 3.18e–16). Skin carcinoma, esophagus squamous carcinoma, lung large cell lung carcinoma, and multiple myeloma are the most splicing-influenced cancer subtypes. On the other hand, isoforms have less predictive power in lung adenocarcinoma, acute lymphoblastic leukemia, and colon adenocarcinoma. These findings are in accordance with a recent large-scale study of 4,542 patients from The Cancer Genome Atlas, which measured driver and functional isoform switches in 11 cancer types [11]. Within the tumor types shared with our study, kidney carcinoma and colon adenocarcinoma were the cancers with the most and the fewest driver isoform switches, respectively. Lung squamous carcinoma was more affected by splicing switches than lung adenocarcinoma. In addition, we found that within hematological tumors, acute lymphoblastic leukemia had the lowest proportion of transcript biomarkers. Diffuse B-cell lymphoma, acute myeloid leukemia, and multiple myeloma had more than half of their essential genes better predicted by transcripts.

Considering the whole transcriptome as the source for biomarkers, we studied the recurrence of each transcript biotype of the predicted biomarkers in comparison to the general biotypes (Fig. 4). Ensembl [28] catalogs transcripts into 4 main biotypes: protein coding, pseudogene, long noncoding, and short noncoding. These 4 main groups contain 35 subcategories in total. More than 90% of the transcriptome of the 412 cell lines taken together falls into 7 biotypes (out of 35), namely, protein coding, nonsense-mediated decay, long intergenic noncoding RNA, microRNA, antisense, processed transcript, and retained intron. Protein-coding transcripts is the most represented category (∼40% of transcripts).

**Figure 3:**
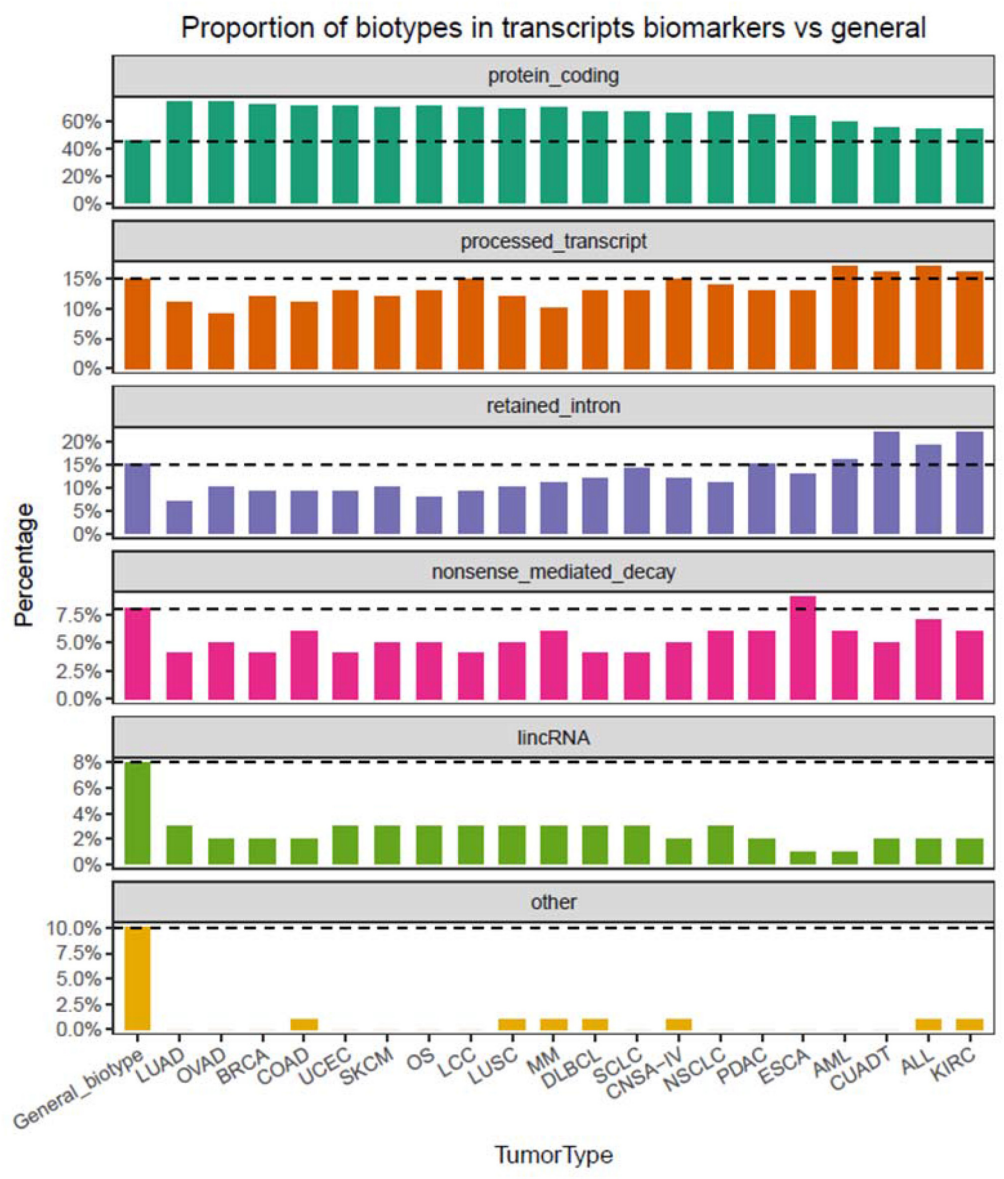
Proportion of transcript biotypes of biomarkers in 20 tumor types vs in general. Acronyms are included in Fig. 3 caption. General_biotype shows the proportion of each specific biotype in the reference transcriptome (Gencode 24). Protein-coding transcripts are overrepresented as biomarkers for all tumor types. lincRNA: long intergenic noncoding RNA.

**Figure 4:**
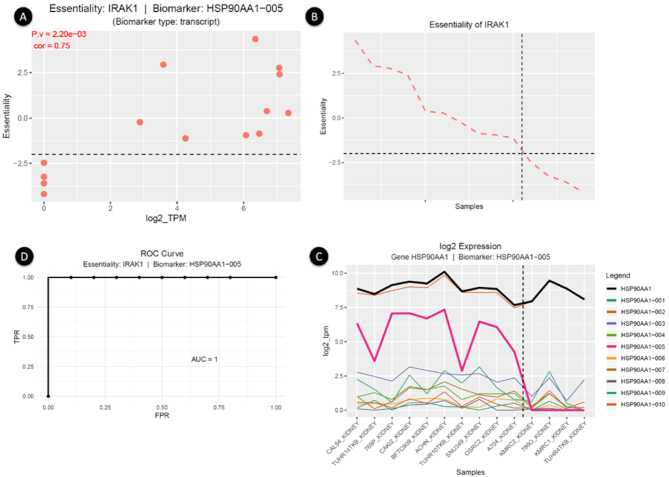
Output of TranscriptAchilles in renal carcinoma cell lines (n = 14). HSP90AA1-005 is a transcript biomarker of essentiality of *IRAK1*. (A) Scatterplot of *IRAK1* essentiality and HSP90AA1-005 log2-expression. Each dot represents a single cell line. The dotted black line marks the –2 essentiality threshold. (B) Essentiality of *IRAK1*. Samples are sorted by their essentiality (more negative implies *IRAK1* is more essential). Samples in panels B and C are sorted in the same order. The x-axes are shared by both panels. The black line marks the default essentiality score of –2 dividing the samples into resistant and sensitive to *IRAK1* KD. (C) log2-expression of gene HSP90AA1 (black line) and its transcripts. The dotted black line divides cell lines into resistant (left side) and sensitive (right side). The best biomarker (HSP90AA1-005) is shown in pink. In this case, transcript expression provides better essentiality markers than gene expression. (D) Receiver operating characteristic (ROC) curve of the selected biomarker. Here the AUC is 1, but this is not generally the case.

We examined whether the biomarker's biotypes mimic the general distribution of biotypes in the transcriptome (Fig. 4). Remarkably, 5 biotypes accounted for the vast majority of the biomarkers. Protein-coding transcripts were the most abundant category across the 20 cell line subtypes, and tended to be overrepresented when compared with the global proportion. MicroRNA and other small RNAs are underrepresented in the table. This result makes sense because short RNAs are usually depleted before sequencing and thus, microRNA concentration cannot be properly measured. Intron retention is, with nonsense-mediated decay, the third most represented transcript biotype. The widespread abundance of intron retention in tumor transcriptome is well documented [29], but, to our knowledge, it has not been proposed as a possible source of biomarkers [30] or even neoantigens [31]. In fact, our results suggest that coding isoforms are better biomarkers. The roles of intron retention in cancer have yet to be elucidated. The primary fate of this class of AS is degradation through the nonsense-mediated mRNA decay (NMD) mechanism. NMD results in reduced parent gene expression. However, it has been shown that certain intron retentions are capable of avoiding NMD and have been postulated to regulate the function of the parent gene in a dominant-negative manner [32].

### Case study

To further illustrate the potential of this platform in precision medicine, we show a case study using renal carcinoma cell lines (n = 14). We first conducted the gene essentiality analysis of these cell lines. We selected genes (i) essential in ≥25% of renal cancer cell lines, setting the threshold for the DEMETER score as –2; (ii) with a specificity odds ratio of ≥2; and (iii) with a minimum expression of 1 TPM in ≥75% of cell lines when the genes are essential. Applying these parameters, 121 genes were found to be essential for renal carcinoma. Some of these genes belong to pathways known to be dysregulated in renal cancer (e.g., *ITGAV*, *TIAM1*, and *PIK3CB*) [33]. Interestingly, 73 of 121 genes (*P* = 1.1e–3, Fisher exact test) have previously been identified as potential cancer drivers in other tumor types in mice according to the Candidate Cancer Gene Database [34].

Among these genes, *PAX8* and *HNF1B* play a key role in renal carcinoma [35, 36]. PAX proteins are transcription factors that regulate cell proliferation and migration of embryonic precursor cells [37]. The depletion of *PAX2* by RNAi induces apoptosis in kidney carcinoma [38]. In addition, *PAX2* and *PAX8* double-mutant cells do not exhibit mesenchymal–epithelial transition and in turn lack mesonephric tubules [39]. On the other hand, *HNF1B* is a transcription factor that acts as a tumor suppressor in renal carcinoma through control of *PKHD1* expression [40].

Biomarkers for essential genes were obtained by running the “Predict Biomarkers for a Target Gene” panel. In this case, we focused on the interleukin-1 receptor-associated kinase (*IRAK*), which is implicated in cancer initiation and progression [41]. TranscriptAchilles revealed that all the proposed biomarkers for *IRAK1* (*P* < 1e–4, |log2 fold change| > 2, and local FDR < 0.1) were transcripts, which stresses the importance of splicing as a source of biomarkers.

The HSP90AA1-005 transcript is one of the best markers of *IRAK1* essentiality (Fig. 2). The *HSP90* gene plays a role in the regulation of *IRAK1* [42]. Interestingly, while gene expression is not capable of distinguishing between sensitive and resistant groups of cell lines (*P* = 0.37; local FDR = 0.7; AUC = 0.63), the predicted transcript HSP90AA1-005 is a good biomarker of *IRAK1*’s essentiality (*P* = 2.62e–07; local FDR = 0.01; AUC = 1).

TranscriptAchilles can also predict genome-wide biomarkers for all essential genes and rank them according to their significance. We found companion biomarkers for 101 essential genes (out of 121). In 60% of cases, the best markers were transcripts rather than genes.

Fig. 2 and Figs. S6 and S7 show 3 essential gene and biomarker pairs (*IRAK1*/HSP90AA1-005, *PER3*/SEC31A-020, *IRAK1*/MAPK1*-*201). In these cases, transcripts are differentially expressed between sensitive and resistant cell lines, while the corresponding genes do not show this pattern. In addition, >95% of the proposed biomarkers for *IRAK1* and *PER3* were transcripts (*P* < 1e–4, |log2 fold change| > 2, and local FDR < 0.1).

The suggested essential gene-biomarker pairs are biologically sound. The interleukin-1 receptor-associated kinase (*IRAK*) plays a key role in the toll-like receptor (*TLR*) and interleukin-1 receptor (*IL1R*) signaling pathways, which are implicated in cancer initiation and progression [41]. Mitogen-activated protein kinase (*MAPK*) is involved in the regulation of normal cell proliferation, survival, and differentiation. Aberrant regulation of *MAPK* contributes to cancer through the well-studied *Ras*-*Raf*-*MEK*-*ERK* pathway [43]. The relationship between *MAPK* and *IRAK* is also documented. *IRAK* participates in the activation of p38 *MAPK* by associating with *Ras* [44].

## Methods

### Data sources and preprocessing

The Cancer Cell Line Encyclopedia (CCLE)(CCLE, RRID:SCR_013836) [45] provides public access to genomic data of nearly 900 cancer cell lines. The transcriptome profiles of these samples were calculated in a previous study [46] from raw RNA-sequencing data using Kallisto (kallisto, RRID:SCR_016582) [47]. This study uses the Gencode 24 transcriptome (GRCh 38) as reference annotation [48]. This version of the transcriptome contains 199,169 transcripts. Transcript expression was measured in TPM and filtered. In the filtering step, we excluded transcripts that had zero TPMs in every sample. Then, for the selected cohort of cell lines, we required the average expression of transcripts to be above a threshold, whose default value is 50% quantile of all the average expressions. After these filters were applied, the resulting number of transcripts was ∼90,000. This number depends on the selection of cell lines.

In the Achilles Project, 412 of these cell lines were interrogated for gene essentiality using short hairpin RNA (shRNA). We used the DEMETER score as a measure of essentiality. DEMETER quantizes the competitive proliferation of the cell lines and minimizes the effect of off-target hybridizations by using a statistical model. The more negative the DEMETER score is, the more essential the gene is for a cell line. Authors of the DEMETER score established a cutoff of –2 as a threshold of essentiality. Genes with a DEMETER score lower than this threshold can be considered essentials for a cell line. Missing elements of DEMETER were imputed using the nearest neighbor averaging algorithm (KNN) [49].

**Figure 5:**
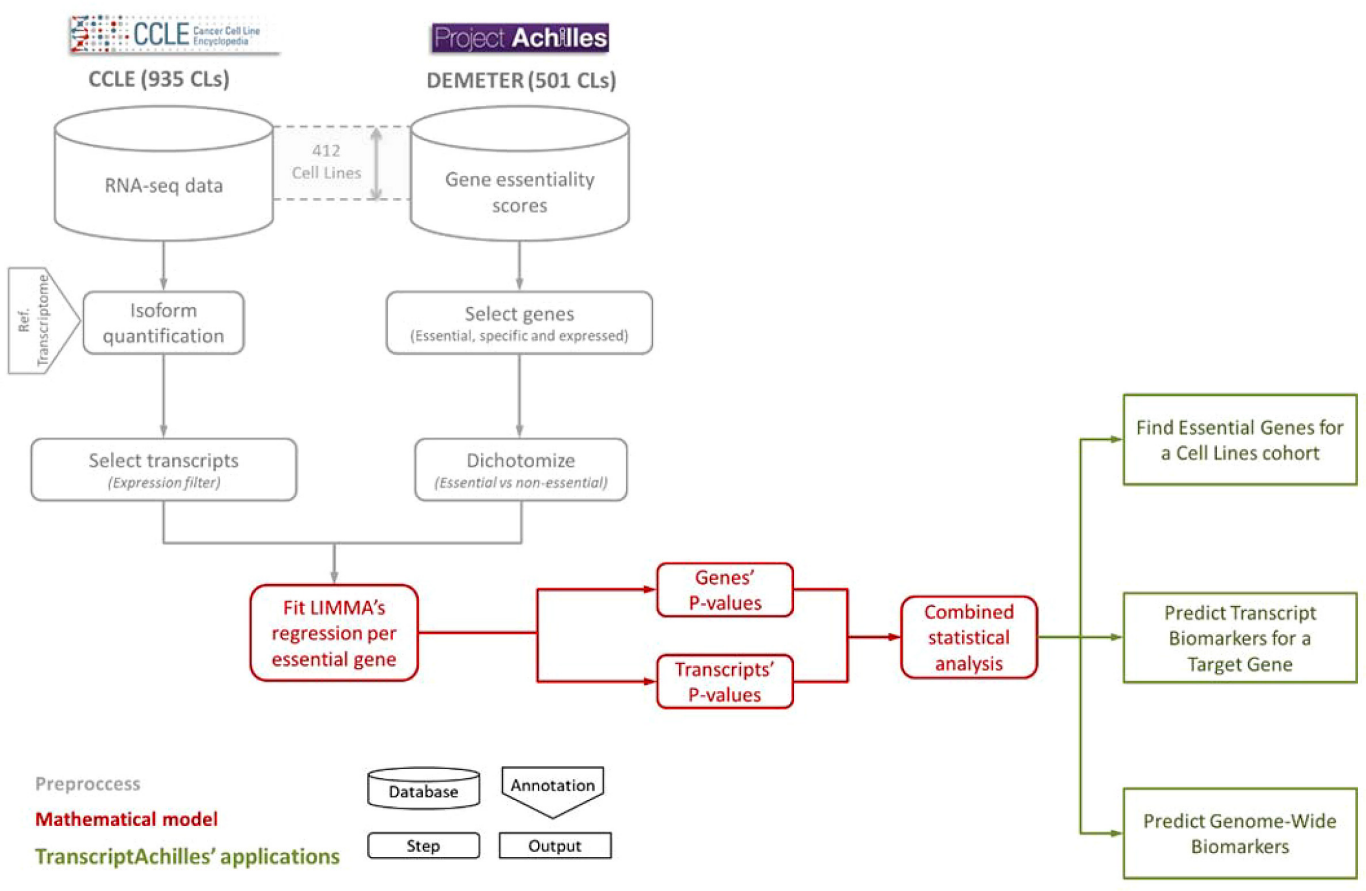
TranscriptAchilles’ workflow. Database icons represent CCLE and Project Achilles data. A total of 412 samples were matched between them. Step boxes represent algorithmic analysis, for both preprocessing (grey) and mathematical modeling (red). Green boxes represent applications of TranscriptAchilles. CL: cell line.

Combining gene and isoform expression and DEMETER, we developed a statistical pipeline to find essential genes and predict the best markers of essentiality (Fig. 5).

### Statistical model

Let *e* denote the number of RNAi target genes and *n* denote the number of screened samples. Let **D** be an $e \times n$ matrix of essentiality with each element ${d_{ij}}$ representing the DEMETER score for the RNAi target *i* in sample *j*. Let D* be an $n \times e$ dichotomized matrix whose each element $\ d_{ij}^{*}$ denotes whether sample *j* is resistant or sensitive to the RNAi target *i* as follows:

\begin{equation*}
{d_{ij}^{\rm{*}}} = \left\{ {\begin{array}{@{}*{1}{c}@{}} {1,\ {\rm{if}}\ {d_{ij}} < {\rm{thr}}\ \left( {{\rm{Sensitive}};{\rm{\ }}S} \right)\ }\\ {0,\ {\rm{otherwise}}\ \left( {{\rm{Resistant}};\ R} \right)} \end{array}} \right.\ ,
\end{equation*}

where thr is a threshold whose default value is −2 as proposed in DEMETER.

Let ***s*** be a subset of *N*cell lines that yields an essentiality vector ${\boldsymbol{d}}_{{{\boldsymbol{e}}_{\boldsymbol{s}}}}^{\boldsymbol{*}} = ( {{d_{{e_s}_1}},\ \ldots ,\ {d_{{e_s}_{N}}}} )\ $ for the *e*^th^ RNAi target. Let ${{\boldsymbol{y}}_{{{\boldsymbol{g}}_{\boldsymbol{s}}}}} = ( {{y_{{g_s}_1}},\ \ldots ,\ {y_{{g_s}_{N}}}} )\ $ be the expression vector of a putative gene biomarker and ${{\boldsymbol{y}}_{{{\boldsymbol{t}}_{\boldsymbol{s}}}}} = ( {{y_{{t_s}_1}},\ \ldots ,\ {y_{{t_s}_{N}}}} )\ $ be an expression vector of one of their corresponding transcripts. The null hypotheses are defined as

\begin{equation*}
H_0^g:{\rm{\ }}E\ \left( {{{\boldsymbol{y}}_{{{\boldsymbol{g}}_{\boldsymbol{s}}}}}{\rm{|}}{\boldsymbol{d}}_{{{\boldsymbol{e}}_{\boldsymbol{s}}}}^{\boldsymbol{*}} \in S} \right) = E\left( {{{\boldsymbol{y}}_{{{\boldsymbol{g}}_{\boldsymbol{s}}}}}{\rm{|}}{\boldsymbol{d}}_{{{\boldsymbol{e}}_{\boldsymbol{s}}}}^{\boldsymbol{*}} \in R} \right).
\end{equation*}

\begin{equation*}
H_0^t:{\rm{\ }}E\ \left( {{{\boldsymbol{y}}_{{{\boldsymbol{t}}_{\boldsymbol{s}}}}}{\rm{|}}{\boldsymbol{d}}_{{{\boldsymbol{e}}_{\boldsymbol{s}}}}^{\boldsymbol{*}} \in S} \right) = E\left( {{{\boldsymbol{y}}_{{{\boldsymbol{t}}_{\boldsymbol{s}}}}}{\rm{|}}{\boldsymbol{d}}_{{{\boldsymbol{e}}_{\boldsymbol{s}}}}^{\boldsymbol{*}} \in R} \right).
\end{equation*}

This null hypothesis is therefore “the mean expression of a biomarker is identical in resistant and in sensitive cell lines to a gene KD.” To test this hypothesis, we used a moderated t-test implemented in *limma* [20]. We applied this test for each RNAi target and all the expressed genes and transcripts to get the corresponding *P*-values. Dealing with these *P*-values implies solving 2 challenges: (i) integrating transcripts and genes to get the best biomarkers and (ii) correcting for multiple hypotheses.

**Figure 6:**
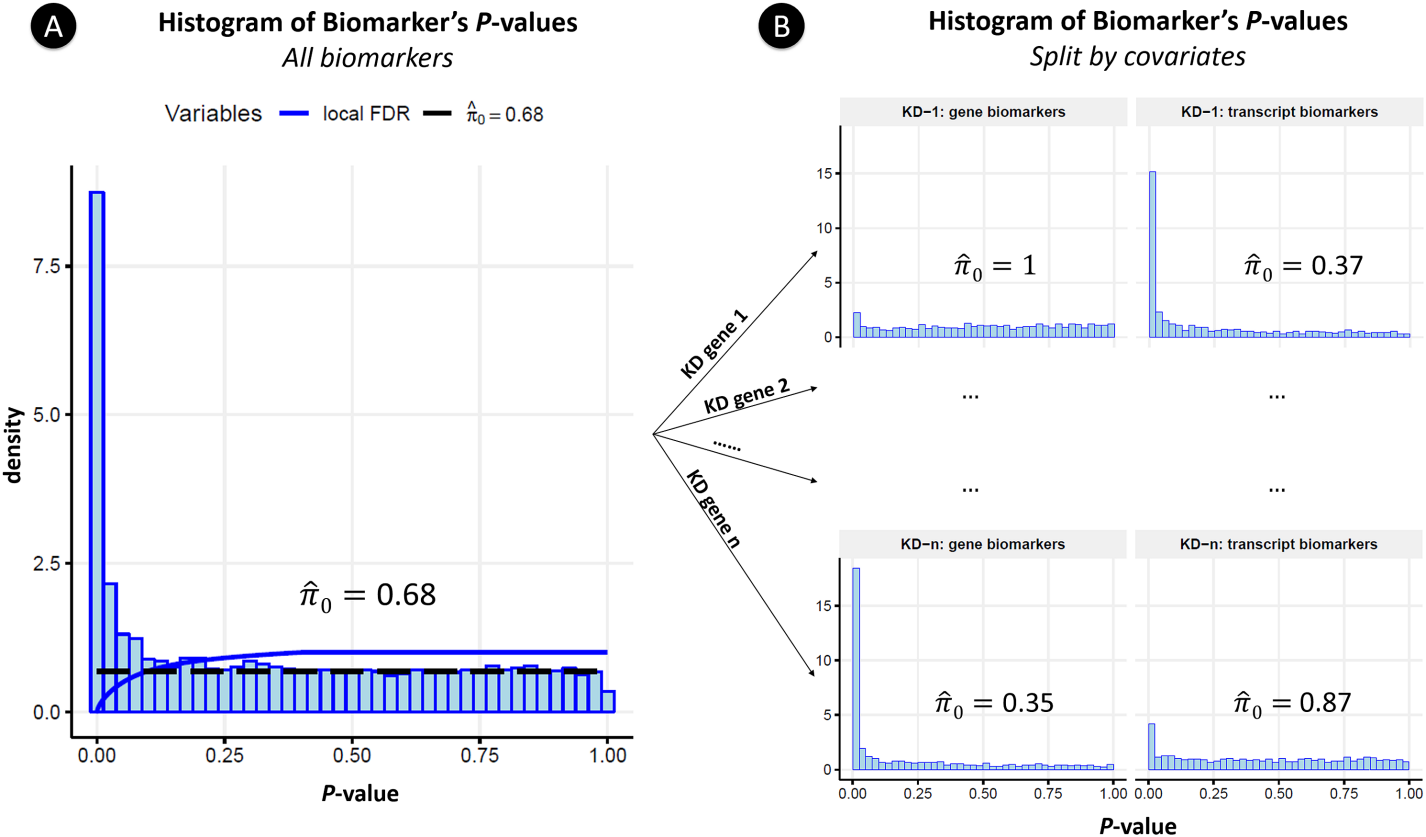
(A) Histogram of *P*-values of all tests (both genes and transcripts) taken together. The local FDR and the ${\hat{\pi }_0}$ (the proportion of true null hypotheses) values are shown. (B) Histogram of *P*-values after splitting by the covariates. The complete histogram in panel A gathers all the histograms in panel B. The covariates are, by rows: the KD genes; and, by columns: whether the biomarker is a gene or a transcript. In KD gene 1, transcripts are better biomarkers than genes (${\hat{\pi }_0} = 0.37\,{\rm{vs}}\,{\hat{\pi }_0} = 1$), and vice versa in KD gene *n* (${\hat{\pi }_0} = 0.35\,{\rm{vs}}\,{\hat{\pi }_0} = 1$).

To face these challenges, we followed a methodology similar to the independent hypothesis weighting procedure [50], which increases the power of a test by grouping the results using covariates. In our case, we divided the *P*-values corresponding to all the tests into 2*n* groups, where *n* is the number of KD genes (see Fig. 6). Each group includes the *P*-values of either the transcripts or genes interrogating each KD gene.

For each of these groups, we computed the local FDR [51]. The local FDR estimates, for each test, the probability that the null hypothesis is true, conditioned on the observed *P*-values. The formula of the local FDR is the following:

\begin{equation*}
P\ \left( {{H_0}{\rm{|}}z} \right) = local FDR\left( z \right) = \frac{{{\pi _0}{f_0}\left( z \right)}}{{f\left( z \right)}}\ ,
\end{equation*}

where *z* are the observed *P*-values; π_0_ is the proportion of true null hypotheses (estimated from the data); ${f_0}( z )$, the empirical null distribution—usually a uniform (0,1) distribution for well-designed tests- and $f( z )$, the mixture of the densities of the null and alternative hypothesis, also estimated from the data.

As stated in Efron et al. [51], “the advantage of the local FDR is its specificity: it provides a measure of belief in gene *i*'s 'significance' that depends on its *P*-value, not on its inclusion in a larger set of possible values” as it occurs, e.g., with *q*-values or the standard FDR. In addition, the clear statistical meaning of the local FDR [i.e., *P*(*H*_0_|*z*)] allows genes to be compared with transcripts to provide the best biomarker, taking into account whether it is a gene or a transcript. For example, in Fig. 6, transcripts are better biomarkers than genes for the first KD gene and vice versa for the last KD gene. Splitting the results into different groups increases the statistical power (as stated in [50]).

The local FDR and π_0_ were estimated using the Bioconductor R Package *qvalue* (Qvalue, RRID:SCR_001073) [52]. The value of π_0_ provides an estimate on whether transcripts or genes are better biomarkers for a particular RNAi target, as observed in Fig. 6. In addition, Fig. S5 shows different real cases in which the best biomarkers are genes or isoforms.

## Discussion

We have developed TranscriptAchilles, a large-scale tool to predict genomic biomarkers associated with gene essentiality. This is the first approach that combines high-throughput RNAi screenings with isoform expression. In addition, we have developed a methodology that combines gene and transcript expression to predict biomarkers of essentiality.

The 2 main technologies integrated in TranscriptAquilles are genome-wide loss-of-function RNAi screens and whole-transcriptome expression profiling using RNA-seq. We first discuss the potential and limitations of these technologies and then comment on the results of TranscriptAchilles.

RNAi screening provides an approach to predict genes that are essential for cell viability. Analyzing the output of these experiments is a challenge owing to the off-target effects of shRNAs, which are mainly produced by the similarity of seed sequences. Several methodologies have explicitly modeled seed effects and dramatically improved the essentiality score [13–15, 53]. In this scenario, the DEMETER score outperforms other summarization techniques. Despite the efforts made to decrease these errors, reducing the off-target effects of shRNA remains a challenge when it comes to predicting the essentiality of the gene. In fact, DEMETER's developers are further improving their tool [16]. In addition, other promising loss-of-function approaches are emerging to identify essential genes, such as genome editing through the use of CRISPR [54].

Isoforms were quantified in a previous investigation using Kallisto. It could be argued that Kallisto only detects known isoforms included in a reference transcriptome and that, in cancer, there are many novel isoforms, perhaps because of malfunctioning of the spliceosome [55]. Despite this disadvantage, isoform quantification algorithms—such as Kallisto—can be better adapted to compare disparate experiments. In addition, transcriptome annotation is ever increasing and improving, filling gaps of previous versions. Kallisto was able to identify well-expressed isoforms that, in turn, were almost perfect biomarkers of the essentiality of their companion genes. Using other algorithms, such as Stringtie [56] or Cufflinks [57, 58], we could have discovered novel isoforms. Unfortunately, the specificity and sensitivity of the transcriptome reconstruction algorithms is well below 50% [59] and computation time is much larger. In summary, novel splicing events can be a fruitful source of biomarkers, but, given the present knowledge of the transcriptome, known isoforms also present great potential as a source of biomarkers in precision medicine and are much easier to integrate.

Regarding TranscriptAchilles, the pipeline has 3 steps: (i) selecting the cohort of cell lines, (ii) finding essential genes, and (iii) predicting biomarkers. The standard use of the pipeline begins by selecting a single tumor subtype. The user can also choose a combination of tumors according to other characteristics such as histology (e.g., lung and stomach adenocarcinoma). Within this cohort, the algorithm finds genes that are essential for cell viability. Essential genes are also required to be specific for the selected cohort (when compared with the rest of the cell lines). Setting this parameter is important in order to exclude genes that, because they are essential for all cells, could be a source of adverse effects in a potential therapy.

The algorithm also predicts the best biomarkers (either genes or transcripts) of gene essentiality. We filtered the transcripts according to their expression before running the statistical model because >30% are not expressed at all in our data set. Our model integrates genes and transcripts and, with the aid of their corresponding local FDR, selects (if existing) the proper biomarker for each cancer target.

The analysis in 20 tumor subtypes suggested that the incorporation of splicing complements gene expression to find biomarkers in several cancer types. This is the case in skin carcinoma, esophagus squamous carcinoma, lung large cell carcinoma, and multiple myeloma, among others. In other tumors, such as lung adenocarcinoma, acute lymphoblastic leukemia, and colon adenocarcinoma, an analysis based merely on gene expression recalled >60% of the biomarkers. Unsurprisingly, the proportion of coding transcripts in the predicted biomarkers is higher than what is expected by chance in almost all cancer subtypes.

Finally, we showed a case study of the pipeline using kidney carcinoma cell lines. This example can easily be replicated using the application. In kidney carcinoma, 60% of essential genes were better marked by transcripts than by genes. Based on this study, the inhibition of *IRAK1* is proposed as a new potential therapeutic strategy in this tumor.

TranscriptAchilles opens a wide range of translational applications in cancer, especially in those cases that lack an effective therapy or an adequate response biomarker. Future work may exploit this powerful technique in combination with mutations, copy number variations, or chromatin modifications to find new potential drug targets with their corresponding biomarkers.

## Availability of supporting data and materials

Snapshots of the code are available in the *GigaScience* GigaDB repository [60].

## Availability of source code and requirements

Project name: TranscriptAchilles

Project home page: https://gitlab.com/fcarazo.m/transcriptachilles

http://biotecnun.unav.es:8080/app/TranscriptAchilles

- Operating systems: Platform independent
- Programming language: R
- Other requirements: RShiny, CRAN
- License: GNU GPL v3
- RRID:SCR_016849

## Additional files

Figure S1. Quick start: pipeline

Figure S2. Quick start: selection of samples

Figure S3. Quick start: essential genes

Figure S4. Quick start: prediction of transcript biomarkers

Figure S5. Three examples of TranscriptAchilles in kidney carcinoma (n = 14). In each example, the essentiality of a gene for every cell line and the log2 expression values of the gene biomarker are shown in the upper and lower plot, respectively. The cell lines are ordered according to increasing essentiality. The vertical dotted line separates the cell lines into resistant (left) and sensitive (right) to the inhibition of the essential gene (DEMETER score ≤2). Gene expression is highlighted in red. The best transcript biomarker is also highlighted. When the best biomarker is the gene, no transcript is highlighted. (A) Essentiality of *ZNF610*. The biomarker is the gene expression of *RAB17*. (B) Essentiality of *CENPU*. The best biomarker is isoform AP000275.65-003. (C) Essentiality of *IRAK1*. The isoform biomarker is not the most expressed isoform. Gene expression is not a good biomarker. However, there is a clear expression change in Isoform HSP90AA1-005.

Figure S6. (A) log2-expression box plot of the predicted transcript biomarker (SEC31A-020) in renal cancer cell lines (n = 14). *PER3* sensitive (red) and resistant (blue) cell lines are shown. (B) Expression pattern of gene *SEC31A* (red highlighted line) and its transcripts. Samples are ordered according to increasing essentiality. The black line marks the –2 essentiality threshold. The best transcript biomarker (SEC31A-020) is highlighted in blue.

Figure S7. Predicted target gene (*IRAK1*) in renal carcinoma cell lines (n = 14) with its companion biomarker (transcript MAPK1-201). (A) renal cell lines ordered by increasing essentiality of *IRAK1*. The dotted black line marks the default essentiality score of –2. (B) Expression pattern of gene *MAPK1* (red highlighted line) and its transcripts. Samples are ordered according to increasing essentiality of *IRAK1*. The dotted black line marks the –2 essentiality threshold dividing cell lines into resistant (left side) and sensitive (right side). The best transcript biomarker (MAPK1-201) is highlighted in purple. In this case, transcript expression is a better marker of essentiality than gene expression.

Figure S8. *BRAF* oncogene. Essentiality of *BRAF* for *BRAF* wt (0) and *BRAF* mut (1) in 412 samples.

Figure S9. *KRAS* oncogene. Essentiality of *KRAS* for *KRAS*wt (0) and *KRAS* mut (1) in 412 samples.

Figure S10. *NRAS* oncogene. Essentiality of *NRAS* for *NRAS* wt (0) and *NRAS* mut (1) in 412 samples.

Figure S11. *PIK3CA* oncogene. Essentiality of *PIK3CA* for *PIK3CA*wt (0) and *PIK3CA* mut (1) in 412 samples.

Figure S12. TP53 mutation and *MDM2*. Essentiality of *MDM2* for *TP53* wt (0) and *MDM2* mut (1) in 412 samples. *MDM2* is known to be essential if *TP53* is functional -*TP53* wt (0).

Figure S13. *TP53* mutation and *MDM4*. Essentiality of *MDM4* for *TP53* wt (0) and *MDM4* mut (1) in 412 samples. *MDM4* is known to be essential if *TP53* is functional -*TP53* wt (0)

## Abbreviations

AS: alternative splicing; AUC: area under the curve; CCLE: Cancer Cell Line Encyclopedia; FDR: false discovery rate; KD: knock-down; mRNA: messenger RNA; NMD: nonsense-mediated mRNA decay; RNAi: RNA interference; shRNA: short hairpin RNA; TPM: transcripts per million

## Competing interests

The authors declare that they have no competing interests.

## Funding

Research reported in this publication was supported by the Provincial Council of Gipuzkoa through the MINEDRUG project “Predicting therapy response in oncology using Big Data analysis” and the Basque Government with the grant promoting doctoral theses for young predoctoral researchers [grants PRE_2017_2_0033 to F.C. and PRE_2017_1_0327 to X.C.].

## Author contributions

Conception and design: FC, LC, XC and AR. Development of methodology: FC, LC, XC and AR. Acquisition of data: FC and AR. Development of software: FC and AR. Analysis and interpretation of data (e.g., statistical analysis, biostatistics, computational analysis): FC, LC, XC and AR. Writing, review, and/or revision of the manuscript: FC, LC, XC and AR. Development of the Shiny application: FC and AR. Study supervision: AR. All authors read and approved the final manuscript.

## Supplementary Material

GIGA-D-18-00356_Original-Submission.pdf

GIGA-D-18-00356_Revision-1.pdf

GIGA-D-18-00356_Revision-2.pdf

Response-to-Reviewer-Comments_Original-Submission.pdf

Response-to-Reviewer-Comments_Revision-1.pdf

Reviewer-1-Report-Original-Submission -- Stephen R Piccolo, Ph.D. 11/7/2018 Reviewed

Reviewer-1-Report-Revision-1 -- Stephen R Piccolo, Ph.D. 1/8/2019 Reviewed

Reviewer-2-Report-Original-Submission -- Jianjiong Gao 12/2/2018 Reviewed

Supplement_File.pdf
