# Supplementary material for: TranscriptAchilles: a genome-wide platform to predict isoform biomarkers of gene essentiality in cancer
Source: Gigascience. 2019 Apr 3;8(4):giz021. doi: 10.1093/gigascience/giz021 (PMC6446222; doi:10.1093/gigascience/giz021)
Supplement: GIGA-D-18-00356_Original-Submission.pdf [file giz021_giga-d-18-00356_original-submission.pdf]

GigaScience

TranscriptAchilles: a genome-wide platform to predict transcript biomarkers and drug target genes in cancer

--Manuscript Draft--

**Manuscript Number:**	GIGA-D-18-00356
**Full Title:**	TranscriptAchilles: a genome-wide platform to predict transcript biomarkers and drug target genes in cancer
**Article Type:**	Technical Note
**Funding Information:**	Provincial Council of Gipuzkoa (MINEDRUG project: “Predicting therapy response in oncology using Big Data analysis”) — Dr. Angel Rubio<br>Eusko Jaurlaritza (PRE_2017_2_0033) — Mr. Fernando Carazo<br>Eusko Jaurlaritza (PRE_2017_1_0327) — Mr. Xabier Cendoya
**Abstract:**	Background: Aberrant alternative splicing plays a key role in cancer development. In recent years, AS is being used as a prognosis biomarker, a therapy response biomarker and even as a therapeutic target. Next generation RNA sequencing has an unprecedented potential to measure the transcriptome. However, due to the complexity of dealing with isoforms, the scientific community has not sufficiently exploited this valuable resource in precision medicine.<br><br>Findings: We present TranscriptAchilles, the first large-scale tool to predict transcript biomarkers associated with gene inhibition sensitivity. This application integrates 412 loss-of-function RNA interference screens of over 17,000 genes together with their corresponding whole-transcriptome expression profiling. Using this tool, we have studied which are the cancer subtypes for which alternative splicing plays a significant role in terms of drug target markers. In addition, we include a case study of renal cell carcinoma that shows the biological soundness of the results. TranscriptAchilles has been developed using the Docker container framework. The databases and source code are available at GitLab (https://gitlab.com/fcarazo.m/transcriptAchilles.git/). The application is also hosted in an AWS cloud environment service (http://biotecnun.unav.es:8080/).<br><br>Conclusions: TranscriptAchilles enables the identification of putative gene targets and transcript biomarkers without the need of a bioinformatics background. This approach opens a wide range of translational applications in cancer.
**Corresponding Author:**	Angel Rubio<br><br>SPAIN
**Corresponding Author Secondary Information:**	
**Corresponding Author's Institution:**	
**Corresponding Author's Secondary Institution:**	
**First Author:**	Fernando Carazo
**First Author Secondary Information:**	
**Order of Authors:**	Fernando Carazo
	Lucia Campuzano
	Xabier Cendoya
	Francisco J. Planes
	Angel Rubio

Powered by Editorial Manager® and ProduXion Manager® from Aries Systems Corporation

Order of Authors Secondary Information:	
**Additional Information:**	
**Question**	**Response**
Are you submitting this manuscript to a special series or article collection?	No
**Experimental design and statistics**<br><br>Full details of the experimental design and statistical methods used should be given in the Methods section, as detailed in our Minimum Standards Reporting Checklist. Information essential to interpreting the data presented should be made available in the figure legends.<br><br>Have you included all the information requested in your manuscript?	Yes
**Resources**<br><br>A description of all resources used, including antibodies, cell lines, animals and software tools, with enough information to allow them to be uniquely identified, should be included in the Methods section. Authors are strongly encouraged to cite Research Resource Identifiers (RRIDs) for antibodies, model organisms and tools, where possible.<br><br>Have you included the information requested as detailed in our Minimum Standards Reporting Checklist?	Yes
**Availability of data and materials**<br><br>All datasets and code on which the conclusions of the paper rely must be either included in your submission or deposited in publicly available repositories (where available and ethically appropriate), referencing such data using a unique identifier in the references and in the "Availability of Data and Materials" section of your manuscript.	Yes

Powered by Editorial Manager® and ProduXion Manager® from Aries Systems Corporation

Have you have met the above requirement as detailed in our Minimum Standards Reporting Checklist?	

Main manuscript and supplementary materials

Click here to download Manuscript gigascience_paper_v30_e.pdf

TranscriptAchilles: a genome-wide platform to predict transcript biomarkers and drug target genes in cancer

Fernando Carazo[1], Lucía Campuzano[2], Xabier Cendoya[1], Francisco J. Planes[1] and Angel Rubio[1]*

1 Tecnun (University of Navarra), Paseo Manuel Lardizábal 15, 20018 San Sebastián, SPAIN

2 University of Luxembourg, 2, avenue de l'Université, 4365 Esch-sur-Alzette, LUXEMBOURG

** Corresponding author: Angel Rubio, e-mail: arubio@tecnun.es*

Abstract

Background: Aberrant alternative splicing plays a key role in cancer development. In recent years, AS is being used as a prognosis biomarker, a therapy response biomarker and even as a therapeutic target. Next generation RNA sequencing has an unprecedented potential to measure the transcriptome. However, due to the complexity of dealing with isoforms, the scientific community has not sufficiently exploited this valuable resource in precision medicine.

Findings: We present TranscriptAchilles, the first large-scale tool to predict transcript biomarkers associated with gene inhibition sensitivity. This application integrates 412 loss-of-function RNA interference screens of over 17,000 genes together with their corresponding whole-transcriptome expression profiling. Using this tool, we have studied which are the cancer subtypes for which alternative splicing plays a significant role in terms of drug target markers. In addition, we include a case study of renal cell carcinoma that shows the biological soundness of the results. TranscriptAchilles has been developed using the Docker container framework. The databases and source code are available at GitLab (https://gitlab.com/fcarazo.m/transcriptAchilles.git/). The application is also hosted in an AWS cloud environment service (http://biotecnun.unav.es:8080/).

Conclusions: TranscriptAchilles enables the identification of putative gene targets and transcript biomarkers without the need of a bioinformatics background. This approach opens a wide range of translational applications in cancer.

Contact: fcarazo@tecnun.es; arubio@tecnun.es

INTRODUCTION

Alternative splicing (AS) is the mechanism by which a single pre-mRNA molecule can lead to different mature mRNA molecules, called isoforms or transcripts. Through this process, a gene is capable of encoding different proteins [1]. The number of discovered isoforms increases as the study of an organism improves. In humans, around 95% of multi-exonic genes present AS events in diverse conditions [2].

AS occurs as a normal process in cells. However, there are some genetic aberrations – mutations or expression changes of splicing factor genes, among others [3] – that affect AS and may result in the expression of less standard isoforms that produce an anomalous gain or loss of protein function. AS has shown to play a pivotal role in the development of several diseases, including cancer. Specifically, all the hallmarks of cancer (e.g. angiogenesis, cell immortality, avoiding immune system response, etc.) are found to have a counterpart in aberrant splicing of key genes [4–6]. In recent years, AS is being used as a prognosis biomarker, a therapy response biomarker and even as a therapeutic target in cancer [7,8].

Several studies have analyzed the influence of AS in different contexts, as reviewed in [9]. These studies are usually based on the study of the relative or absolute concentration of transcripts looking for isoform changes across different conditions [10,11]. Since best biomarkers for a certain drug target can be either genes or isoforms, it would be desirable a methodology that integrated transcript and gene expression to provide the best biomarker regardless being a gene or a transcript.

In the context of cancer, identifying genes that are essential to cellular viability is a potential source of drug targets. Analyzing mutant phenotypes and gene repression is especially relevant to this aim. One selective and efficient way to post-transcriptionally suppress gene expression is RNA interference. Project Achilles [12] performed genome-wide RNA interference screening in different cohorts of cancer cell lines (CLs), aiming to establish cancer dependencies and essential genes. Analyzing the biological output data of these experiments has been a challenge mainly due to the off-target hybridizations of the RNAi seed sequences. The DEMETER score [13] is a statistical summarization of essentiality scores that solves this issue and outperforms other summarization scores such as the ATARiS score [14] or Bayes Factors [15]. Recently, the authors of DEMETER have published a preprint manuscript of an improved estimation of the essentiality score [16].

Different studies have successfully used Project Achilles data in combination with other -omics data to define novel personalized treatments, mainly based on mutations and copy number variations [13,14,17]. Moreover, several web-tools allow the visualization of Project Achilles data, such as Depmap (https://depmap.org/portal/). However, little work has been done to relate Project Achilles with AS.

Here, we present TranscriptAchilles, a computational genome-wide tool that exploits gene and isoform expression as biomarkers of gene essentiality in the context of cancer. It integrates loss-of-function RNA interference screening with whole-transcriptome expression profiling of 412 cancer CLs. Using this tool, we have studied which are the cancer subtypes for which AS plays a significant role in terms of drug target markers. Skin carcinoma, esophagus squamous carcinoma, lung large cell carcinoma and multiple myeloma are the most splicing-influenced cancer subtypes. In addition, we include a case study of renal cell carcinoma that shows the biological soundness of the results. TranscriptAchilles provides a user-friendly web interface to identify potential drug targets and their corresponding biomarkers.

RESULTS

TranscriptAchilles pipeline

We have developed a statistical pipeline to predict the best biomarkers (genes or transcripts) of sensitivity to a given gene knock-down. The model is based on *limma* [18] to state the probability of a gene/transcript to be differentially expressed in CLs that are sensitive to gene silencing. We have also developed an open and intuitive visual platform to allow researchers to carry out their own analyses following simple steps. The platform is presented in three main panels, as shown in Figure 1.

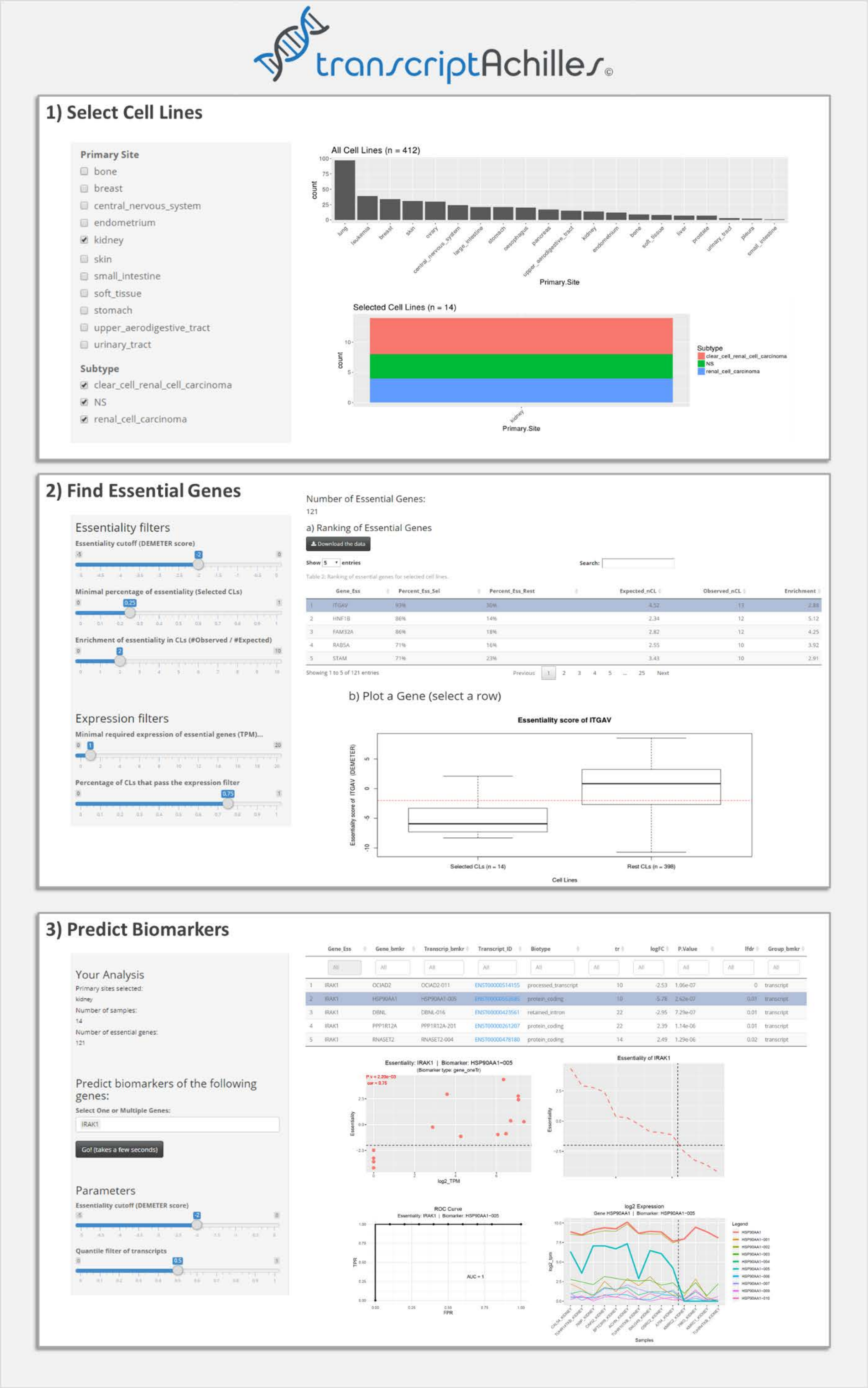
transcriptAchilles©
1) Select Cell Lines
Primary Site
bone
breast
central_nervous_system
endometrium
kidney
skin
small_intestine
soft_tissue
stomach
upper_aerodigestive_tract
urinary_tract
Subtype
clear_cell_renal_cell_carcinoma
NS
renal_cell_carcinoma
All Cell Lines (n = 412)
count
Primary.Site
Selected Cell Lines (n = 14)
Subtype
2) Find Essential Genes
Essentiality filters
Essentiality cutoff (DEMETER score)
Minimal percentage of essentiality (Selected CLs)
Enrichment of essentiality in CLs (#Observed / #Expected)
Expression filters
Minimal required expression of essential genes (TPM)...
Percentage of CLs that pass the expression filter
Number of Essential Genes:
121
a) Ranking of Essential Genes
Download the data
Showing 1 to 5 of 121 entries
b) Plot a Gene (select a row)
Essentiality score of ITGAV
Essentiality score of ITGAV (DEMETER)
Selected CLs (n = 14)
Rest CLs (n = 398)
Cell Lines
3) Predict Biomarkers
Your Analysis
Primary sites selected:
kidney
Number of samples:
14
Number of essential genes:
121
Predict biomarkers of the following genes:
Select One or Multiple Genes:
IRAK1
Go! (takes a few seconds)
Parameters
Essentiality cutoff (DEMETER score)
Quantile filter of transcripts
Essentiality: IRAK1 | Biomarker: HSP90AA1-005
Essentiality of IRAK1
log2_TPM
ROC Curve
AUC = 1
FPR
TPR
log2 Expression
Gene HSP90AA1 | Biomarker: HSP90AA1-005
log2_tpm
Samples
Legend

Figure 1. Screenshots of the three main tabs of TranscriptAchilles. ***1) Selection of cell-lines (CLs)****. Both primary site and subtypes can be selected. Two histograms summarize the number of all (up) and selected (down) CLs.* ***2) Find Essential Genes.*** *This functionality finds genes whose inhibition reduces the proliferation of the selected cohort. The returned genes are essential, specific and expressed in the selected CLs. All the parameters can be tuned with the sliders. A ranking of essential genes and a boxplot of essentiality (DEMETER score) for the selected cohort (left) and the rest of CLs (right) are shown. The red dotted line marks the default essentiality score of -2 dividing the samples into resistant (up) and sensitive (down) to the knock-down. In this case, the essential gene selected in the ranking table is ITGAV.* ***3) Predict biomarkers*** *(both transcripts and genes) for the essential genes selected by the user. This analysis can be run for every essential gene in the other tab. The ranking of biomarkers has the following columns: Gene_Ess: essential gene; Gene_bmkr and Transcript_bmkr: gene/transcript expression biomarker; tr: number of transcripts of the corresponding gene; logFC: log2 Fold change of expression; Lfdr: local fdr; Group_bmkr: indicates whether the best biomarker is a gene or a transcript. See legend of Figure 4 for a more detailed explanation of the plots.*

The main panels of the platform are:

Select CLs. The user can select the cohort of CLs to be analyzed. Several primary sites and subtypes can be selected at the same time. The application is pre-loaded with all the necessary data, so that the user does not need to upload any file.

Find Essential Genes. TranscriptAchilles identifies putative drug targets for the selected CLs. Essential genes are required to meet several criteria: 1) they must be essential for a minimum percentage of samples in the selected subtype, 2) they must be specific for the subtype under study and 3) they must be expressed. In order to achieve these three requirements, the user must set several thresholds. The first one is the percentage of cell-lines that are sensitive to the gene knockdown of interest. The second one is an odds ratio, which can be illustrated with an example: if the enrichment is set to 2, the percentage of CLs sensitive to the gene knockdown must be two times larger for the CLs under study than for the rest of CLs in the DEMETER dataset. Finally, a threshold on minimum TPM (transcripts per million) expression can be set to ensure that the gene is expressed.

Predict Biomarkers for a Target Gene. In this section, the user can select one or more genes from the previous step and predict putative biomarkers of their essentiality. The statistical model estimates the local false discovery rate (lfdr) for both genes and transcripts and decides whether genes or transcripts are the best markers for each case (see Methods section). The user can also find biomarkers for all the essential genes identified in the step *Find Essential Genes in the tab Predict Genome-Wide Biomarkers*.

Implementation and availability

TranscriptAchilles has been developed using the Docker container framework. It guarantees computational reproducibility and facilitates collaborative research. The application has been fully developed using R [19] and Shiny [20]. The databases and source code are available at GitLab (https://gitlab.com/fcarazo.m/transcriptAchilles.git/). Once the git repository is cloned, TranscriptAchilles' dependencies can be installed with the following lines of code (also included in the repository):

```
install.packages(c('shiny', 'shinyjs', 'shinythemes', 'ggplot2', 'scales',
'dply', 'rMatrix', 'matrixStats', 'tidyr', 'readr', 'psych', 'pheatmap',
'RColorBrewer', 'progress', 'grid', 'shinycssloaders', 'rmarkdown', 'DT',
'ROCR', 'plotROC'), repos='https://cloud.r-project.org/')

source('https://bioconductor.org/biocLite.R');biocLite();
biocLite(pkgs=c('impute', 'limma', 'STRINGdb', 'qvalue'))
```

Then, the shiny app can be run locally simply by typing:

```
shiny::runApp('./transcriptachilles')
```

TranscriptAchilles is also hosted using the AWS cloud environment service on the server: http://biotecnun.unav.es:8080/. The security of the app is managed by using the ShinyProxy framework [21].

Splice-based overview of tumor subtypes

We conducted several comparisons throughout 20 tumor subtypes to quantify the potential of genes and transcripts to be used as biomarkers of essentiality (Figure 2). We ran our pipeline for every tumor subtype with at least 7 samples (20 tumor subtypes). For each of them, we identified a set of genes that were essential in the selected cohort of CLs by running the *Find Essential Genes* tab (essential: DEMETER score < -2; specific: enrichment of essentiality ≥ 1; expressed: TPM > 1).

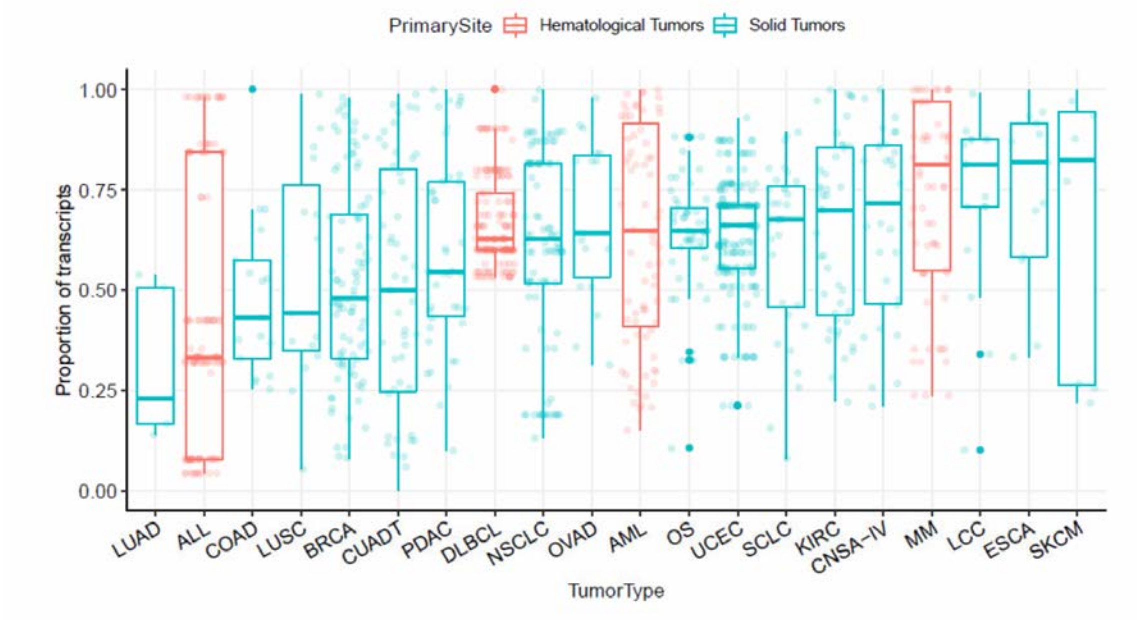

Figure 2. Percentage of transcripts as a biomarker of essential genes in 20 tumor types. Each point of the boxplots represents the proportion of transcript biomarkers for an essential gene for a given tumor type. Acronyms: acute lymphoblastic leukemia (ALL); acute myeloid leukemia (AML); breast ductal carcinoma (BRCA); central nervous system astrocytoma grade iv (CNSA-IV); colon adenocarcinoma (COAD); upper aerodigestive tract squamous cell carcinoma (CUADT); diffuse large b-cell lymphoma (DLBCL); oesophagus squamous cell carcinoma (ESCA); kidney renal clear cell carcinoma (KIRC); lung large cell carcinoma (LCC); lung adenocarcinoma (LUAD); lung squamous cell carcinoma (LUSC); multiple myeloma (MM); lung non-small cell carcinoma (NSCLC); osteosarcoma (OS); ovary adenocarcinoma (OVAD); pancreas ductal carcinoma (PDAC); lung small cell carcinoma (SCLC); skin carcinoma (SKCM) and endometrium adenocarcinoma (UCEC).

Then, we predicted a set of biomarkers related to the essentiality of the identified essential genes. As noted above, these biomarkers can be either transcripts or genes. With this information available, we compared the capability of transcripts and genes to be used as biomarkers for each tumor subtype. The underlying hypothesis is that tumors with more aberrant AS will have a larger proportion of transcript biomarkers.

Skin carcinoma, esophagus squamous carcinoma, lung large cell carcinoma and multiple myeloma are the most splicing-influenced cancer subtypes. On the other hand, for lung adenocarcinoma, acute lymphocyte leukemia and colon adenocarcinoma, isoforms have less predictive power. These findings are in accordance with a recent large-scale study of 4,542 patients from The Cancer Genome Atlas (TCGA), which measured driver and functional isoform switches in 11 cancer types [11]. Within the tumor types shared with our study, kidney

carcinoma and colon adenocarcinoma were found to have the highest and the fewest number of driver isoform switches, respectively. Lung squamous carcinoma was more affected by splicing switches than lung adenocarcinoma. In addition, we found that within hematological tumors, acute lymphoid leukemia has the lowest proportion of transcript biomarkers. On the other hand, diffuse B-cell lymphoma, acute myeloid leukemia and multiple myeloma have more than half of their essential genes better predicted by transcripts.

Considering the whole transcriptome as the source for biomarkers, we studied the recurrence of each transcript biotype of the predicted biomarkers in comparison to the general biotypes (Figure 3). Ensembl [22] catalogs transcripts into four main biotypes: protein coding, pseudogene, long noncoding and short noncoding. These four main groups contain 35 categories in total. More than 90% of the transcriptome of the 412 CLs taken together falls into 7 biotypes, namely protein coding, nonsense mediated decay, lincRNA, miRNA, antisense, processed transcript and retained intron. Protein coding transcripts is the most represented category (around 40% of transcripts).

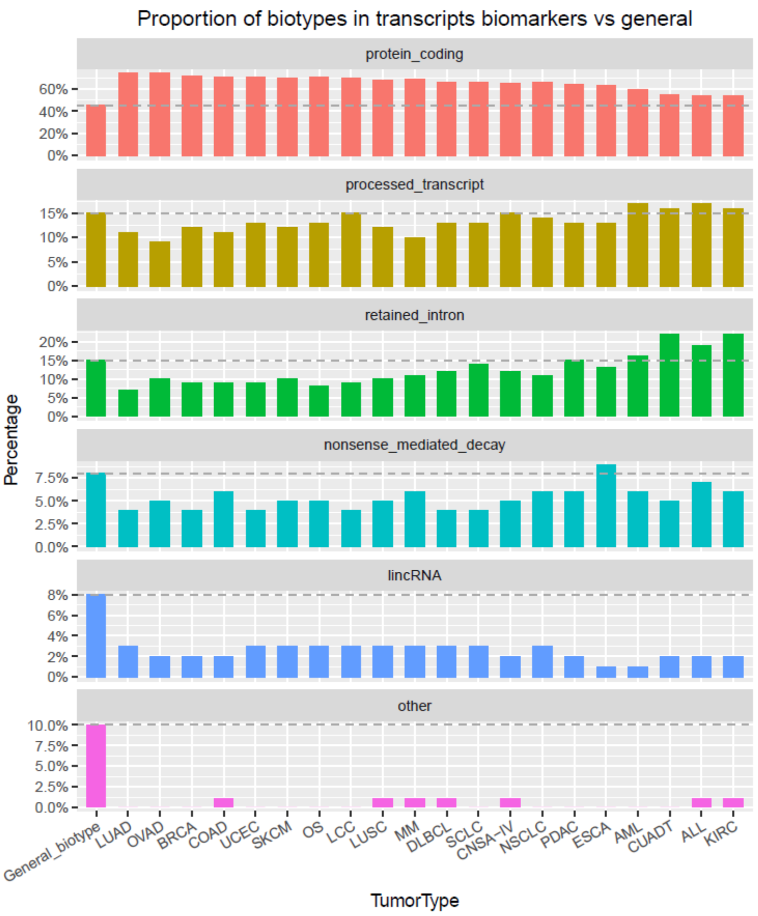

Figure 3. Proportion of transcript biotypes of biomarkers in 20 tumor types vs in general. Acronyms are included in Figure 2's caption.

We examined if the predicted biomarker biotype mimic the global distribution of biotypes in the transcriptome. Remarkably, five biotypes accounted for the vast majority of the biomarkers (Figure 3). Protein coding transcripts were the most abundant category across the 20 cell line subtypes, and tended to be overrepresented when compared to the global proportion. miRNA and other small RNAs are underrepresented in the table. This result is logical since short RNAs are usually depleted before sequencing and thus, miRNA concentration cannot be properly

measured. Intron retention is, with nonsense-mediated decay, the third most represented transcript biotype. The widespread abundance of intron retention in tumor transcriptome is well documented [23] but, to our knowledge, it has not been proposed as a possible source of biomarkers [24]. In fact, our results suggest that coding isoforms are better biomarkers. The roles of intron retention in cancer have yet to be elucidated. The primary fate of this class of AS is degradation through the nonsense-mediated mRNA decay mechanism (NMD). NMD results in reduced parent gene expression. However, it has been shown that certain intron retentions are capable of avoiding NMD and have been postulated to regulate the function of the parent gene in a dominant-negative manner [25]. Recent studies suggest that intron retentions might be a novel source of neoantigens [26]. These aberrant peptides represent ideal targets for T cell-based cancer immunotherapy and cancer vaccines (intron retention as novel source).

Case study

To further illustrate the potential of this platform in precision medicine, we show a case study using renal carcinoma CLs (n=14). We first conducted the gene essentiality analysis of these CLs. For that, we selected genes i) essential in at least 25% of renal cancer CLs, fixing the threshold for the DEMETER score as -2; ii) with a specificity odds ratio of at least 2; iii) with a minimum expression of 1TPM in at least 75% of CLs for which they were essential. Applying these parameters, 121 genes were found to be potential drug targets of renal carcinoma. Some of these genes belong to pathways known to be dysregulated in renal cancer (e.g., ITGAV, TIAM1 and PIK3CB) [27]. Interestingly, 73 out of 121 genes have previously been identified as potential cancer drivers in other tumor types in mice according to the Candidate Cancer Gene Database [28]. This proportion is significant (P-value = 1.1e-3, Fisher's exact test). Among these genes, PAX8 and HNF1B are known to play a role in renal carcinoma [29,30].

Biomarkers for essential genes were obtained by running the *"Predict Biomarkers for a Target Gene"* panel. In this case, we focused on the interleukin-1 receptor-associated kinase (*IRAK*), which is implicated in cancer initiation and progression [31]. TranscriptAchilles revealed that all the proposed biomarkers for *IRAK1* (P-value < 1e-4, |log2FC| > 2 and lfdr < 0.1) were transcripts, which stresses the importance of splicing as a source of biomarkers.

The *HSP90AA1-005* transcript is one of the best markers of *IRAK1* essentiality (Figure 4). The *HSP90* gene is known to play a role in the regulation of *IRAK1* [32]. Interestingly, while gene expression is not capable of distinguishing between sensitive and resistant groups of CLs, the predicted transcript *HSP90AA1-005* changes significantly between both groups (P-value = 2.62e-07; lfdr = 0.01).

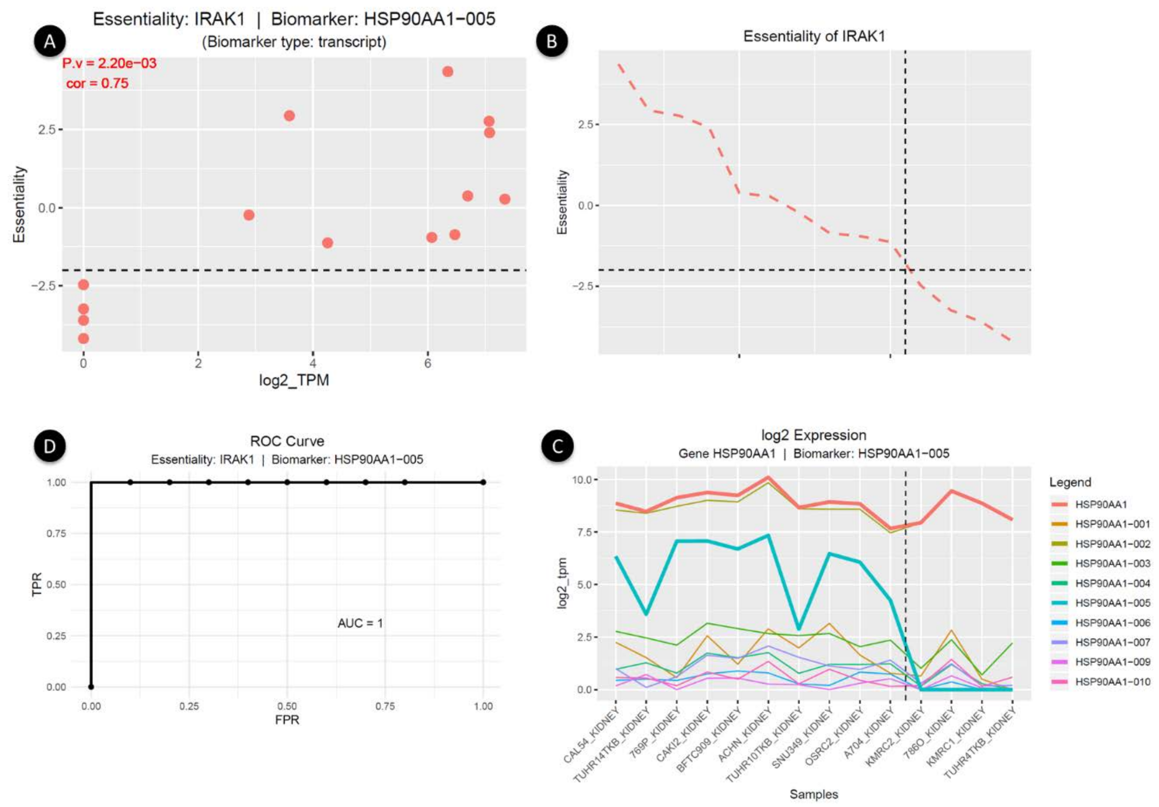

Figure 4. Output of TranscriptAchilles in renal carcinoma cell-lines (CLs, n=14). HSP90AA1-005 is a transcript biomarker of essentiality of IRAK1. ***A)*** *Scatterplot of IRAK1 essentiality and HSP90AA1-005 log2-expression. Each dot represents a single CL. The dotted black line marks the -2 essentiality threshold.* ***B)*** *Essentiality of IRAK1. Samples are sorted by its essentiality (more negative implies more essential). The black line marks the default essentiality score of -2 dividing the samples into resistant and sensitive to IRAK1 knock-down.* ***C)*** *log2-expression of gene HSP90AA1 (red line) and its transcripts. Panels B and C share the same x-axis. The dotted black line divides CLs into resistant (left side) and sensitive (right side). The best biomarker (HSP90AA1-005) is shown in blue. In this case, transcript expression provides better essentiality markers than gene expression.* ***D)*** *ROC curve of the selected biomarker. Here the AUC is 1 but this is not generally the case.*

TranscriptAchilles can also predict genome-wide biomarkers for all essential genes and rank them according to their significance. We found companion biomarkers for 101 essential genes (out of 121). In 60% of cases, the best markers were transcripts rather than genes.

Figure 4, S7 and S6 show three essential gene and biomarker pairs (IRAK1/ HSP90AA1-005, PER3/SEC31A-020, IRAK1/MAPK1-201). In these cases, transcripts are differentially expressed between sensitive and resistant CLs, whilst the corresponding genes do not show this pattern. In addition, over 95% of the proposed biomarkers for IRAK1 and PER3 were transcripts (P-value < 1e-4, |log2FC| > 2 and lfdr < 0.1).

The suggested target-biomarker pairs are biologically sound. The interleukin-1 receptor-associated kinase (IRAK) plays a key role in the toll-like receptor (TLR) and interleukin-1 receptor (IL1R) signaling pathways, which are implicated in cancer initiation and progression [31]. Mitogen-activated protein kinase (MAPK) is involved in the regulation of normal cell proliferation, survival and differentiation. Aberrant regulation of MAPK contributes to cancer through the well-studied Ras-Raf-MEK-ERK pathway [33]. The relationship between MAPK and IRAK is also documented. IRAK participates in the activation of p38 MAPK by associating with Ras [34].

METHODS

Data sources and preprocessing

The Cancer Cell Line Encyclopedia (CCLE) [35] provides public access to genomic data of near 900 cancer CLs. The transcriptome profiles of these samples were calculated in a previous study [36] from raw RNA sequencing data using Kallisto [37]. This study uses the Gencode 24 transcriptome (GRCh 38) as reference annotation [38]. This version of the transcriptome contains 199,169 transcripts. Transcript expression was measured in Transcripts Per Million (TPM) and filtered. In the filtering step, we excluded transcripts that had zero TPMs in every sample. Then, when the cohort of CLs is selected, we took the quantile 0.5 of the most expressed transcripts (this parameter can be modified within the application). After these filters, the resulting number of transcripts was around 90,000, but it depends on the selection of CLs.

In the Achilles Project 412 of these CLs were interrogated for gene essentiality using shRNA. We used the DEMETER score as a measure of essentiality [13]. DEMETER quantizes the competitive proliferation of the CLs and minimizes the effect of off-target hybridizations by using a statistical model. The more negative the DEMETER score is, the more essential the gene is for a cell line. Authors of the DEMETER score established a cut-off of -2 as a threshold of essentiality. Genes with a DEMETER score lower than this threshold can be considered essential for a cell line. Missing elements of DEMETER were imputed using the nearest neighbor averaging algorithm (KNN) [39].

Combining DEMETER and gene and isoform expression, we developed a statistical pipeline to find essential genes and predict the best markers of essentiality (Figure 5).

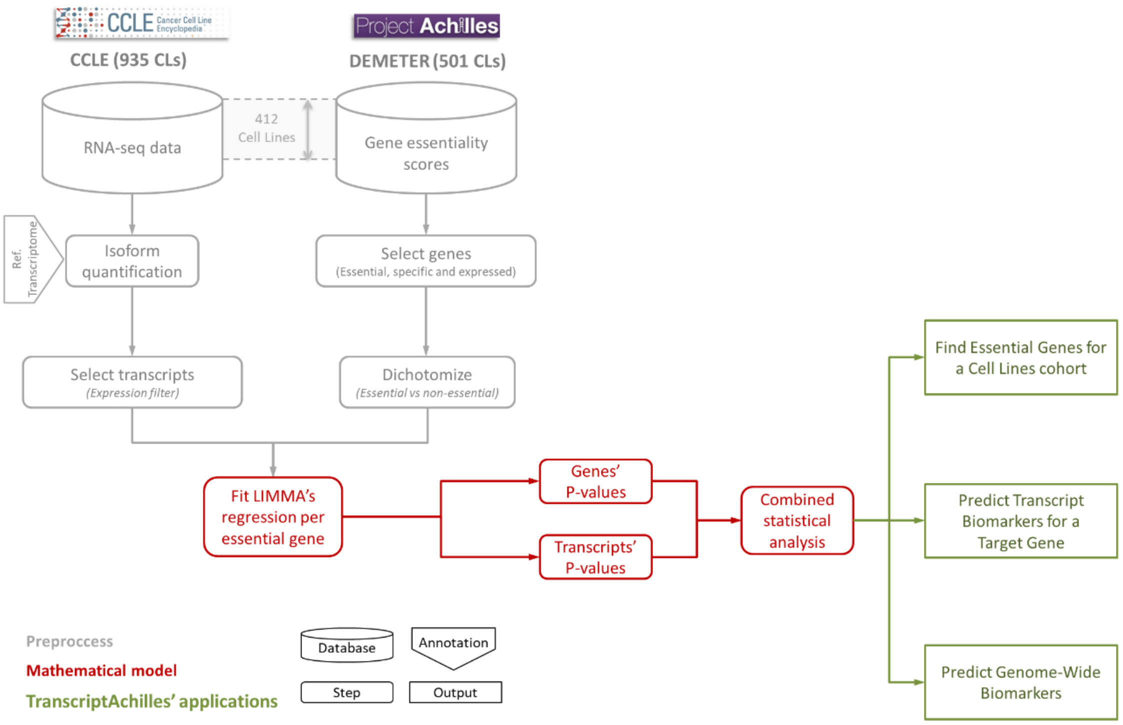

Figure 5. TranscriptAchilles' workflow. Grey database icons represent CCLE and Project Achilles data. 412 samples were matched between them. Step boxes represent algorithmic analysis, for both preprocessing (grey) and the mathematical modeling (red). Green boxes represent potential applications of TranscriptAchilles.

Mathematical model

Let n denote the number of screened samples and e denote the number of RNAi target genes. Let **D** be an $e \times n$ matrix of essentiality with each element $d_{i,j}$ representing the DEMETER score for the RNAi target *i* in sample *j*. Let **D*** be a $n \times e$ dichotomized matrix whose each element $d^*{}_{i,j}$ denotes whether sample *j* is resistant or sensitive to the RNAi target *i* as follows:

$$d^*{}_{i,j} = \begin{cases} 1, & if\ d_{i,j} < thr \quad (Essential;\ Ess) \\ 0, & otherwise \quad (non - Essential;\ \overline{Ess}) \end{cases}$$

where *thr* is a threshold whose default value is -2 as proposed in DEMETER.

Let ***s*** be a subset of *n' CLs that* yields an essentiality vector $\boldsymbol{d}^*_{\boldsymbol{e_s}} = \left(d_{e_{s_1}}, \dots, d_{e_{s_{n'}}}\right)$ for the eth RNAi target. Let $\boldsymbol{y}_{\boldsymbol{g_s}} = \left(y_{g_{s_1}}, \dots, y_{g_{s_{n'}}}\right)$ be the expression vector of a putative gene biomarker and $\boldsymbol{y}_{\boldsymbol{t_s}} = \left(y_{t_{s_1}}, \dots, y_{t_{s_{n'}}}\right)$ be an expression vector of one of their corresponding transcripts. The null hypotheses are defined as:

$$H_0^g\colon\ E\left(\boldsymbol{y}_{\boldsymbol{g_s}} \middle| \boldsymbol{d}^*_{\boldsymbol{e_s}} \in Ess\right) = E\left(\boldsymbol{y}_{\boldsymbol{g_s}} \middle| \boldsymbol{d}^*_{\boldsymbol{e_s}} \notin Ess\right)$$

$$H_0^t\colon\ E\left(\boldsymbol{y}_{\boldsymbol{t_s}} \middle| \boldsymbol{d}^*_{\boldsymbol{e_s}} \in Ess\right) = E\left(\boldsymbol{y}_{\boldsymbol{t_s}} \middle| \boldsymbol{d}^*_{\boldsymbol{e_s}} \notin Ess\right)$$

To test this hypothesis, we used a moderated t-test using *limma* [18]. We applied this test for each RNAi target using all the expressed genes and transcripts. For both genes and transcripts, we estimated the local false discovery rate (lfdr) [40]. The estimated lfdr of a given test is an empirical Bayesian posterior probability of having a true null hypothesis, conditioned on the observed p-value. The formula of the lfdr is the following:

$$lfdr(z) = \frac{\pi_0 f_0(z)}{f(z)},$$

where π_0 is the probability that a gene/transcript is not differentially expressed, $f_0(z)$ the empirical null distribution –usually an uniform (0,1) distribution for well-designed tests- and $f(z)$ the mixture of the densities of the null and alternative hypothesis, estimated from the data. In our case, the lfdr is the probability that either a gene or a transcript is not differentially expressed in sensitive and resistant CLs. To test the predictive power of genes and transcripts, we simply compared the lfdr for each one and ranked them accordingly. The followed methodology to integrate genes and transcripts P-values is similar to the IHW (Independent Hypothesis Weighting) procedure [41], a multiple testing correction that increases power while controlling the false discovery rate.

The lfdr and π_0 were estimated using the Bioconductor's R Package *qvalue* [42]. The value of π_0 gives an estimate on whether transcripts or genes are better biomarkers for a particular RNAi target, as observed in Figure 6. In addition, Figure S5 shows different real cases in which the best biomarkers are genes or isoforms.

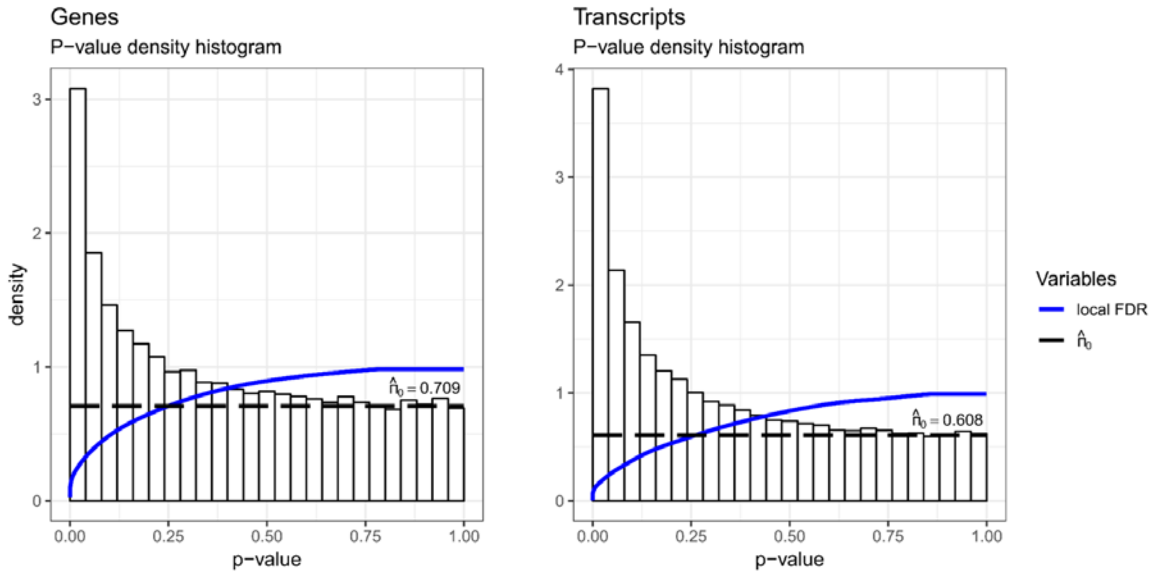

Figure 6. P-value density histogram of genes (left) and transcripts (right) as markers of essentiality of IRAK1 in kidney carcinoma. π_0 (the proportion of true null hypotheses) is smaller for transcripts (0.608) than for genes (0.709), which means that transcripts are a priori better markers than genes in this particular case.

DISCUSSION

We have developed TranscriptAchilles, a large-scale tool to predict genomic biomarkers associated with gene inhibition sensitivity. This is the first approach that combines high-throughput RNA interference screenings with isoform expression. Besides, we have developed a methodology that combines gene and transcript expression to predict biomarkers of essentiality. The application has been developed using the Docker container framework that guarantees computational reproducibility and facilitates collaborative research.

The two main sources of information integrated in TranscriptAquilles are genome-wide knock-down experiments using RNA interference and isoform expression using RNA-seq. We will discuss each of them, and later on, we will continue with the described procedure in TranscriptAchilles.

RNA interference screenings provide an approach to predict genes that are essential for cell viability. The analysis of the results of these experiments is a challenge due to the off-target effects of shRNAs, mainly because of shared seed sequences. Several methodologies have explicitly modeled seed effects and dramatically improved the essentiality score [13–15,43]. The DEMETER score outperforms other summarization techniques. Despite the efforts made to decrease these errors, reducing the off-target effects of shRNA remains a challenge when it comes to predicting the essentiality of the gene. In fact, DEMETER's developers are further improving their tool [16]. In addition, other promising loss-of-function approaches are emerging to identify essential genes, such as genome editing through the use of CRISPR-Cas9 technology [44].

In spite of the potential drawbacks of the Achilles data, we conducted several tests to confirm the reliability of DEMETER. We found that essential genes were expressed in more than 80% of cases -much more than what is expected by chance-. This fact corroborates the low proportion of artifacts in the prediction of essential genes. In addition, we checked some positive controls:

well-known driver oncogenes with activating mutations. Figure S8-S13 shows the DEMETER score for different cell lines grouped by their mutation status in KRAS, BRAS, NRAS and PIK2CA. CLs with activating mutations are sensitive to the knock-down of the activated oncogenes as expected. In addition, we checked the interactions between TP53's mutation status and MDM2 and MDM4. We confirmed in all the cases the known relationships between driver genes and mutations are in accordance with the bibliography. All these results –the expression of essential genes, positive controls and the biological soundness of the results- are good signs to trust DEMETER.

We used Kallisto to quantify annotated isoforms. It could be argued that this algorithm detects only known isoforms included in a reference transcriptome, and that in cancer, there are many novel isoforms perhaps because of malfunctioning of the spliceosome [45]. Despite this disadvantage, isoform quantification algorithms can be better adapted to compare disparate experiments. In addition, transcriptome annotation is ever increasing and the proportion of novel isoforms to be elucidated has declined. Kallisto was able to identify well-expressed isoforms that, in turn, were almost perfect biomarkers of the essentiality of their companion genes. Using other algorithms –such as Stringtie [46] or Cufflinks [47,48]- we could have discovered novel isoforms. Unfortunately, the specificity and sensitivity of the transcriptome reconstruction algorithms is well below 50% [49]. In summary, novel splicing events can be a fruitful source of biomarkers. Nevertheless, given the present knowledge of the transcriptome, known isoforms present great potential as a source of biomarkers in precision medicine.

Regarding TranscriptAchilles, we will discuss the three steps of the pipeline described before: selecting the cohort of CLs, finding essential genes and predicting biomarkers. The standard usage of the pipeline begins by selecting a single tumor subtype. The user can also choose a combination of tumors according to other characteristics such as histology (e.g. lung and stomach adenocarcinoma). Within this cohort, the algorithm finds genes that are essential for cell viability. Essential genes are also required to be specific for the selected cohort (when compared with the rest of the CLs). Setting this parameter is important to exclude genes that are essential for any cell and could be a source of strong side-effects in a potential therapy.

The algorithm finally predicts the best biomarkers (either genes or transcripts) of gene essentiality. We filtered the transcripts according to their expression before running the statistical model since more than 30% are not expressed in our dataset. Our model integrates genes and transcripts and, with the aid of their corresponding local fdr, selects (if existing) the proper biomarker for each cancer target.

The analysis throughout 20 tumor subtypes suggested that the incorporation of splicing complements gene expression to find biomarkers in several cancer types. This is the case for skin carcinoma, esophagus squamous carcinoma, lung large cell carcinoma and multiple myeloma. In other tumors (lung adenocarcinoma, acute lymphocyte leukemia and colon adenocarcinoma), an analysis based merely on gene expression recalled more than 60% of the biomarkers. Unsurprisingly, the proportion of coding transcripts in the predicted biomarkers is higher than what is expected by chance in almost all cancer subtypes.

Finally, we showed a case study of the pipeline using kidney carcinoma CLs. This example can easily be replicated using the application. In kidney carcinoma, 60% of essential genes were better marked by transcripts rather than by genes. Based on this study, the inhibition of IRAK1 is proposed as a new potential therapeutic strategy in this tumor.

In order to compare isoform expression with other potential biomarkers, we tested if there were mutations that were biomarkers of IRAK1. Mutations in EIF4E are able to predict the essentiality of IRAK1, but its statistical significance is smaller than the one of the suggested isoform (p.value = 8.10e-4 vs p.value = 2.62e-07). There are other essential genes (for example CENPU) that has no mutation markers of essentiality and the p.value for the best isoform is strongly significant (p.value = 5.06e-10). Of course, these statements do not imply that isoform biomarkers are better than mutations: for some genes it can be the other way around. However, it illustrates the potential of using isoforms as biomarkers for treatment indication.

TranscriptAchilles opens a wide range of translational applications in cancer, especially in those cases that lack an effective therapy or an adequate response biomarker. Future work may exploit this powerful technique in combination with mutations, copy number variations or chromatin modifications to find new potential drug targets with their corresponding biomarkers.

FUNDING

Research reported in this publication was supported by the Provincial Council of Gipuzkoa through the MINEDRUG project: “Predicting therapy response in oncology using Big Data analysis” and the Basque Government with the grant promoting doctoral theses for young pre-doctoral researchers [grant numbers: PRE_2017_2_0033 to FC and PRE_2017_1_0327 to XC].

ACKNOWLEDGMENTS

The authors are grateful to Fernando Carazo-Villalaín and Rafael de Miguel for their technical support on web server hosting and to María J. López for her fruitful comments on the preparation of this manuscript.

REFERENCES

1. Nilsen TW, Graveley BR. Expansion of the eukaryotic proteome by alternative splicing. Nature 2010; 463:457–63
2. Park E, Pan Z, Zhang Z, et al. The expanding landscape of alternative splicing variation in human populations. Am. J. Hum. Genet. 2018; 102:11–26
3. Sebestyén E, Singh B, Miñana B, et al. Large-scale analysis of genome and transcriptome alterations in multiple tumors unveils novel cancer-relevant splicing networks. Genome Res. 2016; 26:732–744
4. Sveen A, Kilpinen S, Ruusulehto A, et al. Aberrant RNA splicing in cancer; expression changes and driver mutations of splicing factor genes. Oncogene 2015; 35:1–15
5. Ladomery M. Aberrant alternative splicing is another hallmark of cancer. Int. J. Cell Biol. 2013; 2013:
6. Oltean S, Bates DO. Hallmarks of alternative splicing in cancer. Oncogene 2014; 33:5311–5318
7. Garcia-Blanco MA, Baraniak AP, Lasda EL. Alternative splicing in disease and therapy. Nat. Biotechnol. 2004; 22:535–546
8. Safikhani Z, Smirnov P, Thu KL, et al. Gene isoforms as expression-based biomarkers predictive of drug response in vitro. Nat. Commun. 2017; 8:
9. Carazo F, Romero JP, Rubio Á. Upstream analysis of alternative splicing: a review of computational approaches to predict context-dependent splicing factors. Brief. Bioinform. 2018; 50
10. Vitting-Seerup K, Sandelin A. The Landscape of Isoform Switches in Human Cancers. Mol. Cancer Res. 2017; 15:1206–1221
11. Climente-Gonzalez H, Porta-Pardo E, Godzik A, et al. The Functional Impact of Alternative Splicing in Cancer. 2017; 2215–2226
12. Cowley GS, Weir BA, Vazquez F, et al. Parallel genome-scale loss of function screens in 216 cancer cell lines for the identification of context-specific genetic dependencies. Sci. data 2014; 1:140035
13. Tsherniak A, Vazquez F, Montgomery PG, et al. Defining a Cancer Dependency Map. Cell 2017; 170:564–576.e16
14. Shao DD, Tsherniak A, Gopal S, et al. ATARiS: Computational quantification of gene suppression phenotypes from multisample RNAi screens. Genome Res. 2013; 23:665–678
15. Hart T, Brown KR, Sircoulomb F, et al. Measuring error rates in genomic perturbation screens: gold standards for human functional genomics. Mol. Syst. Biol. 2014; 10:733–733
16. Mcfarland JM, Ho Z V, Kugener G, et al. Improved estimation of cancer dependencies from large-scale RNAi screens using model- based normalization and data integration. bioRxiv 2018; 305656

17. Aguirre AJ, Meyers RM, Weir BA, et al. Genomic copy number dictates a gene-independent cell response to CRISPR/Cas9 targeting. Cancer Discov. 2016; 6:914–929
18. Ritchie ME, Phipson B, Wu D, et al. Limma powers differential expression analyses for RNA-sequencing and microarray studies. Nucleic Acids Res. 2015; 43:e47
19. R Development Core Team. R: a language and environment for statistical computing. [http://www.Rproject.org] 2003;
20. Chang W, Cheng J, Allaire J, et al. shiny: Web application framework for R. [http://CRAN. R-project. org/package= shiny] 2017;
21. Verbeke T, Michielssen F. ShinyProxy--open source enterprise deployment for shiny. GitHub Repos 2016;
22. Zerbino DR, Achuthan P, Akanni W, et al. Ensembl 2018. Nucleic Acids Res. 2018; 46:D754–D761
23. Dvinge H, Bradley RK. Widespread intron retention diversifies most cancer transcriptomes. Genome Med. 2015; 7:1–13
24. Xi X, Li T, Huang Y, et al. RNA Biomarkers: Frontier of Precision Medicine for Cancer. Non-Coding RNA 2017; 3:9
25. Braunschweig U, Barbosa-Morais NL, Pan Q, et al. Widespread intron retention in mammals functionally tunes transcriptomes. Genome Res. 2014; 24:1774–1786
26. Smart AC, Margolis CA, Pimentel H, et al. Intron retention as a novel source of cancer neoantigens. bioRxiv 2018; 309450
27. Liu X, Wang J, Sun G. Identification of key genes and pathways in renal cell carcinoma through expression profiling data. Kidney Blood Press. Res. 2015; 40:288–297
28. Abbott KL, Nyre ET, Abrahante J, et al. The candidate cancer gene database: A database of cancer driver genes from forward genetic screens in mice. Nucleic Acids Res. 2015; 43:D844–D848
29. Clissold RL, Hamilton AJ, Hattersley AT, et al. HNF1B-associated renal and extra-renal disease—an expanding clinical spectrum. Nat. Rev. Nephrol. 2014; 11:102–112
30. Chang A, Brimo F, Montgomery EA, et al. Use of PAX8 and GATA3 in diagnosing sarcomatoid renal cell carcinoma and sarcomatoid urothelial carcinoma. Hum. Pathol. 2013; 44:1563–1568
31. Rhyasen GW, Starczynowski DT. IRAK signalling in cancer. Br. J. Cancer 2015; 112:232–237
32. De Nardo D, Masendycz P, Ho S, et al. A central role for the Hsp90·Cdc37 molecular chaperone module in interleukin-1 receptor-associated-kinase-dependent signaling by Toll-like receptors. J. Biol. Chem. 2005; 280:9813–9822
33. Roberts PJ, Der CJ. Targeting the Raf-MEK-ERK mitogen-activated protein kinase cascade for the treatment of cancer. Oncogene 2007; 26:3291–3310
34. McDermott EP, O'Neill LAJ. Ras participates in the activation of p38 MAPK by interleukin-1 by associating with IRAK, IRAK2, TRAF6, and TAK-1. J. Biol. Chem. 2002; 277:7808–7815
35. Barretina J, Caponigro G, Stransky N, et al. The Cancer Cell Line Encyclopedia enables predictive modelling of anticancer drug sensitivity Supp. Nature 2012; 483:603–7
36. Tatlow PJ, Piccolo SR. A cloud-based workflow to quantify transcript-expression levels in public cancer compendia. Sci. Rep. 2016; 6:39259
37. Bray NL, Pimentel H, Melsted P, et al. Near-optimal probabilistic RNA-seq quantification. Nat. Biotechnol. 2016; 34:525–527
38. Harrow J, Frankish A, Gonzalez JM, et al. GENCODE: The Reference Human Genome Annotation for The ENCODE Project. Genome Res 2012; 22:1760–1774
39. Brown P, Hastie T, Tibshirani R, et al. Missing value estimation methods for DNA microarrays. Bioinformatics 2001; 17:520–525
40. Efron B, Efron B, Tibshirani R, et al. Empirical bayes method and false discovery rates for microarrays. Genet. Epidemiol. 2002; 23:70–86
41. Ignatiadis N, Klaus B, Zaugg JB, et al. Data-driven hypothesis weighting increases detection power in genome-scale multiple testing. Nat. Methods 2016; 13:577–580
42. Storey JD. A direct approach to false discovery rates. J. R. Stat. Soc. Ser. B Stat. Methodol. 2002; 64:479–498
43. Jaiswal A, Peddinti G, Akimov Y, et al. Seed-effect modeling improves the consistency of genome-wide loss-of-function screens and identifies synthetic lethal vulnerabilities in cancer cells. Genome Med. 2017; 9:51
44. Meyers RM, Bryan JG, McFarland JM, et al. Computational correction of copy number effect improves specificity of CRISPR-Cas9 essentiality screens in cancer cells. Nat. Genet. 2017; 49:1779–1784
45. Ritchie W, Granjeaud S, Puthier D, et al. Entropy measures quantify global splicing disorders in cancer. PLoS Comput. Biol. 2008; 4:1–9
46. Pertea M, Pertea GM, Antonescu CM, et al. StringTie enables improved reconstruction of a transcriptome from RNA-seq reads. Nat. Biotechnol. 2015; 33:290–295
47. Trapnell C, Williams BA, Pertea G, et al. Transcript assembly and quantification by RNA-Seq reveals unannotated transcripts and isoform switching during cell differentiation. Nat. Biotechnol. 2010; 28:511–515
48. Trapnell C, Hendrickson DG, Sauvageau M, et al. Differential analysis of gene regulation at transcript resolution with RNA-seq. Nat. Biotechnol. 2013; 31:46–53
49. Steijger T, Abril JF, Engström PG, et al. Assessment of transcript reconstruction methods for RNA-seq. Nat. Methods 2013; 10:1177–1184

Supplementary Material

TranscriptAchilles: a genome-wide platform to predict transcript biomarkers and drug target genes in cancer

Fernando Carazo[1], Lucía Campuzano[2], Xabier Cendoya[1], Francisco J. Planes[1] and Angel Rubio[1]*

1 Tecnun (University of Navarra), Paseo Manuel Lardizábal 15, 20018 San Sebastián, SPAIN

2 University of Luxembourg, 2, avenue de l'Université, 4365 Esch-sur-Alzette, LUXEMBOURG

** Corresponding author: Angel Rubio, e-mail: arubio@tecnun.es*

SECTION 1. Quick start

The page of the web-app contains an *Overview* panel with detailed information about the tool and the pipeline for performing an analysis with the app. In the *Help* panel, further details of the different features can be found. The functionalities of TranscriptAchilles are presented in a set of panels in the app. Figure S1 shows the pipeline of TranscriptAchilles.

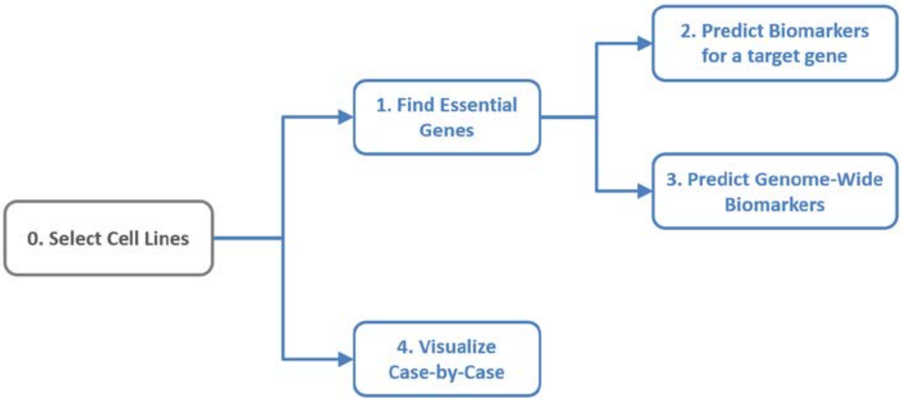

Figure S1. Quick start: pipeline

The main panels of the platform are:

0. Select CLs. The user can select the cohort of CLs to be analyzed. Several primary sites and subtypes can be selected at the same time. The application is pre-loaded with all the necessary data, so that the user does not need to upload any file.

1. Find Essential Genes. TranscriptAchilles identifies putative drug targets for the selected CLs. Essential genes are required to meet several criteria: 1) they must be essential for a minimum percentage of samples in the selected subtype, 2) they must be specific for the subtype under study and 3) they must be expressed. In order to achieve these three requirements, the user must set several thresholds. The first one is the percentage of cell-lines that are sensitive to the gene knockdown of interest. The second one is an odds ratio, which can be illustrated with an example: if the enrichment is set to 2, the percentage of CLs sensitive to the gene knockdown must be two times larger for the CLs under study than for the rest of CLs in the DEMETER dataset. Finally, a threshold on minimum TPM (transcripts per million) expression can be set to ensure that the gene is expressed.

2. Predict Biomarkers for a Target Gene. In this section, the user can select one or more genes from the previous step and predict putative biomarkers of their essentiality. The statistical model estimates the local false discovery rate (lfdr) for both genes and transcripts and decides whether genes or transcripts are the best markers for each case (see Methods section of the main manuscript).

3. Predict Genome-Wide Biomarkers. In this case the biomarkers are found for all the essential genes identified in the step *Find Essential Genes.*

4. Visualize Case-by-Case. The user can visualize the essentiality of any gene and transcript biomarker. This panel can be run once the CLs are selected.

0. Select cell lines

The user is required to select the cohort of CLs to be analyzed. Several primary sites and subtypes can be selected at the same time. The application is pre-loaded with all the necessary data, so that the user does not need to upload any data.

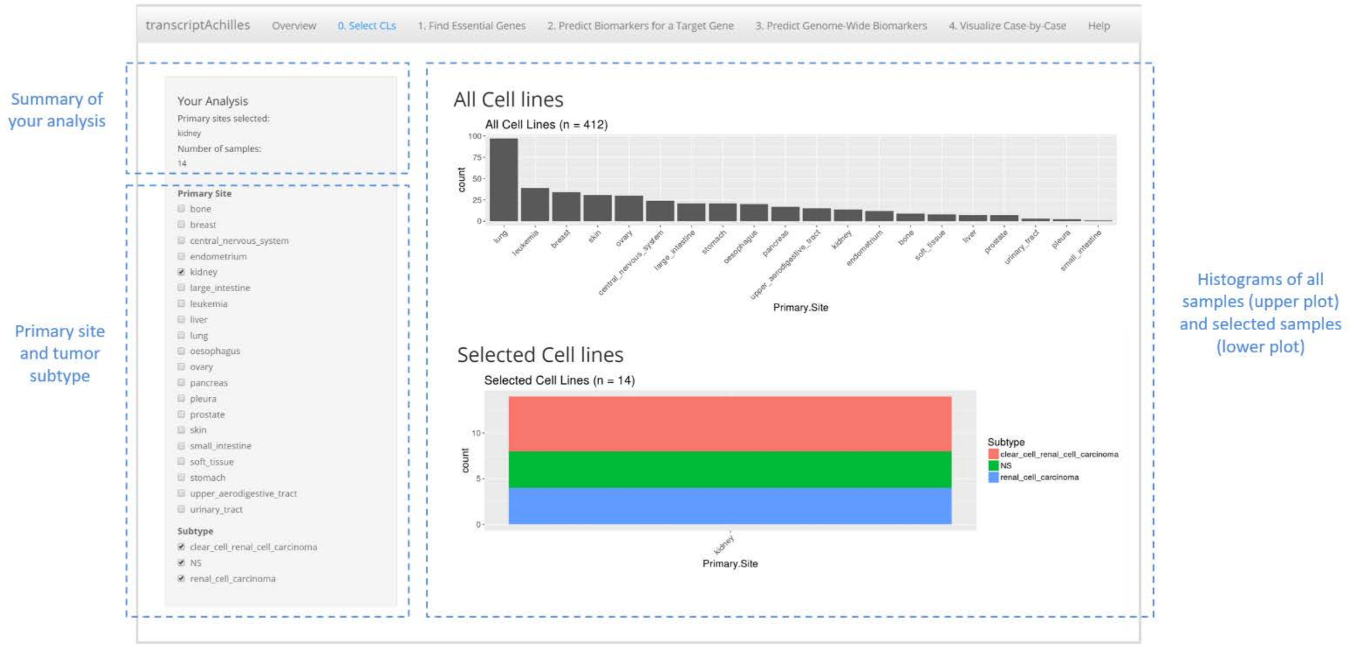

Figure S2. Quick start: selection of samples

1. Find essential genes

The second step of the analysis consists in extracting the essential gene list for the selected cell lines. Five tunable parameters allow the user to tailor the output. The toolbox provides a default value for each parameter. The assigned values correspond to our understanding of the minimum conditions which need to be satisfied by a gene to be essential.

The filters correspond to three criteria: essentiality, specificity and expression. Essentiality is a two-legged characteristic. It refers to the percentage of selected cell lines that have a DEMETER score lower than the essentiality cut-off. Specificity is represented by the enrichment ratio. This filter allows the user to define the minimum ratio between the proportion of selected cell lines for which a gene is essential and the proportion of the rest of the cell lines for which the same gene is essential.

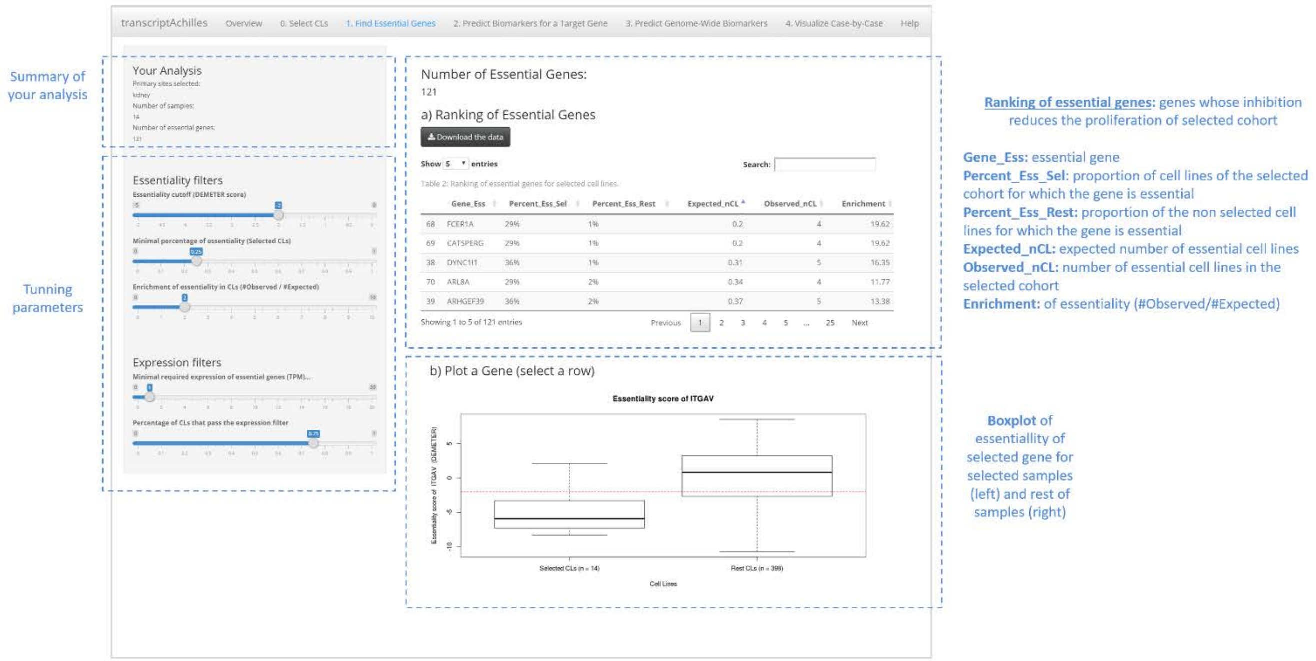

Figure S3. Quick start: essential genes

2. Predict biomarkers for a target gene

In this section, the user can select one or more genes of the previous step and predict putative biomarkers for their essentiality. In each case, the application decides whether genes or transcripts are the best markers.

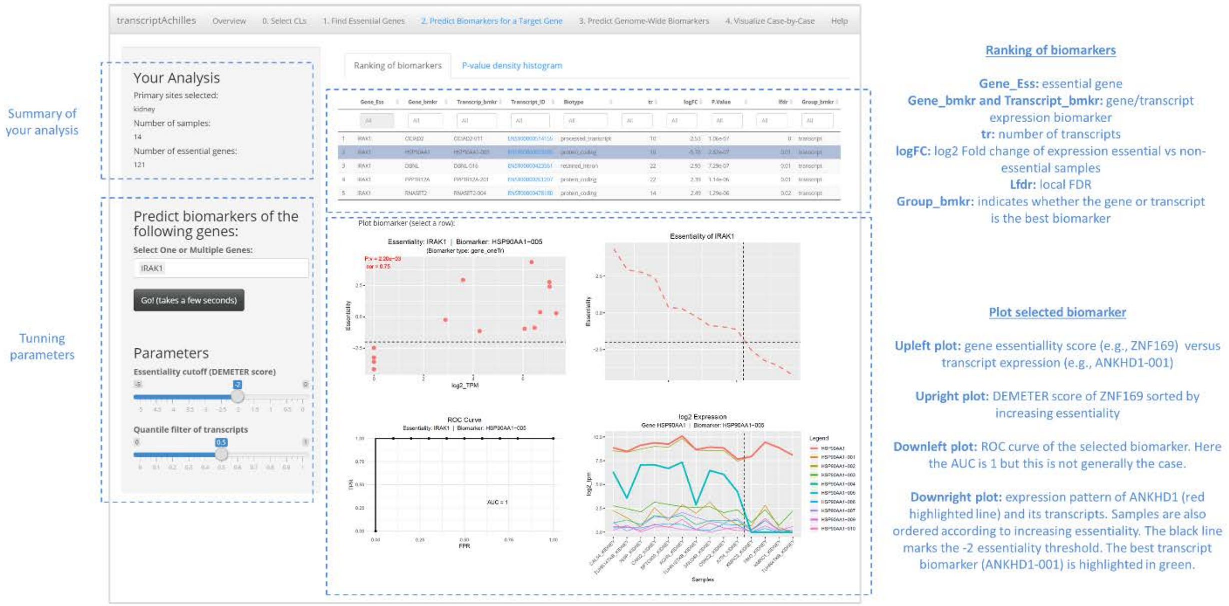

Figure S4. Quick start: prediction of transcript biomarkers

3. Predict Genome-Wide Biomarkers.

In this case the biomarkers are found for all the essential genes identified in the step *Find Essential Genes.*

4. Visualize Case-by-Case.

The user can also visualize the essentiality of any gene and transcript biomarker. This panel can be run once the CLs are selected

SECTION 2. Other examples of TranscriptAchilles

Three examples of TranscriptAchilles in kidney carcinoma:

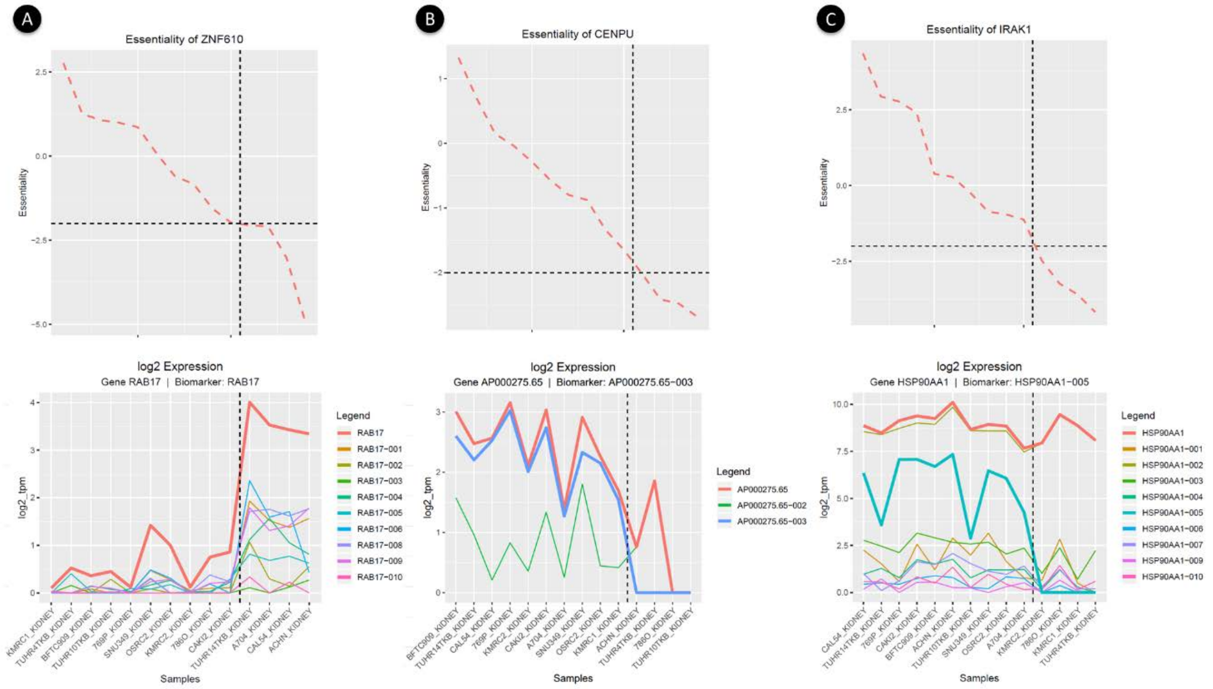

Figure S5. Three examples of TranscriptAchilles in kidney carcinoma (n = 14). In each example, the essentiality of a gene for every cell line and the log2 expression values of the gene biomarker are shown in the upper and lower plot, respectively. The cell lines are ordered according to increasing essentiality. The vertical dotted line separates the cell lines into resistant (left) and sensitive (right) to the inhibition of the essential gene (DEMETER score < -2). Gene expression is highlighted in red. The best transcript biomarker is also highlighted. When the best biomarker is the gene, no transcript is highlighted. ***A)*** *Essentiality of ZNF610. The biomarker is the gene expression of RAB17.* ***B)*** *Essentiality of CENPU. The best biomarker is isoform AP000275.65-003.* ***C)*** *Essentiality of IRAK1. The isoform biomarker is not the most expressed isoform. Gene expression is not a good biomarker. However, there is a clear expression change in Isoform HSP90AA1-005.*

Once essential genes are identified the tool allows the prediction of companion biomarkers, as none of the putative target genes is essential for 100% of renal CLs. These biomarkers (gene or transcript) are obtained by running the Predict Genome-Wide Biomarkers section of the tool.

PER3 belongs to the period circadian regulator family, which is reported to be dysregulated in kidney tumors (Mazzoccoli *et al.*, 2012). SEC31A is expressed by all renal CLs, but its transcript SEC31A-020 is absent in sensitive CLs.

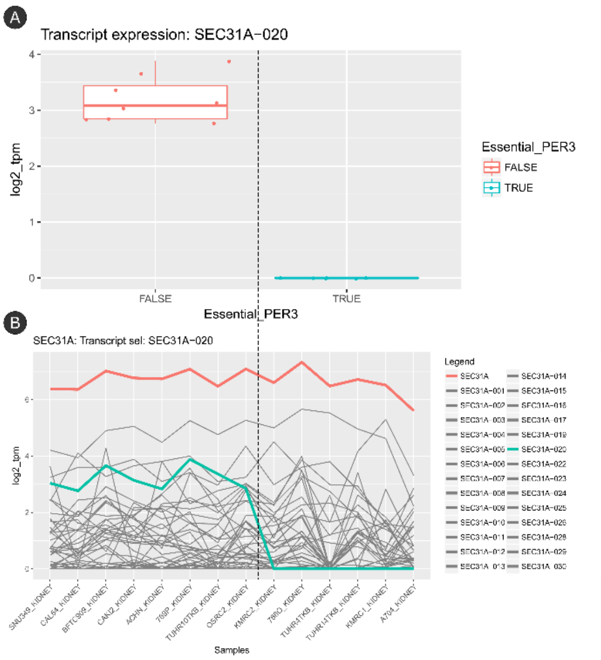

Figure S6. A) log2-expression boxplot of the predicted transcript biomarker (SEC31A-020) in renal cancer CLs (n = 14). PER3 sensitive (red) and resistant (blue) CLs are shown. B) Expression pattern of gene SEC31A (red highlighted line) and its transcripts. Samples are ordered according to increasing essentiality. The black line marks the -2 essentiality threshold. The best transcript biomarker (SEC31A-020) is highlighted in blue.

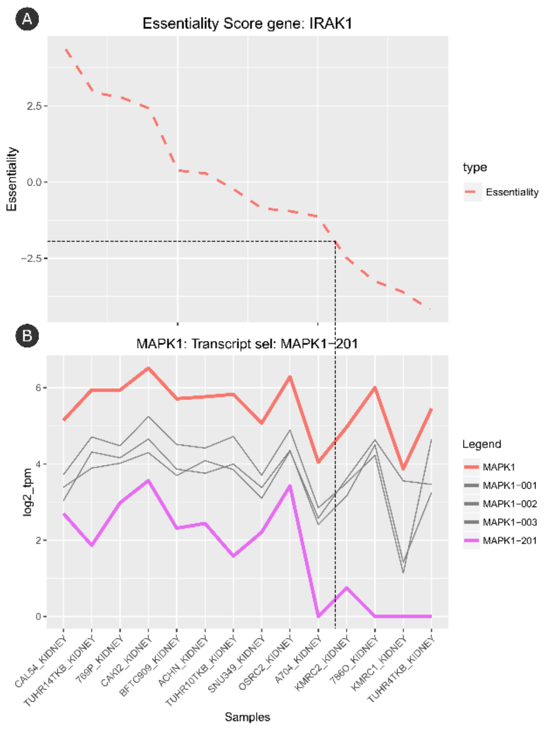

Figure S7. Predicted target gene (IRAK1) in renal carcinoma cell-lines (CLs, n = 14) with its companion biomarker (transcript MAPK1-201). A) renal CLs ordered by increasing essentiality of IRAK1.. The dotted black line marks the default essentiality score of -2. B) Expression pattern of gene MAPK1 (red highlighted line) and its transcripts. Samples are ordered according to increasing essentiality of IRAK1. The dotted black line marks the -2 essentiality threshold dividing CLs into resistant (left side) and sensitive (right side). The best transcript biomarker (MAPK1-201) is highlighted in purple. In this case, transcript expression is a better marker of essentiality than gene expression.

SECTION 3. Positive controls of DEMETER

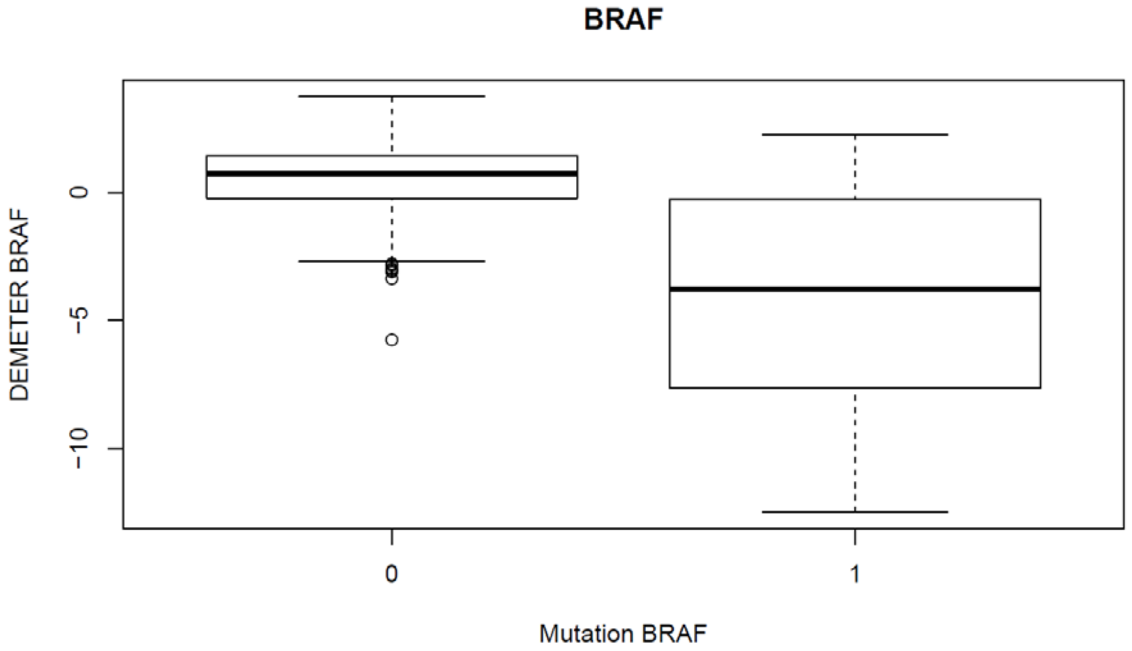

Figure S8. BRAF oncogene. Essentiality of BRAF for BRAF wt (0) and BRAF mut (1) in 412 samples.

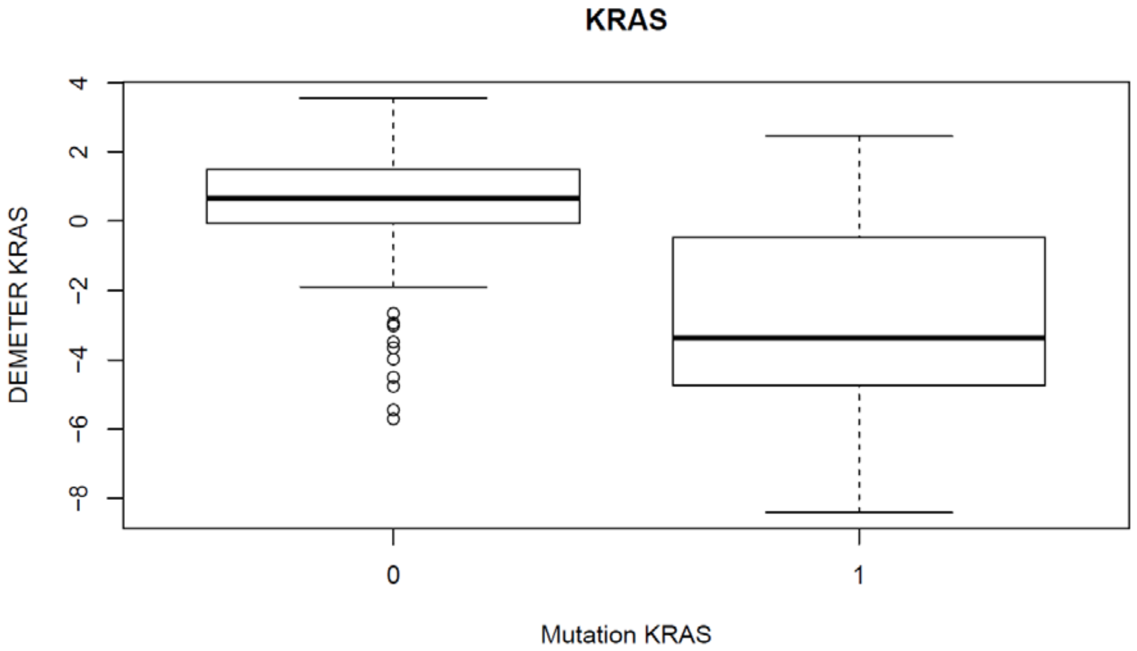

Figure S9. KRAS oncogene. Essentiality of KRAS for KRAF wt (0) and KRAS mut (1) in 412 samples.

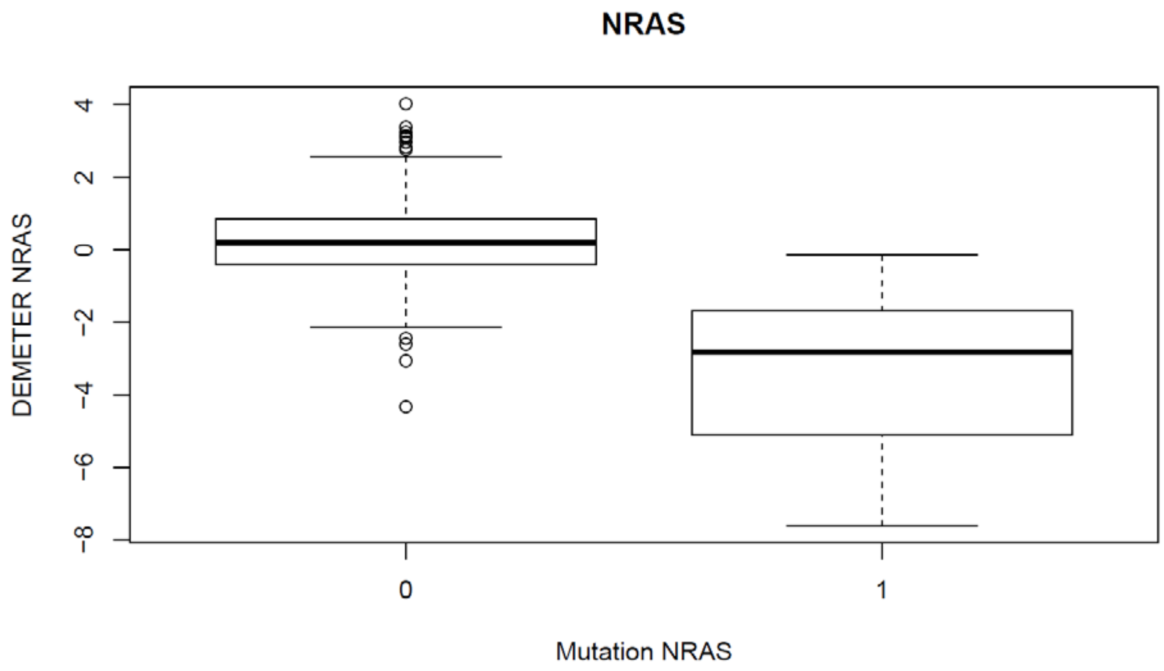

Figure S10. NRAS oncogene. Essentiality of NRAS for NRAS wt (0) and NRAS mut (1) in 412 samples.

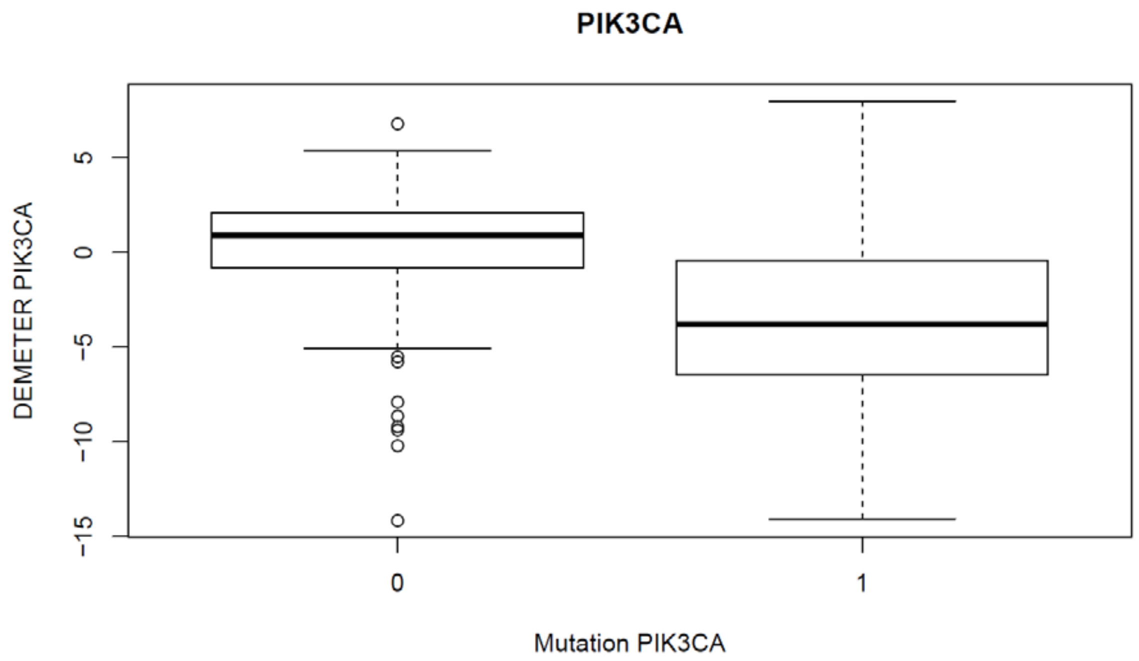

Figure S11. PIK3CA oncogene. Essentiality of PIK3CA for PIK3CA wt (0) and PIK3CA mut (1) in 412 samples.

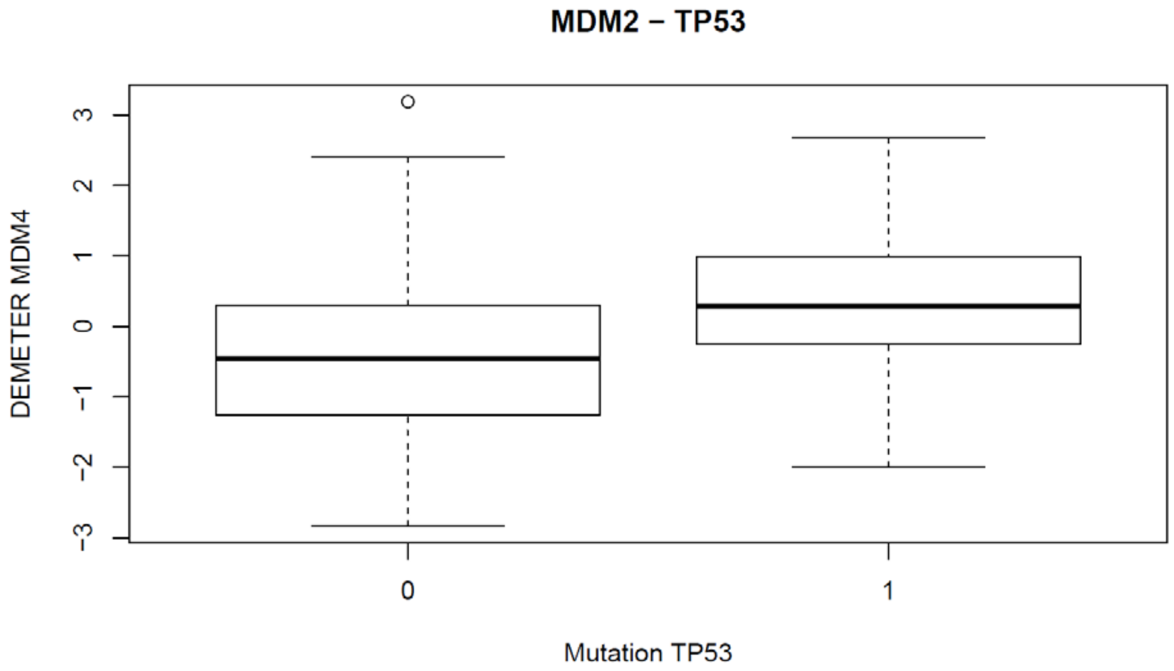

Figure S12. TP53 mutation and MDM2. Essentiality of MDM2 for TP53 wt (0) and MDM2 mut (1) in 412 samples. MDM2 is known to be essential if TP53 is functional -TP53 wt (0).

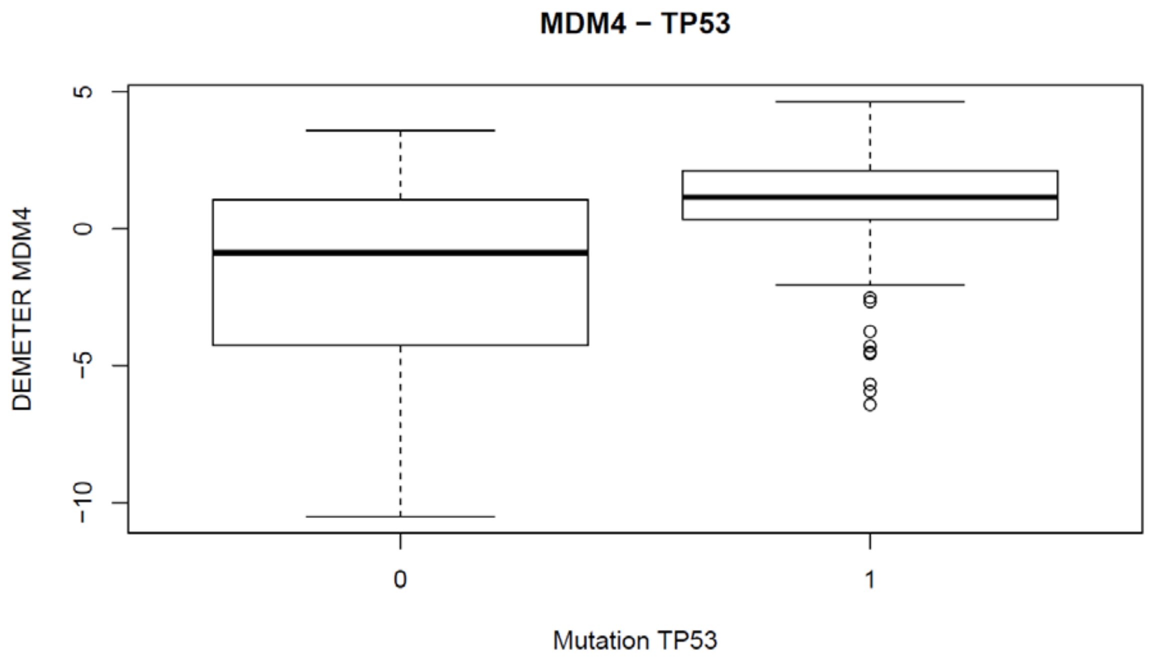

Figure S13. TP53 mutation and MDM4. Essentiality of MDM4 for TP53 wt (0) and MDM4 mut (1) in 412 samples. MDM4 is known to be essential if TP53 is functional -TP53 wt (0)

References

Barretina,J. *et al.* (2012) The Cancer Cell Line Encyclopedia enables predictive modelling of anticancer drug sensitivity Supp. *Nature*, **483**, 603–7.

Bray,N.L. *et al.* (2016) Near-optimal probabilistic RNA-seq quantification. *Nat. Biotechnol.*, **34**, 525–527.

Brown,P. *et al.* (2001) Missing value estimation methods for DNA microarrays. *Bioinformatics*, **17**, 520–525.

Efron,B. *et al.* (2002) Empirical bayes method and false discovery rates for microarrays. *Genet. Epidemiol.*, **23**, 70–86.

Harrow,J. *et al.* (2012) GENCODE: The Reference Human Genome Annotation for The ENCODE Project. *Genome Res*, **22**, 1760–1774.

Mazzoccoli,G. *et al.* (2012) Altered expression of the clock gene machinery in kidney cancer patients. *Biomed. Pharmacother.*, **66**, 175–179.

Ritchie,M.E. *et al.* (2015) Limma powers differential expression analyses for RNA-sequencing and microarray studies. *Nucleic Acids Res.*, **43**, e47.

Tatlow,P.J. and Piccolo,S.R. (2016) A cloud-based workflow to quantify transcript-expression levels in public cancer compendia. *Sci. Rep.*, **6**, 39259.

Tsherniak,A. *et al.* (2017) Defining a Cancer Dependency Map. *Cell*, **170**, 564–576.e16.
