# Supplementary material for: TranscriptAchilles: a genome-wide platform to predict isoform biomarkers of gene essentiality in cancer
Source: Gigascience. 2019 Apr 3;8(4):giz021. doi: 10.1093/gigascience/giz021 (PMC6446222; doi:10.1093/gigascience/giz021)
Supplement: GIGA-D-18-00356_Revision-2.pdf [file giz021_giga-d-18-00356_revision-2.pdf]

GigaScience

TranscriptAchilles: a genome-wide platform to predict isoform biomarkers of gene essentiality in cancer

--Manuscript Draft--

**Manuscript Number:**	GIGA-D-18-00356R2
**Full Title:**	TranscriptAchilles: a genome-wide platform to predict isoform biomarkers of gene essentiality in cancer
**Article Type:**	Technical Note
**Funding Information:**	Eusko Jaurlaritza (PRE_2017_2_0033) — Mr. Fernando Carazo<br>Eusko Jaurlaritza (PRE_2017_1_0327) — Mr. Xabier Cendoya<br>Provincial Council of Gipuzkoa (MINEDRUG project: “Predicting therapy response in oncology using Big Data analysis”) — Dr. Angel Rubio
**Abstract:**	Background<br><br>Aberrant alternative splicing (AS) plays a key role in cancer development. In recent years, AS is being used as a prognosis biomarker, a therapy response biomarker and even as a therapeutic target. Next generation RNA sequencing has an unprecedented potential to measure the transcriptome. However, due to the complexity of dealing with isoforms, the scientific community has not sufficiently exploited this valuable resource in precision medicine.<br><br>Findings<br><br>We present TranscriptAchilles, the first large-scale tool to predict transcript biomarkers associated with gene essentiality in cancer. This application integrates 412 loss-of-function RNA interference screens of over 17,000 genes together with their corresponding whole-transcriptome expression profiling. Using this tool, we have studied which are the cancer subtypes for which alternative splicing plays a significant role to state gene essentiality. In addition, we include a case study of renal cell carcinoma that shows the biological soundness of the results. The databases, the source code and a guide to build the platform within a Docker container are available at GitLab (https://gitlab.com/fcarazo.m/transcriptAchilles.git/). The application is also available online at (http://biotecnun.unav.es:8080/app/TranscriptAchilles).<br><br>Conclusions<br><br>TranscriptAchilles provides a user-friendly web interface to identify transcript or gene biomarkers of gene essentiality, which could be used as a starting point for a drug development project. This approach opens a wide range of translational applications in cancer.
**Corresponding Author:**	Angel Rubio<br><br>SPAIN
**Corresponding Author Secondary Information:**	
**Corresponding Author's Institution:**	
**Corresponding Author's Secondary Institution:**	
**First Author:**	Fernando Carazo
**First Author Secondary Information:**	
**Order of Authors:**	Fernando Carazo

Powered by Editorial Manager® and ProduXion Manager® from Aries Systems Corporation

	Lucia Campuzano
	Xabier Cendoya
	Francisco J. Planes
	Angel Rubio
**Order of Authors Secondary Information:**	
**Response to Reviewers:**	Thank you for your comments. We have included a point-by-point response to the reviewers.<br><br>“Reviewer reports:<br>Reviewer #1: The authors addressed my comments. I have a few additional comments.<br>1. Maybe I am missing it, but I could not find where I can download the data files used in the app. It is important to make these easily and openly accessible for reproducibility and for data sharing reasons.”<br><br>Thank you for pointing it out. We have added a “download data” section in the user’s guide of GitLab (https://gitlab.com/fcarazo.m/transcriptachilles/). In this section, we have included the paths where data can be downloaded including both the integrated data and the original data [1].<br><br>[1] Tatlow PJ, Piccolo SR. A cloud-based workflow to quantify transcript-expression levels in public cancer compendia. Sci Rep<br><br>“2. I was able to build the Docker container successfully. But I wasn't sure what command to run after that to start the web server. Please clarify this.”<br><br>Thanks. We have included the command to start the web server in the user’s guide of GitLab (https://gitlab.com/fcarazo.m/transcriptachilles/) and referred the reader to the “Getting Started” page of ShinyProxy to get more information about the configuration. In order to test the Docker image, the most straightforward method is to download the docker from Docker hub ("Build Docker from Docker Hub") and run the container ("Run TranscriptAchilles in a Docker App").<br><br>“3. You might consider pushing your Docker image to Docker Hub so that others don't need to build it before running it.”<br><br>Thank for the comment. We have pushed the docker image to Docker Hub and included the instructions to build it in the documentation of GitLab (https://gitlab.com/fcarazo.m/transcriptachilles/). We tested that it works in Windows, Linux Ubuntu and Mac OSX.<br><br>“Nice paper and tool!”<br><br>We really appreciated that!
**Additional Information:**	
**Question**	**Response**
Are you submitting this manuscript to a special series or article collection?	No
**Experimental design and statistics**<br><br>Full details of the experimental design and statistical methods used should be given in the Methods section, as detailed in our Minimum Standards Reporting Checklist. Information essential to interpreting the	Yes

data presented should be made available in the figure legends.<br><br>Have you included all the information requested in your manuscript?	
**Resources**<br><br>A description of all resources used, including antibodies, cell lines, animals and software tools, with enough information to allow them to be uniquely identified, should be included in the Methods section. Authors are strongly encouraged to cite Research Resource Identifiers (RRIDs) for antibodies, model organisms and tools, where possible.<br><br>Have you included the information requested as detailed in our Minimum Standards Reporting Checklist?	Yes
**Availability of data and materials**<br><br>All datasets and code on which the conclusions of the paper rely must be either included in your submission or deposited in publicly available repositories (where available and ethically appropriate), referencing such data using a unique identifier in the references and in the "Availability of Data and Materials" section of your manuscript.<br><br>Have you have met the above requirement as detailed in our Minimum Standards Reporting Checklist?	Yes

Manuscript

Click here to view linked References

Click here to access/download;Manuscript;gigascience_paper_v38_reviewe

TranscriptAchilles: a genome-wide platform to predict isoform biomarkers of gene essentiality in cancer

Fernando Carazo[1], Lucía Campuzano[2], Xabier Cendoya[1], Francisco J. Planes[1] and Angel Rubio[1]*

1 Tecnun (University of Navarra), Paseo Manuel Lardizábal 15, 20018 San Sebastián, SPAIN

2 University of Luxembourg, 2, avenue de l'Université, 4365 Esch-sur-Alzette, LUXEMBOURG

** Corresponding author: Angel Rubio, e-mail: arubio@tecnun.es*

ORCID IDs:

Fernando Carazo: 0000-0002-4288-3652; , Lucía Campuzano: 0000-0002-8026-9610; *Xabier Cendoya*: 0000-0001-8401-4087; Francisco J Planes: 0000-0003-1155-3105; Angel Rubio: 0000-0002-3274-2450

Abstract

Background: Aberrant alternative splicing (AS) plays a key role in cancer development. In recent years, AS is being used as a prognosis biomarker, a therapy response biomarker and even as a therapeutic target. Next generation RNA sequencing has an unprecedented potential to measure the transcriptome. However, due to the complexity of dealing with isoforms, the scientific community has not sufficiently exploited this valuable resource in precision medicine.

Findings: We present TranscriptAchilles, the first large-scale tool to predict transcript biomarkers associated with gene essentiality in cancer. This application integrates 412 loss-of-function RNA interference screens of over 17,000 genes together with their corresponding whole-transcriptome expression profiling. Using this tool, we have studied which are the cancer subtypes for which alternative splicing plays a significant role to state gene essentiality. In addition, we include a case study of renal cell carcinoma that shows the biological soundness of the results. The databases, the source code and a guide to build the platform within a Docker container are available at GitLab (https://gitlab.com/fcarazo.m/transcriptAchilles.git/). The application is also available online at (http://biotecnun.unav.es:8080/app/TranscriptAchilles).

Conclusions: TranscriptAchilles provides a user-friendly web interface to identify transcript or gene biomarkers of gene essentiality, which could be used as a starting point for a drug development project. This approach opens a wide range of translational applications in cancer.

Contact: fcarazo@tecnun.es; arubio@tecnun.es

INTRODUCTION

Alternative splicing (AS) is the mechanism by which a single pre-mRNA molecule can lead to different mature mRNA molecules, called isoforms or transcripts. Through this process, a gene is capable of encoding different proteins [1]. The number of discovered isoforms increases as the study of an organism improves. In humans, around 95% of multi-exonic genes present AS events in diverse conditions [2].

AS occurs as a normal process in cells. However, there are some genetic aberrations –such as mutations or expression changes of splicing factor genes [3]– that affect AS and may result in the expression of less standard isoforms that produce an anomalous gain or loss of protein function. AS has shown to play a pivotal role in the development of several diseases, including cancer. Specifically, all the hallmarks of cancer (e.g. angiogenesis, cell immortality, avoiding immune system response, etc.) are found to have a counterpart in aberrant splicing of key genes [4–6]. In recent years, AS is being used as a prognosis biomarker, a therapy response biomarker and even as a therapeutic target in cancer [7,8].

Several studies have analyzed the influence of AS in different contexts, as reviewed in [9]. These studies are usually based on the study of the relative or absolute concentration of transcripts looking for isoform changes across different conditions [10,11]. Since the best biomarkers for a certain condition can be either genes or isoforms, it would be desirable to develop a methodology that integrated transcript and gene expression to provide the best biomarkers regardless of them being a gene or a transcript.

In the context of cancer, identifying genes that are essential to cellular viability is a potential source of drug targets. Analyzing mutant phenotypes and gene repression is especially relevant to this aim. One selective and efficient way to post-transcriptionally suppress gene expression is RNA interference. Project Achilles [12] performed genome-wide RNA interference screening in different cohorts of cancer cell lines, aiming to establish cancer dependencies and essential genes. Analyzing the biological output data of these experiments has been a challenge mainly due to the off-target hybridizations of the RNAi seed sequences. The DEMETER score [13] is a statistical summarization of essentiality scores that quantizes the competitive proliferation of the cell lines and minimizes the effect of off-target hybridizations by using a statistical model. DEMETER outperforms other summarizations such as the ATARiS score [14] or Bayes Factors [15]. Recently, the authors of DEMETER have published a preprint manuscript of an improved estimation of the essentiality score [16].

Different studies have successfully used Project Achilles data in combination with other -omics data to define novel personalized treatments, mainly based on mutations and copy number variations [13,14,17]. Moreover, several web-tools allow the visualization of Project Achilles data, such as Depmap (https://depmap.org/portal/). However, little work has been done to relate Project Achilles with AS.

Here, we present TranscriptAchilles (http://biotecnun.unav.es:8080/app/TranscriptAchilles), a computational genome-wide tool that exploits gene and isoform expression as biomarkers of gene essentiality in the context of cancer. It integrates loss-of-function RNA interference screening with whole-transcriptome expression profiling of 412 cancer cell lines. Using this tool, we have studied which are the cancer subtypes for which alternative splicing plays a significant role to state gene essentiality. In addition, we include a case study of renal cell carcinoma that shows the biological soundness of the results. This approach opens a wide range of translational applications in cancer.

RESULTS

TranscriptAchilles pipeline

We have developed a statistical pipeline to predict the best biomarkers (genes or transcripts) of gene essentiality. The model is based on *limma* [18] to state the probability of a gene/transcript to be differentially expressed in cell lines that are sensitive to gene silencing.

TranscriptAchilles uses the essentiality score of DEMETER. The DEMETER score quantizes the competitive proliferation of the cell lines and minimizes the effect of off-target hybridizations by using a statistical model. The more negative the DEMETER score is, the more essential the gene is for a cell line. Authors of the DEMETER score established a cut-off of -2 as a threshold of essentiality. Genes with a DEMETER score lower than this threshold can be considered essentials for a cell line.

Although DEMETER's authors performed some validations of their essentiality score, we did two simple tests to confirm its reliability. First, we checked that genes are expressed when they are essentials (DEMETER score < -2). We found that genes are expressed more than 1 TPM in 85% of the cases when they are essential, versus 70% when they are non-essential (Wilcoxon test p-value < 2.2 e-16).

Second, we checked the essentiality scores of some well-known driver oncogenes related to their mutational state. Figure S8-S13 shows the DEMETER score for different cell lines grouped by their mutation status in *KRAS*, *BRAF*, *NRAS* and *PIK2CA*. We found that mutated cell lines are sensitive to the knock-down of the activated oncogenes –this effect is known as "oncogene addiction" [19]. We also checked that the mutation status of *TP53* affects the essentiality of *MDM2* and *MDM4* as expected, since *MDM4* and *MDM2* regulate the activity and the stability of *TP53* respectively [20]. We confirmed, in all the cases, that the relationships between DEMETER and mutation status are in accordance with the bibliography.

In addition, we have developed an open and intuitive visual platform to allow researchers to perform their own analysis following simple steps. The platform is presented in three main panels, as shown in Figure 1.

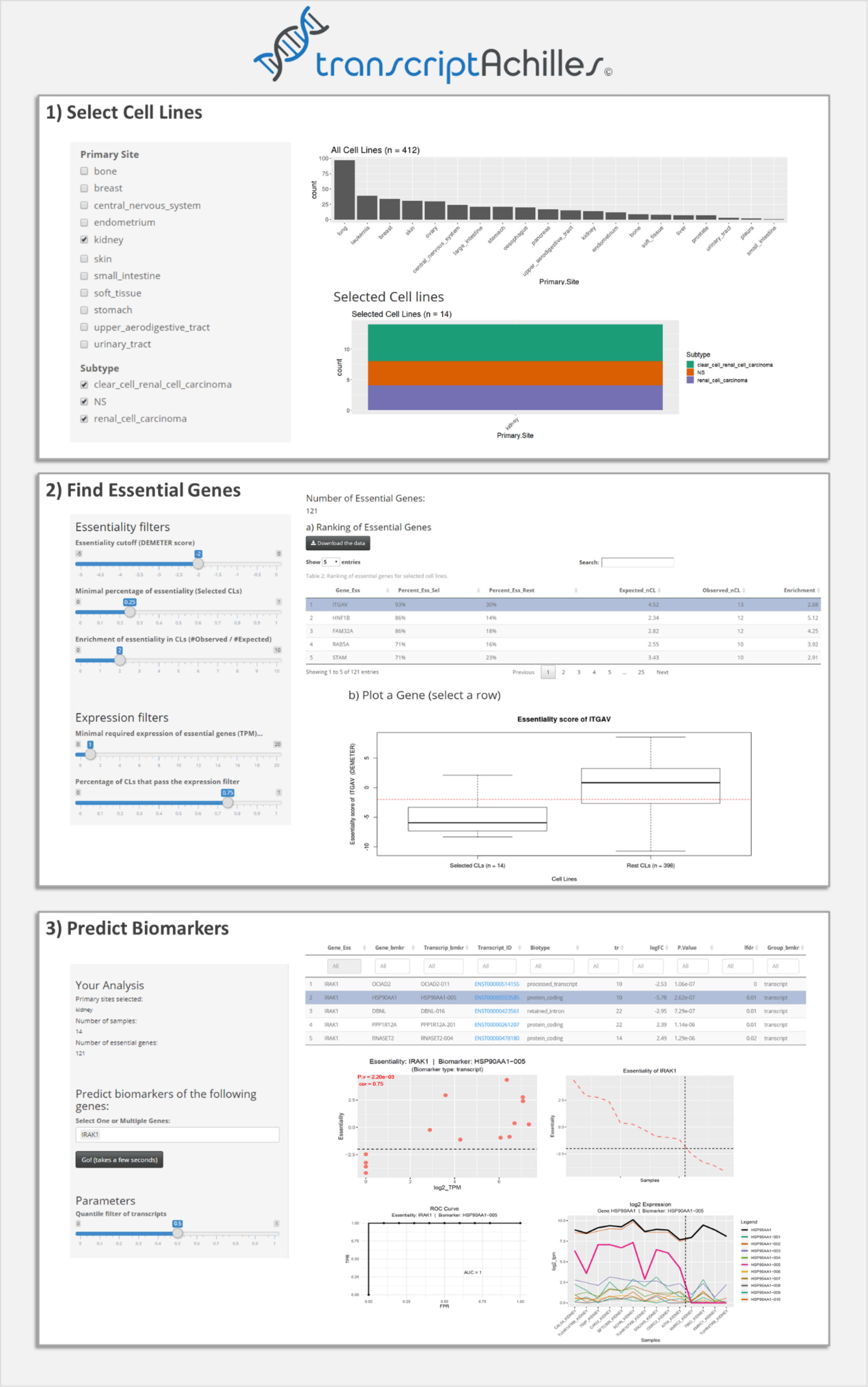

transcriptAchilles©
1) Select Cell Lines
Primary Site
bone
breast
central_nervous_system
endometrium
kidney
skin
small_intestine
soft_tissue
stomach
upper_aerodigestive_tract
urinary_tract
Subtype
clear_cell_renal_cell_carcinoma
NS
renal_cell_carcinoma
All Cell Lines (n = 412)
count
Primary.Site
Selected Cell lines
Selected Cell Lines (n = 14)
Subtype
clear_cell_renal_cell_carcinoma
NS
renal_cell_carcinoma
2) Find Essential Genes
Essentiality filters
Essentiality cutoff (DEMETER score)
Minimal percentage of essentiality (Selected CLs)
Enrichment of essentiality in CLs (#Observed / #Expected)
Expression filters
Minimal required expression of essential genes (TPM)...
Percentage of CLs that pass the expression filter
Number of Essential Genes:
121
a) Ranking of Essential Genes
Download the data
Show 5 entries
Search:
Table 2: Ranking of essential genes for selected cell lines.
Gene_Ess Percent_Ess_Sel Percent_Ess_Rest Expected_nCL Observed_nCL Enrichment
1 ITGAV 93% 30% 4.52 13 2.88
2 HNF1B 86% 14% 2.34 12 5.12
3 FAM32A 86% 18% 2.82 12 4.25
4 RAB5A 71% 16% 2.55 10 3.92
5 STAM 71% 23% 3.43 10 2.91
Showing 1 to 5 of 121 entries
Previous 1 2 3 4 5 ... 25 Next
b) Plot a Gene (select a row)
Essentiality score of ITGAV
Essentiality score of ITGAV (DEMETER)
Selected CLs (n = 14)
Rest CLs (n = 398)
Cell Lines
3) Predict Biomarkers
Your Analysis
Primary sites selected:
kidney
Number of samples:
14
Number of essential genes:
121
Predict biomarkers of the following genes:
Select One or Multiple Genes:
IRAK1
Go! (takes a few seconds)
Parameters
Quantile filter of transcripts
Gene_Ess Gene_bmkr Transcrip_bmkr Transcript_ID Biotype tr logFC P.Value fdr Group_bmkr
1 IRAK1 OCIAD2 OCIAD2-011 ENST00000514155 processed_transcript 10 -2.53 1.06e-07 0 transcript
2 IRAK1 HSP90AA1 HSP90AA1-005 ENST00000553585 protein_coding 10 -5.78 2.62e-07 0.01 transcript
3 IRAK1 DBNL DBNL-016 ENST00000423561 retained_intron 22 -2.95 7.29e-07 0.01 transcript
4 IRAK1 PPP1R12A PPP1R12A-201 ENST00000261207 protein_coding 22 2.39 1.14e-06 0.01 transcript
5 IRAK1 RNASET2 RNASET2-004 ENST00000478180 protein_coding 14 2.49 1.29e-06 0.02 transcript
Essentiality: IRAK1 | Biomarker: HSP90AA1-005
(Biomarker type: transcript)
P.v = 2.20e-03
cor = 0.75
Essentiality
log2_TPM
Essentiality of IRAK1
Samples
ROC Curve
Essentiality: IRAK1 | Biomarker: HSP90AA1-005
AUC = 1
TPR
FPR
log2 Expression
Gene HSP90AA1 | Biomarker: HSP90AA1-005
log2_tpm
Legend
HSP90AA1
HSP90AA1-001
HSP90AA1-002
HSP90AA1-003
HSP90AA1-004
HSP90AA1-005
HSP90AA1-006
HSP90AA1-007
HSP90AA1-008
HSP90AA1-009
HSP90AA1-010
Samples

Figure 1. Screenshots of the three main tabs of TranscriptAchilles. ***1) Selection of cell lines****. Both primary site and subtypes can be selected. Two histograms summarize the number of all (up) and selected (down) cell lines.* ***2) Find Essential Genes.*** *This functionality finds genes whose inhibition reduces the proliferation of the selected cohort. The returned genes are essential, specific and expressed in the selected cell lines. All the parameters can be tuned with the sliders. A ranking of essential genes and a boxplot of essentiality (DEMETER score) for the selected cohort (left) and the rest of cell lines(right) are shown. The red dotted line marks the default essentiality score of -2 dividing the samples into resistant (up) and sensitive (down) to the knock-down. In this case, the essential gene selected in the ranking table is ITGAV.* ***3) Predict biomarkers*** *(both transcripts and genes) for the essential genes selected by the user. This analysis can be run for every essential gene in the other tab. The ranking of biomarkers has the following columns: Gene_Ess: essential gene; Gene_bmkr and Transcript_bmkr: gene/transcript expression biomarker; tr: number of transcripts of the corresponding gene; logFC: log2 Fold change of expression; Lfdr: local false discovery rate; Group_bmkr: indicates whether the best biomarker is a gene or a transcript. See legend of Figure 4 for a more detailed explanation of the plots.*

The main panels of the platform are:

Select cell lines. The user can select the cohort of cell lines to be analyzed. Several primary sites and subtypes can be selected at the same time. The application is pre-loaded with all the necessary data, so that the user does not need to upload any file.

Find Essential Genes. Based on the Achilles Project data, TranscriptAchilles identifies essential genes for the selected cell lines. These genes are required to meet several criteria: 1) they must be essential for a minimum percentage of samples in the selected subtype, 2) they must be specific for the subtype under study and 3) they must be expressed. In order to achieve these three requirements, the user can tune several thresholds. The first one is the percentage of cell lines that are sensitive to the gene knockdown of interest. The second one is an odds ratio, which can be illustrated with an example: if the enrichment is set to 2, the percentage of cell lines sensitive to the gene knockdown must be two times larger for the cell lines under study than for the rest of cell lines in the DEMETER dataset. Finally, a threshold on expression can be set to ensure that the genes are expressed when they are essential.

Predict Biomarkers for a Target Gene. In this section, the user can select one or more genes from the previous step and predict putative biomarkers of their essentiality. The statistical model estimates the local false discovery rate for both genes and transcripts and decides whether genes or transcripts are the best markers for each case (see Methods section). The user can also find biomarkers for all the essential genes identified by running the panel: *Find Essential Genes in the tab Predict Genome-Wide Biomarkers*.

Implementation and availability

TranscriptAchilles (SciCrunch.org RRID: SCR_016849) has been fully developed using R [21] and Shiny [22]. The databases and source code are available at GitLab (https://gitlab.com/fcarazo.m/transcriptAchilles.git/). Once the git repository is cloned, TranscriptAchilles can be run locally following the instructions included in the repository. The application can also be run locally within Docker to avoid installation problems and to facilitate reproducibility. TranscriptAchilles is hosted using the Amazon Web Services cloud environment service on the server: http://biotecnun.unav.es:8080/app/TranscriptAchilles. The security of the app is managed by using the ShinyProxy framework [23].

Splice-based overview of tumor subtypes

We conducted several comparisons throughout 20 tumor subtypes to quantify the potential of genes and transcripts to be used as biomarkers of essentiality. We ran our pipeline for every tumor subtype with at least 7 samples (20 tumor subtypes). For each of them, we identified a

set of genes that are essential in the selected cohort of cell lines by running the Find Essential Genes tab (essential: DEMETER score < -2; specific: enrichment of essentiality ≥ 1; expressed: TPM > 1).

Using our statistical pipeline, we predicted which genes or transcripts are potential biomarkers of gene essentiality. A condition affected by splicing is more likely to have more transcripts biomarkers than one with no splicing changes. To estimate this characteristic, we compared the proportion of genes/transcripts relative to the total number of predicted biomarkers (i.e., if a certain essential gene has 10 potential biomarkers, and 9 out of which are "transcripts", we would say that 90% of its biomarkers are transcripts) (Figure 2).

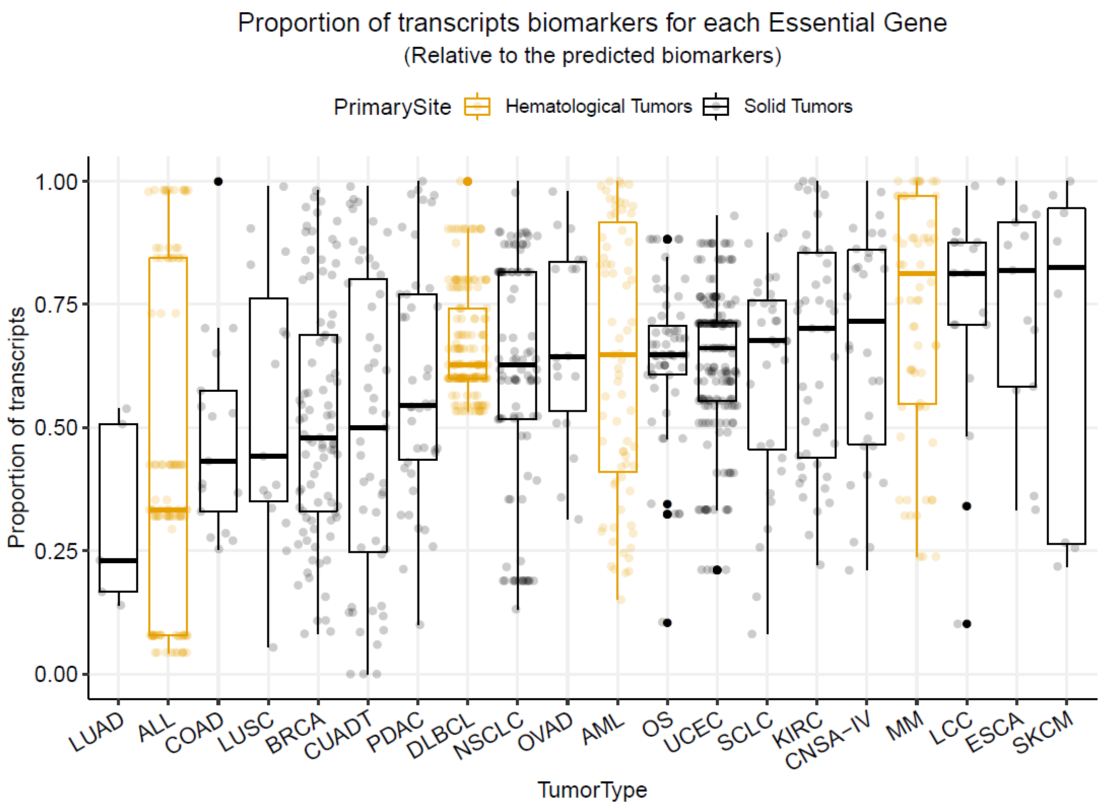

Figure 2. Percentage of transcripts predicted to be biomarkers of essential genes in 20 tumor types. Each essential gene has different biomarkers: some of them are genes and others are transcripts. Each point of the boxplots represents the proportion of transcript biomarkers for an essential gene for a given tumor type. Acronyms: acute lymphoblastic leukemia (ALL); acute myeloid leukemia (AML); breast ductal carcinoma (BRCA); central nervous system astrocytoma grade iv (CNSA-IV); colon adenocarcinoma (COAD); upper aerodigestive tract squamous cell carcinoma (CUADT); diffuse large b-cell lymphoma (DLBCL); oesophagus squamous cell carcinoma (ESCA); kidney renal clear cell carcinoma (KIRC); lung large cell carcinoma (LCC); lung adenocarcinoma (LUAD); lung squamous cell carcinoma (LUSC); multiple myeloma (MM); lung non-small cell carcinoma (NSCLC); osteosarcoma (OS); ovary adenocarcinoma (OVAD); pancreas ductal carcinoma (PDAC); lung small cell carcinoma (SCLC); skin carcinoma (SKCM) and endometrium adenocarcinoma (UCEC).

Differences found within the different tumor types were strongly significant (Kruskal-Wallis p-value = 3.18E-16). Skin carcinoma, esophagus squamous carcinoma, lung large cell carcinoma and multiple myeloma are the most splicing-influenced cancer subtypes. On the other hand, isoforms have less predictive power in lung adenocarcinoma, acute lymphocyte leukemia and colon adenocarcinoma. These findings are in accordance with a recent large-scale study of 4,542 patients from The Cancer Genome Atlas (TCGA), which measured driver and functional isoform switches in 11 cancer types [11]. Within the tumor types shared with our study, kidney

carcinoma and colon adenocarcinoma were the cancers with the highest and the fewest number of driver isoform switches, respectively. Lung squamous carcinoma was more affected by splicing switches than lung adenocarcinoma. In addition, we found that within hematological tumors, acute lymphoid leukemia has the lowest proportion of transcript biomarkers. Diffuse B-cell lymphoma, acute myeloid leukemia and multiple myeloma have more than half of their essential genes better predicted by transcripts.

Considering the whole transcriptome as the source for biomarkers, we studied the recurrence of each transcript biotype of the predicted biomarkers in comparison to the general biotypes (Figure 3). Ensembl [24] catalogs transcripts into four main biotypes: protein coding, pseudogene, long noncoding and short noncoding. These four main groups contain 35 subcategories in total. More than 90% of the transcriptome of the 412 cell lines taken together falls into 7 biotypes (out of 35), namely protein coding, nonsense mediated decay, lincRNA, miRNA, antisense, processed transcript and retained intron. Protein coding transcripts is the most represented category (around 40% of transcripts).

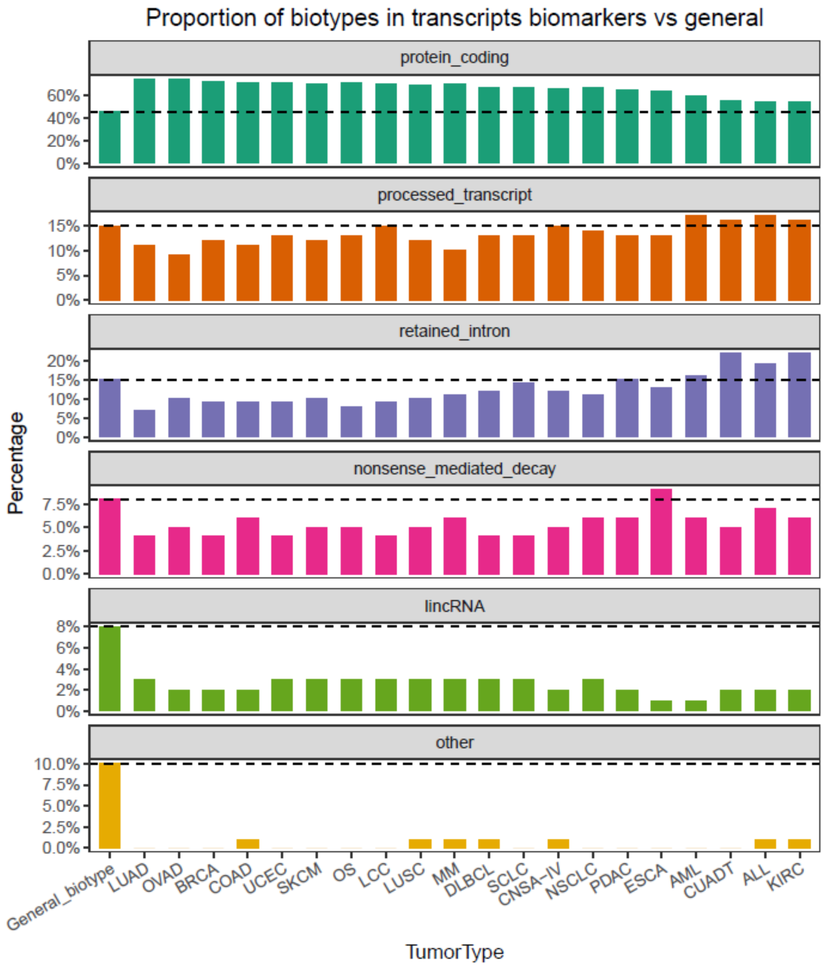

Figure 3. Proportion of transcript biotypes of biomarkers in 20 tumor types vs in general. Acronyms are included in Figure 2's caption. General_biotype shows the proportion of each specific biotype in the reference transcriptome (Gencode 24). Protein-coding transcripts are overrepresented as biomarkers for all tumor types.

We examined whether the biomarker's biotypes mimic the general distribution of biotypes in the transcriptome (Figure 3). Remarkably, five biotypes accounted for the vast majority of the biomarkers. Protein coding transcripts were the most abundant category across the 20 cell line

subtypes, and tended to be overrepresented when compared to the global proportion. miRNA and other small RNAs are underrepresented in the table. This result makes sense, since short RNAs are usually depleted before sequencing and thus, miRNA concentration cannot be properly measured. Intron retention is, with nonsense-mediated decay, the third most represented transcript biotype. The widespread abundance of intron retention in tumor transcriptome is well documented [25] but, to our knowledge, it has not been proposed as a possible source of biomarkers [26] or even neoantigens [27]. In fact, our results suggest that coding isoforms are better biomarkers. The roles of intron retention in cancer have yet to be elucidated. The primary fate of this class of AS is degradation through the nonsense-mediated mRNA decay mechanism (NMD). NMD results in reduced parent gene expression. However, it has been shown that certain intron retentions are capable of avoiding NMD and have been postulated to regulate the function of the parent gene in a dominant-negative manner [28].

Case study

To further illustrate the potential of this platform in precision medicine, we show a case study using renal carcinoma cell lines (n=14). We first conducted the gene essentiality analysis of these cell lines. We selected genes i) essential in at least 25% of renal cancer cell lines, setting the threshold for the DEMETER score as -2; ii) with a specificity odds ratio of at least 2; iii) with a minimum expression of 1TPM in at least 75% of cell lines when the genes are essential. Applying these parameters, 121 genes were found to be essential for renal carcinoma. Some of these genes belong to pathways known to be dysregulated in renal cancer (e.g., *ITGAV*, *TIAM1* and *PIK3CB*) [29]. Interestingly, 73 out of 121 genes (p-value = 1.1e-3, Fisher's exact test) have previously been identified as potential cancer drivers in other tumor types in mice according to the Candidate Cancer Gene Database [30].

Among these genes, PAX8 and HNF1B play a key role in renal carcinoma [31,32]. PAX proteins are transcription factors that regulate cell proliferation and migration of embryonic precursor cells [33]. The depletion of PAX2 by RNAi induces apoptosis in kidney carcinoma [34]. In addition, PAX2 and PAX8 double mutants cells do not exhibit mesenchymal–epithelial transition and in turn lack mesonephric tubules [35]. On the other hand, HNF1B is a transcription factor that acts as a tumor suppressor in renal carcinoma through control of PKHD1 expression [36].

Biomarkers for essential genes were obtained by running the *"Predict Biomarkers for a Target Gene"* panel. In this case, we focused on the interleukin-1 receptor-associated kinase (*IRAK*), which is implicated in cancer initiation and progression [37]. TranscriptAchilles revealed that all the proposed biomarkers for *IRAK1* (p-value < 1e-4, |log2FC| > 2 and local fdr < 0.1) were transcripts, which stresses the importance of splicing as a source of biomarkers.

The *HSP90AA1-005* transcript is one of the best markers of *IRAK1* essentiality (Figure 4). The *HSP90* gene play a role in the regulation of *IRAK1* [38]. Interestingly, while gene expression is not capable of distinguishing between sensitive and resistant groups of cell lines (p-value = 0.37; local fdr = 0.7; AUC = 0.63), the predicted transcript *HSP90AA1-005* is a good biomarker of *IRAK1*'s essentiality (p-value = 2.62e-07; local fdr = 0.01; AUC = 1).

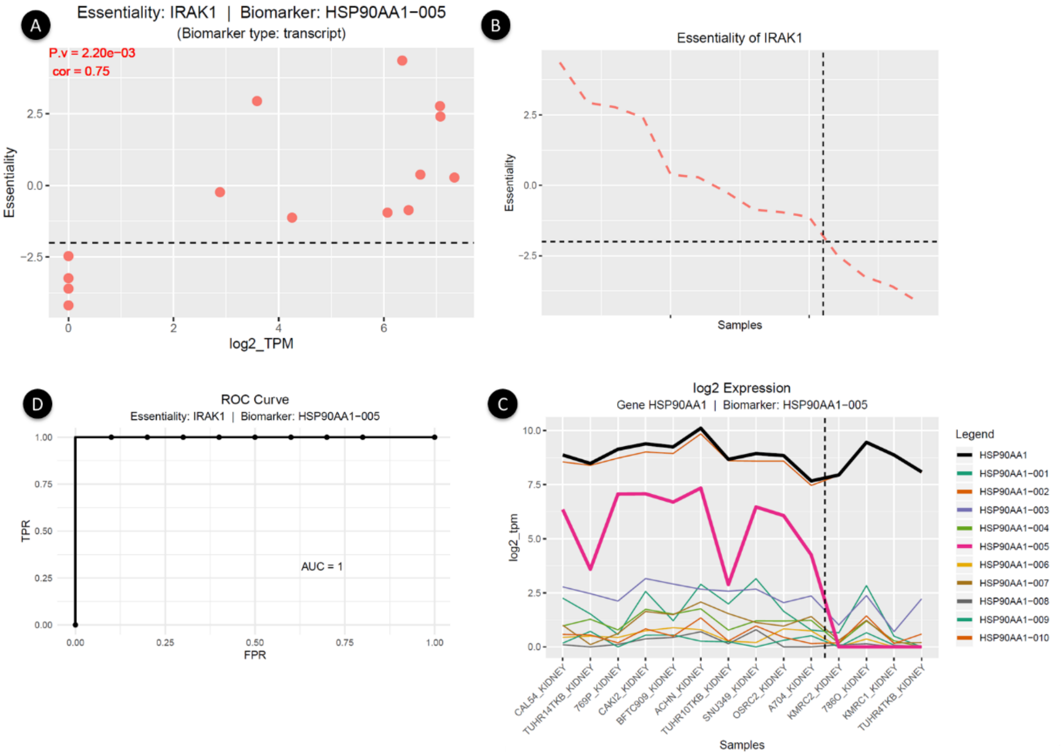

Figure 4. Output of TranscriptAchilles in renal carcinoma cell lines (n=14). HSP90AA1-005 is a transcript biomarker of essentiality of IRAK1. ***A)*** *Scatterplot of IRAK1 essentiality and HSP90AA1-005 log2-expression. Each dot represents a single cell line. The dotted black line marks the -2 essentiality threshold.* ***B)*** *Essentiality of IRAK1. Samples are sorted by its essentiality (more negative implies IRAK1 is more essential). Samples in panels B and C are sorted in the same order. The x-axes are shared by both panels. The black line marks the default essentiality score of -2 dividing the samples into resistant and sensitive to IRAK1 knock-down.* ***C)*** *log2-expression of gene HSP90AA1 (black line) and its transcripts. The dotted black line divides cell lines into resistant (left side) and sensitive (right side). The best biomarker (HSP90AA1-005) is shown in pink. In this case, transcript expression provides better essentiality markers than gene expression.* ***D)*** *ROC curve of the selected biomarker. Here the AUC is 1 but this is not generally the case.*

TranscriptAchilles can also predict genome-wide biomarkers for all essential genes and rank them according to their significance. We found companion biomarkers for 101 essential genes (out of 121). In 60% of cases, the best markers were transcripts rather than genes.

Figure 4, S7 and S6 show three essential gene and biomarker pairs (*IRAK1/ HSP90AA1-005*, *PER3/SEC31A-020*, *IRAK1/MAPK1-201*). In these cases, transcripts are differentially expressed between sensitive and resistant cell lines, whilst the corresponding genes do not show this pattern. In addition, over 95% of the proposed biomarkers for *IRAK1* and *PER3* were transcripts (p-value < 1e-4, |log2FC| > 2 and local fdr < 0.1).

The suggested essential gene-biomarker pairs are biologically sound. The interleukin-1 receptor-associated kinase (*IRAK*) plays a key role in the toll-like receptor (*TLR*) and interleukin-1 receptor (*IL1R*) signaling pathways, which are implicated in cancer initiation and progression [37]. Mitogen-activated protein kinase (*MAPK*) is involved in the regulation of normal cell proliferation, survival and differentiation. Aberrant regulation of *MAPK* contributes to cancer through the well-studied *Ras-Raf-MEK-ERK* pathway [39]. The relationship between *MAPK* and IRAK is also documented. IRAK participates in the activation of p38 *MAPK* by associating with *Ras* [40].

METHODS

Data sources and preprocessing

The Cancer Cell Line Encyclopedia (CCLE)(CCLE, RRID:SCR_013836) [41] provides public access to genomic data of near 900 cancer cell lines. The transcriptome profiles of these samples were calculated in a previous study [42] from raw RNA sequencing data using Kallisto (kallisto, RRID:SCR_016582) [43]. This study uses the Gencode 24 transcriptome (GRCh 38) as reference annotation [44]. This version of the transcriptome contains 199,169 transcripts. Transcript expression was measured in Transcripts Per Million (TPM) and filtered. In the filtering step, we excluded transcripts that had zero TPMs in every sample. Then, for the selected cohort of cell lines, we required the average expression of transcripts to be above a threshold, whose default value is 50% quantile of all the average expressions. After these filters, the resulting number of transcripts was around 90,000. This number depends on the selection of cell lines.

In the Achilles Project, 412 of these cell lines were interrogated for gene essentiality using shRNA. We used the DEMETER score as a measure of essentiality. DEMETER quantizes the competitive proliferation of the cell lines and minimizes the effect of off-target hybridizations by using a statistical model. The more negative the DEMETER score is, the more essential the gene is for a cell line. Authors of the DEMETER score established a cut-off of -2 as a threshold of essentiality. Genes with a DEMETER score lower than this threshold can be considered essentials for a cell line. Missing elements of DEMETER were imputed using the nearest neighbor averaging algorithm (KNN) [45].

Combining gene and isoform expression and DEMETER, we developed a statistical pipeline to find essential genes and predict the best markers of essentiality (Figure 5).

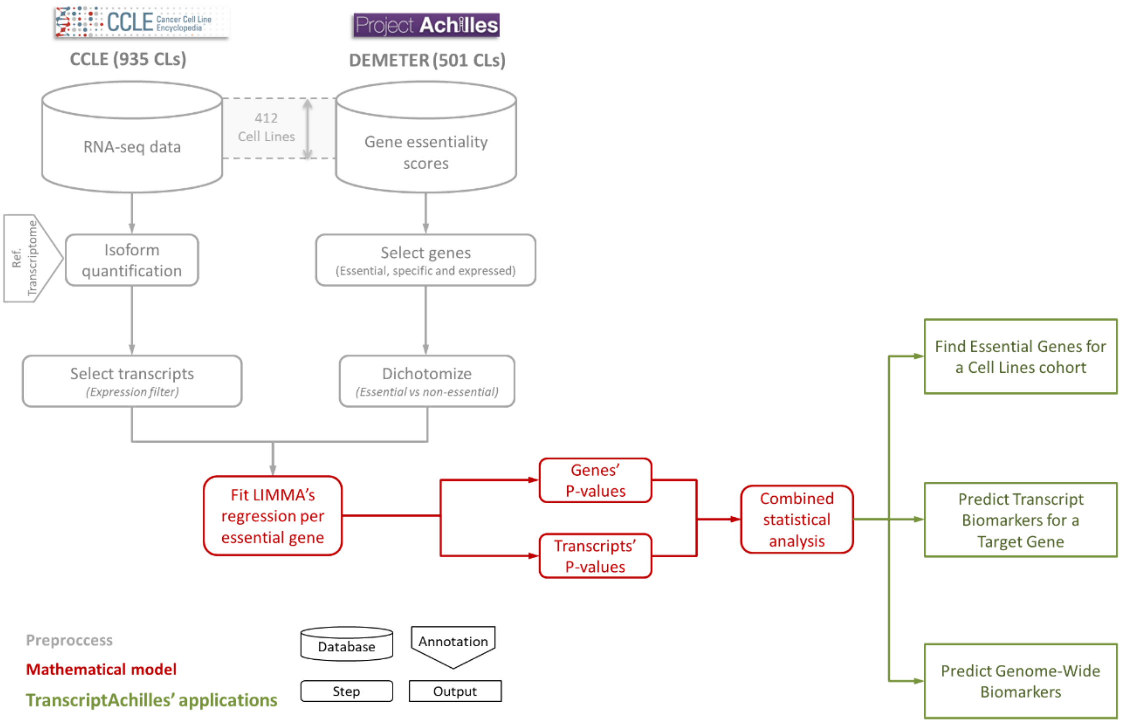

Figure 5. TranscriptAchilles' workflow. Database icons represent CCLE and Project Achilles data. 412 samples were matched between them. Step boxes represent algorithmic analysis, for both preprocessing (grey) and mathematical modeling (red). Green boxes represent applications of TranscriptAchilles.

Statistical model

Let e denote the number of RNAi target genes and n denote the number of screened samples. Let **D** be an $e \times n$ matrix of essentiality with each element d_{ij} representing the DEMETER score for the RNAi target *i* in sample *j*. Let **D*** be a $n \times e$ dichotomized matrix whose each element d^*_{ij} denotes whether sample *j* is resistant or sensitive to the RNAi target *i* as follows:

$$d^*_{ij} = \begin{cases} 1, & if\ d_{ij} < thr \quad (\text{Sensitive}\ ;\ S) \\ 0, & otherwise \quad (\text{Resistant}\ ;\ \text{R}) \end{cases},$$

where *thr* is a threshold whose default value is -2 as proposed in DEMETER.

Let ***s*** be a subset of *n' cell lines that* yields an essentiality vector $\boldsymbol{d}^*_{\boldsymbol{e_s}} = \left(d_{e_{s_1}}, \ldots, d_{e_{s_{n'}}}\right)$ for the eth RNAi target. Let $\boldsymbol{y}_{\boldsymbol{g_s}} = \left(y_{g_{s_1}}, \ldots, y_{g_{s_{n'}}}\right)$ be the expression vector of a putative gene biomarker and $\boldsymbol{y}_{\boldsymbol{t_s}} = \left(y_{t_{s_1}}, \ldots, y_{t_{s_{n'}}}\right)$ be an expression vector of one of their corresponding transcripts. The null hypotheses are defined as:

$$H_0^g:\ E\left(\boldsymbol{y}_{\boldsymbol{g_s}} \middle| \boldsymbol{d}^*_{\boldsymbol{e_s}} \in S\right) = E\left(\boldsymbol{y}_{\boldsymbol{g_s}} \middle| \boldsymbol{d}^*_{\boldsymbol{e_s}} \in R\right)$$

$$H_0^t:\ E\left(\boldsymbol{y}_{\boldsymbol{t_s}} \middle| \boldsymbol{d}^*_{\boldsymbol{e_s}} \in S\right) = E\left(\boldsymbol{y}_{\boldsymbol{t_s}} \middle| \boldsymbol{d}^*_{\boldsymbol{e_s}} \in R\right)$$

This null hypothesis is therefore: "the mean expression of a biomarker is identical in resistant and in sensitive cell lines to a gene knock-down". To test this hypothesis, we used a moderated t-test implemented in *limma* [18]. We applied this test for each RNAi target and all the expressed genes and transcripts to get the corresponding p-values. Dealing with these p-values implies solving two challenges: i) integrating transcripts and genes to get the best biomarkers and ii) correcting for multiple hypotheses.

In order to face these challenges, we followed a methodology similar to the IHW (Independent Hypothesis Weighting) procedure [46], which increases the power of a test by grouping the results using covariates. In our case, we divided the p-values corresponding to all the tests into 2n groups, where n is the number of knock-down genes (see Figure 6). Each group includes the p-values of either the transcripts or genes interrogating each knock-down gene.

For each of these groups, we computed the local false discovery rate (local fdr) [47]. The local fdr estimates, for each test, the probability of the null hypothesis to be true, conditioned on the observed p-values. The formula of the local fdr is the following:

$$P(H_0|z) = local\ fdr(z) = \frac{\pi_0 f_0(z)}{f(z)},$$

where z are the observed p-values, π_0 is the proportion of true null hypotheses –estimated from the data-, $f_0(z)$ the empirical null distribution –usually a uniform (0,1) distribution for well-designed tests- and $f(z)$ the mixture of the densities of the null and alternative hypothesis, also estimated from the data.

As stated in [47], "*the advantage of the local fdr is its specificity: it provides a measure of belief in gene i's 'significance' that depends on its p-value, not on its inclusion in a larger set of possible values*" as it occurs, for example with q-values or the standard False Discovery Rate. In addition, the clear statistical meaning of the local fdr (i.e., $P(H_0|z)$) allows for comparing genes with transcripts to provide the best biomarker taking into account that is a gene or a transcript. For example, in Figure 6, transcript are better biomarkers than genes for the first

knock-down gene and it occurs exactly the opposite for the last knock-down gene. Splitting the results into different groups increases the statistical power (as stated in [46]).

The local fdr and π_0 were estimated using the Bioconductor's R Package *qvalue* (Qvalue, RRID:SCR_001073)[48]. The value of π_0 gives an estimate on whether transcripts or genes are better biomarkers for a particular RNAi target, as observed in Figure 6. In addition, Figure S5 shows different real cases in which the best biomarkers are genes or isoforms.

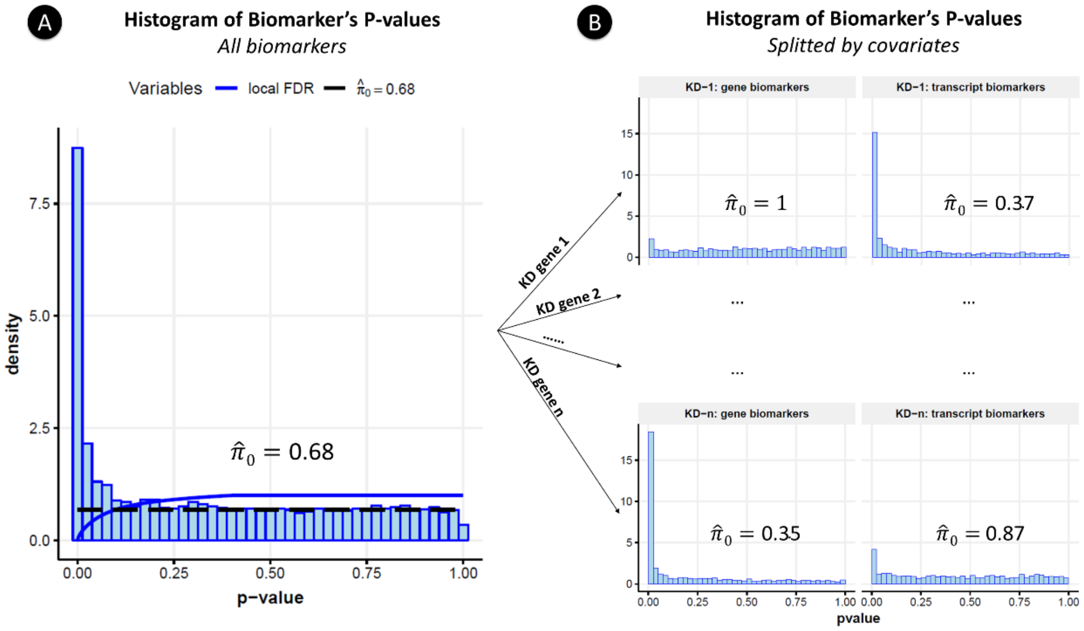

Figure 6. ***A)*** *Histogram of p-values of all tests (both genes and transcripts) taken together. The local false discovery rate (local FDR) and the $\hat{\pi}_0$ (the proportion of true null hypotheses) values are shown.* ***B)*** *Histogram of p-values after splitting by the covariates. The complete histogram in panel A gathers all the histograms in panel B. The covariates are, by rows: the knock-down (KD) genes; and, by columns: whether the biomarker is a gene or a transcript. In the KD gene 1, transcripts are better biomarkers than genes ($\hat{\pi}_0$=0.37 vs $\hat{\pi}_0$=1) and in the KD gene n occurs the opposite ($\hat{\pi}_0$=0.35 vs $\hat{\pi}_0$=1).*

DISCUSSION

We have developed TranscriptAchilles, a large-scale tool to predict genomic biomarkers associated with gene essentiality. This is the first approach that combines high-throughput RNA interference screenings with isoform expression. Besides, we have developed a methodology that combines gene and transcript expression to predict biomarkers of essentiality.

The two main technologies integrated in TranscriptAquilles are genome-wide loss-of-function RNA interference screens and whole-transcriptome expression profiling using RNA-seq. We will first discuss the potential and limitations of these technologies, and then, we will comment the results of TranscriptAchilles.

RNA interference screening provides an approach to predict genes that are essential for cell viability. Analyzing the output of these experiments is a challenge due to the off-target effects of shRNAs, which are mainly produced by the similarity of seed sequences. Several methodologies have explicitly modeled seed effects and dramatically improved the essentiality score [13–15,49]. In this scenario, the DEMETER score outperforms other summarization techniques. Despite the efforts made to decrease these errors, reducing the off-target effects

of shRNA remains a challenge when it comes to predicting the essentiality of the gene. In fact, DEMETER's developers are further improving their tool [16]. In addition, other promising loss-of-function approaches are emerging to identify essential genes, such as genome editing through the use of *CRISPR* [50].

The quantification of isoforms was done in a previous work using Kallisto. It could be argued that Kallisto only detects known isoforms included in a reference transcriptome, and that in cancer, there are many novel isoforms perhaps because of malfunctioning of the spliceosome [51]. Despite this disadvantage, isoform quantification algorithms –such as Kallisto- can be better adapted to compare disparate experiments. In addition, transcriptome annotation is ever increasing and improving filling gaps of previous versions. Kallisto was able to identify well-expressed isoforms that, in turn, were almost perfect biomarkers of the essentiality of their companion genes. Using other algorithms –such as Stringtie [52] or Cufflinks [53,54]- we could have discovered novel isoforms. Unfortunately, the specificity and sensitivity of the transcriptome reconstruction algorithms is well below 50% [55] and computation time is much larger. In summary, novel splicing events can be a fruitful source of biomarkers, but, given the present knowledge of the transcriptome, known isoforms also present great potential as a source of biomarkers in precision medicine and are much easier to integrate.

Regarding TranscriptAchilles, we will discuss the three steps of the pipeline: i) selecting the cohort of cell lines, ii) finding essential genes and iii) predicting biomarkers. The standard usage of the pipeline begins by selecting a single tumor subtype. The user can also choose a combination of tumors according to other characteristics such as histology (e.g. lung and stomach adenocarcinoma). Within this cohort, the algorithm finds genes that are essential for cell viability. Essential genes are also required to be specific for the selected cohort (when compared with the rest of the cell lines). Setting this parameter is important to exclude genes that are essential for any cell and could be a source of strong side-effects in a potential therapy.

The algorithm also predicts the best biomarkers (either genes or transcripts) of gene essentiality. We filtered the transcripts according to their expression before running the statistical model since more than 30% are not expressed at all in our dataset. Our model integrates genes and transcripts and, with the aid of their corresponding local fdr, selects (if existing) the proper biomarker for each cancer target.

The analysis throughout 20 tumor subtypes suggested that the incorporation of splicing complements gene expression to find biomarkers in several cancer types. This is the case of skin carcinoma, esophagus squamous carcinoma, lung large cell carcinoma and multiple myeloma among others. In other tumors –such as lung adenocarcinoma, acute lymphocyte leukemia and colon adenocarcinoma- an analysis based merely on gene expression recalled more than 60% of the biomarkers. Unsurprisingly, the proportion of coding transcripts in the predicted biomarkers is higher than what is expected by chance in almost all cancer subtypes.

Finally, we showed a case study of the pipeline using kidney carcinoma cell lines. This example can easily be replicated using the application. In kidney carcinoma, 60% of essential genes were better marked by transcripts rather than by genes. Based on this study, the inhibition of IRAK1 is proposed as a new potential therapeutic strategy in this tumor.

TranscriptAchilles opens a wide range of translational applications in cancer, especially in those cases that lack an effective therapy or an adequate response biomarker. Future work

may exploit this powerful technique in combination with mutations, copy number variations or chromatin modifications to find new potential drug targets with their corresponding biomarkers.

ABBREVIATIONS

AS: Alternative Splicing; CCLE: Cancer Cell Line Encyclopedia; FDR: False Discovery Rate; NMD: nonsense-mediated mRNA decay; TCGA: The Cancer Genome Atlas; TPM: Transcripts Per Million

Availability of supporting source code and requirements

- Project name: TranscriptAchilles
- Project home page: https://gitlab.com/fcarazo.m/transcriptachilles
  http://biotecnun.unav.es:8080/app/TranscriptAchilles
- Operating systems: Platform independent
- Programming language: R
- Other requirements: RShiny, CRAN
- License: GNU GPL v3
- RRID:SCR_016849)

AVAILABILITY OF SUPPORTING DATA

Snapshots of the code are available in the *GigaScience* GigaDB repository[57].

FUNDING

Research reported in this publication was supported by the Provincial Council of Gipuzkoa through the MINEDRUG project: "Predicting therapy response in oncology using Big Data analysis" and the Basque Government with the grant promoting doctoral theses for young pre-doctoral researchers [grant numbers: PRE_2017_2_0033 to FC and PRE_2017_1_0327 to XC].

ACKNOWLEDGMENTS

The authors are grateful to Fernando Carazo-Villalaín for his technical support on web server hosting and to María J. López for her fruitful comments on the preparation of this manuscript.

REFERENCES

1. Nilsen TW, Graveley BR. Expansion of the eukaryotic proteome by alternative splicing. Nature. 2010;463:457–63.
2. Park E, Pan Z, Zhang Z, Lin L, Xing Y. The expanding landscape of alternative splicing variation in human populations. Am J Hum Genet. The Authors; 2018;102:11–26. doi:10.1016/j.ajhg.2017.11.002
3. Sebestyén E, Singh B, Miñana B, Pagès A, Mateo F, Pujana MA, et al. Large-scale analysis of genome and transcriptome alterations in multiple tumors unveils novel cancer-relevant splicing networks. Genome Res. 2016;26:732–44.
4. Sveen A, Kilpinen S, Ruusulehto A, Lothe R a, Skotheim RI. Aberrant RNA splicing in cancer; expression changes and driver mutations of splicing factor genes. Oncogene. Nature Publishing Group; 2015;35:1–15. Available from: doi:10.1038/onc.2015.318%5Cn
5. Ladomery M. Aberrant alternative splicing is another hallmark of cancer. Int J Cell Biol. Hindawi Publishing Corporation; 2013;2013.

6. Oltean S, Bates DO. Hallmarks of alternative splicing in cancer. Oncogene. Nature Publishing Group; 2014;33:5311–8. doi:10.1038/onc.2013.533
7. Garcia-Blanco MA, Baraniak AP, Lasda EL. Alternative splicing in disease and therapy. Nat Biotechnol. 2004;22:535–46.
8. Safikhani Z, Smirnov P, Thu KL, Silvester J, El-Hachem N, Quevedo R, et al. Gene isoforms as expression-based biomarkers predictive of drug response in vitro. Nat Commun. 2017;8.
9. Carazo F, Romero JP, Rubio Á. Upstream analysis of alternative splicing: a review of computational approaches to predict context-dependent splicing factors. Brief Bioinform. 2018;50.
10. Vitting-Seerup K, Sandelin A. The Landscape of Isoform Switches in Human Cancers. Mol Cancer Res. 2017;15:1206–21. doi:10.1158/1541-7786.MCR-16-0459
11. Climente-Gonzalez H, Porta-Pardo E, Godzik A, Eyras E. The Functional Impact of Alternative Splicing in Cancer. 2017;2215–26.
12. Cowley GS, Weir BA, Vazquez F, Tamayo P, Scott J, Rusin S, et al. Parallel genome-scale loss of function screens in 216 cancer cell lines for the identification of context-specific genetic dependencies. Sci data. 2014;1:140035.
13. Tsherniak A, Vazquez F, Montgomery PG, Weir BA, Kryukov G, Cowley GS, et al. Defining a Cancer Dependency Map. Cell 2017;170:564–576.e16. Aoi:10.1016/j.cell.2017.06.010
14. Shao DD, Tsherniak A, Gopal S, Weir BA, Tamayo P, Stransky N, et al. ATARiS: Computational quantification of gene suppression phenotypes from multisample RNAi screens. Genome Res. 2013;23:665–78.
15. Hart T, Brown KR, Sircoulomb F, Rottapel R, Moffat J. Measuring error rates in genomic perturbation screens: gold standards for human functional genomics. Mol Syst Biol. 2014;10:733–733. doi:10.15252/msb.20145216
16. Mcfarland JM, Ho Z V, Kugener G, Dempster JM, Montgomery PG, Bryan JG, et al. Improved estimation of cancer dependencies from large-scale RNAi screens using model- based normalization and data integration. bioRxiv. 2018;305656.
https://www.biorxiv.org/content/early/2018/04/24/305656.abstract%0Apapers3://publication/doi/10.1101/305656
17. Aguirre AJ, Meyers RM, Weir BA, Vazquez F, Zhang CZ, Ben-David U, et al. Genomic copy number dictates a gene-independent cell response to CRISPR/Cas9 targeting. Cancer Discov. 2016;6:914–29.
18. Ritchie ME, Phipson B, Wu D, Hu Y, Law CW, Shi W, et al. Limma powers differential expression analyses for RNA-sequencing and microarray studies. Nucleic Acids Res. 2015;43:e47.
19. Weinstein IB, Joe A. Oncogene addiction. Cancer Res. 2008;68:3077–80.
20. Toledo F, Wahl GM. MDM2 and MDM4: p53 regulators as targets in anticancer therapy. Int J Biochem Cell Biol. 2007;39:1476–82.
21. R Development Core Team. R: a language and environment for statistical computing. [http://wwwRproject.org]. 2003.
22. Chang W, Cheng J, Allaire J, Xie Y, McPherson J. shiny: Web application framework for R. [http://CRAN R-project org/package= shiny]. 2017;
23. Verbeke T, Michielssen F. ShinyProxy--open source enterprise deployment for shiny. GitHub Repos. 2016;
24. Zerbino DR, Achuthan P, Akanni W, Amode MR, Barrell D, Bhai J, et al. Ensembl 2018. Nucleic Acids Res. 2018;46:D754–61.
25. Dvinge H, Bradley RK. Widespread intron retention diversifies most cancer transcriptomes. Genome Med [Internet]. Genome Medicine; 2015;7:1–13. doi:10.1186/s13073-015-0168-9
26. Xi X, Li T, Huang Y, Sun J, Zhu Y, Yang Y, et al. RNA Biomarkers: Frontier of Precision Medicine for Cancer. Non-Coding RNA. 2017;3:9. Available from: http://www.mdpi.com/2311-553X/3/1/9
27. Smart AC, Margolis CA, Pimentel H, He MX, Miao D, Adeegbe D, et al. Intron retention as a novel source of cancer neoantigens. bioRxiv. 2018;309450. https://www.biorxiv.org/content/early/2018/04/27/309450
28. Braunschweig U, Barbosa-Morais NL, Pan Q, Nachman EN, Alipanahi B, Gonatopoulos-Pournatzis T, et al. Widespread intron retention in mammals functionally tunes transcriptomes. Genome Res. 2014;24:1774–86.
29. Liu X, Wang J, Sun G. Identification of key genes and pathways in renal cell carcinoma through expression profiling data. Kidney Blood Press Res. 2015;40:288–97.
30. Abbott KL, Nyre ET, Abrahante J, Ho YY, Vogel RI, Starr TK. The candidate cancer gene database: A database of cancer driver genes from forward genetic screens in mice. Nucleic Acids Res. 2015;43:D844–8.
31. Clissold RL, Hamilton AJ, Hattersley AT, Ellard S, Bingham C. HNF1B-associated renal and extra-renal disease—an expanding clinical spectrum. Nat Rev Nephrol. 2014;11:102–12. doi:10.1038/nrneph.2014.232
32. Chang A, Brimo F, Montgomery EA, Epstein JI. Use of PAX8 and GATA3 in diagnosing sarcomatoid renal cell carcinoma and sarcomatoid urothelial carcinoma. Hum Pathol. Elsevier Inc.; 2013;44:1563–8. doi:10.1016/j.humpath.2012.12.012
33. Robson EJD, He SJ, Eccles MR. A PANorama of PAX genes in cancer and development. Nat Rev Cancer. 2006;6:52–62.
34. Dressler GR, Wilkinson JE, Rothenpieler UW, Patterson LT, Williams-Simons L, Westphal H. Deregulation of Pax-2 expression in transgenic mice generates severe kidney abnormalities. Nature. Nature Publishing Group; 1993;362:65.
35. Bouchard M, Souabni A, Mandler M, Neubüser A, Busslinger M. Nephric lineage specification by Pax2 and Pax8. Genes Dev. 2002;16:2958–70.

36. Rebouissou S, Vasiliu V, Thomas C, Bellanné-Chantelot C, Bui H, Chrétien Y, et al. Germline hepatocyte nuclear factor 1α and 1β mutations in renal cell carcinomas. Hum Mol Genet. 2005;14:603–14.
37. Rhyasen GW, Starczynowski DT. IRAK signalling in cancer. Br J Cancer. Nature Publishing Group; 2015;112:232–7. doi:10.1038/bjc.2014.513
38. De Nardo D, Masendycz P, Ho S, Cross M, Fleetwood AJ, Reynolds EC, et al. A central role for the Hsp90·Cdc37 molecular chaperone module in interleukin-1 receptor-associated-kinase-dependent signaling by Toll-like receptors. J Biol Chem. 2005;280:9813–22.
39. Roberts PJ, Der CJ. Targeting the Raf-MEK-ERK mitogen-activated protein kinase cascade for the treatment of cancer. Oncogene. 2007;26:3291–310.
40. McDermott EP, O'Neill LAJ. Ras participates in the activation of p38 MAPK by interleukin-1 by associating with IRAK, IRAK2, TRAF6, and TAK-1. J Biol Chem. 2002;277:7808–15.
41. Barretina J, Caponigro G, Stransky N, Venkatesan K, Margolin A a, Kim S, et al. The Cancer Cell Line Encyclopedia enables predictive modelling of anticancer drug sensitivity Supp. Nature. 2012;483:603–7.
42. Tatlow PJ, Piccolo SR. A cloud-based workflow to quantify transcript-expression levels in public cancer compendia. Sci Rep. 2016;6:39259.
43. Bray NL, Pimentel H, Melsted P, Pachter L. Near-optimal probabilistic RNA-seq quantification. Nat Biotechnol [Internet]. 2016;34:525–7. doi:10.1038/nbt.3519
44. Harrow J, Frankish A, Gonzalez JM, Tapanari E, Diekhans M, Kokocinski F. GENCODE: The Reference Human Genome Annotation for The ENCODE Project. Genome Res. 2012;22:1760–74. doi:10.1101/gr.135350.111
45. Brown P, Hastie T, Tibshirani R, Botstein D, Altman RB. Missing value estimation methods for DNA microarrays. Bioinformatics. 2001;17:520–5.
46. Ignatiadis N, Klaus B, Zaugg JB, Huber W. Data-driven hypothesis weighting increases detection power in genome-scale multiple testing. Nat Methods. 2016;13:577–80.
47. Efron B, Efron B, Tibshirani R, Tibshirani R. Empirical bayes method and false discovery rates for microarrays. Genet Epidemiol. 2002;23:70–86.
48. Storey JD. A direct approach to false discovery rates. J R Stat Soc Ser B Stat Methodol. 2002;64:479–98.
49. Jaiswal A, Peddinti G, Akimov Y, Wennerberg K, Kuznetsov S, Tang J, et al. Seed-effect modeling improves the consistency of genome-wide loss-of-function screens and identifies synthetic lethal vulnerabilities in cancer cells. Genome Medicine; 2017;9:51. doi:10.1186/s13073-017-0440-2
50. Meyers RM, Bryan JG, McFarland JM, Weir BA, Sizemore AE, Xu H, et al. Computational correction of copy number effect improves specificity of CRISPR-Cas9 essentiality screens in cancer cells. Nat Genet. 2017;49:1779–84.
51. Ritchie W, Granjeaud S, Puthier D, Gautheret D. Entropy measures quantify global splicing disorders in cancer. PLoS Comput Biol. 2008;4:1–9.
52. Pertea M, Pertea GM, Antonescu CM, Chang T-C, Mendell JT, Salzberg SL. StringTie enables improved reconstruction of a transcriptome from RNA-seq reads. Nat Biotechnol. 2015;33:290–5.
53. Trapnell C, Williams BA, Pertea G, Mortazavi A, Kwan G, van Baren MJ, et al. Transcript assembly and quantification by RNA-Seq reveals unannotated transcripts and isoform switching during cell differentiation. Nat Biotechnol. Nature Publishing Group; 2010;28:511–5. doi:10.1038/nbt.1621
54. Trapnell C, Hendrickson DG, Sauvageau M, Goff L, Rinn JL, Pachter L. Differential analysis of gene regulation at transcript resolution with RNA-seq. Nat Biotechnol. Nature Publishing Group; 2013;31:46–53.
55. Steijger T, Abril JF, Engström PG, Kokocinski F, Hubbard TJ, Guigó R, et al. Assessment of transcript reconstruction methods for RNA-seq. Nat Methods. Nature Publishing Group; 2013;10:1177–84.
56. Mazzoccoli G, Piepoli A, Carella M, Panza A, Pazienza V, Benegiamo G, et al. Altered expression of the clock gene machinery in kidney cancer patients. Biomed Pharmacother [Internet]. Elsevier Masson SAS; 2012;66:175–9. Available from: http://dx.doi.org/10.1016/j.biopha.2011.11.007
57. Carazo F; Campuzano L; Cendoya X; Planes FJ; Rubio A (2019): Supporting data for "TranscriptAchilles: a genome-wide platform to predict isoform biomarkers of gene essentiality in cancer" GigaScience Database. http://dx.doi.org/10.5524/100563

Supplementary Material

TranscriptAchilles: a genome-wide platform to predict transcript biomarkers and drug target genes in cancer

Fernando Carazo[1], Lucía Campuzano[2], Xabier Cendoya[1], Francisco J. Planes[1] and Angel Rubio[1]*

1 Tecnun (University of Navarra), Paseo Manuel Lardizábal 15, 20018 San Sebastián, SPAIN

2 University of Luxembourg, 2, avenue de l'Université, 4365 Esch-sur-Alzette, LUXEMBOURG

** Corresponding author: Angel Rubio, e-mail: arubio@tecnun.es*

SECTION 1. Quick start

The page of the web-app contains an *Overview* panel with detailed information about the tool and the pipeline for performing an analysis with the app. In the *Help* panel, further details of the different features can be found. The functionalities of TranscriptAchilles are presented in a set of panels in the app. Figure S1 shows the pipeline of TranscriptAchilles.

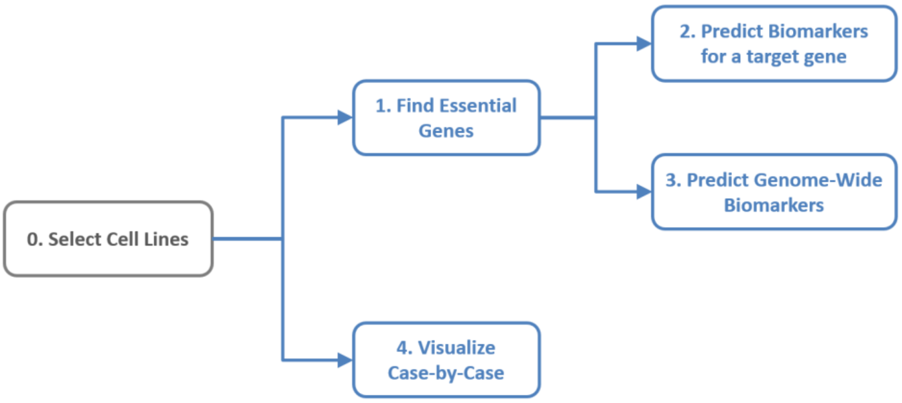

Figure S1. Quick start: pipeline

The main panels of the platform are:

0. Select cell lines. The user can select the cohort of cell lines to be analyzed. Several primary sites and subtypes can be selected at the same time. The application is pre-loaded with all the necessary data, so that the user does not need to upload any file.

1. Find Essential Genes. TranscriptAchilles identifies putative drug targets for the selected cell lines. Essential genes are required to meet several criteria: 1) they must be essential for a minimum percentage of samples in the selected subtype, 2) they must be specific for the subtype under study and 3) they must be expressed. In order to achieve these three requirements, the user must set several thresholds. The first one is the percentage of cell lines that are sensitive to the gene knockdown of interest. The second one is an odds ratio, which can be illustrated with an example: if the enrichment is set to 2, the percentage of cell lines sensitive to the gene knockdown must be two times larger for the cell lines under study than for the rest of cell lines in the DEMETER dataset. Finally, a threshold on minimum TPM (transcripts per million) expression can be set to ensure that the gene is expressed.

2. Predict Biomarkers for a Target Gene. In this section, the user can select one or more genes from the previous step and predict putative biomarkers of their essentiality. The statistical model estimates the local false discovery rate for both genes and transcripts and decides whether genes or transcripts are the best markers for each case (see Methods section of the main manuscript).

3. Predict Genome-Wide Biomarkers. In this case the biomarkers are found for all the essential genes identified in the step *Find Essential Genes.*

4. Visualize Case-by-Case. The user can visualize the essentiality of any gene and transcript biomarker. This panel can be run once the cell lines are selected.

0. Select cell lines

The user is required to select the cohort of cell lines to be analyzed. Several primary sites and subtypes can be selected at the same time. The application is pre-loaded with all the necessary data, so that the user does not need to upload any data.

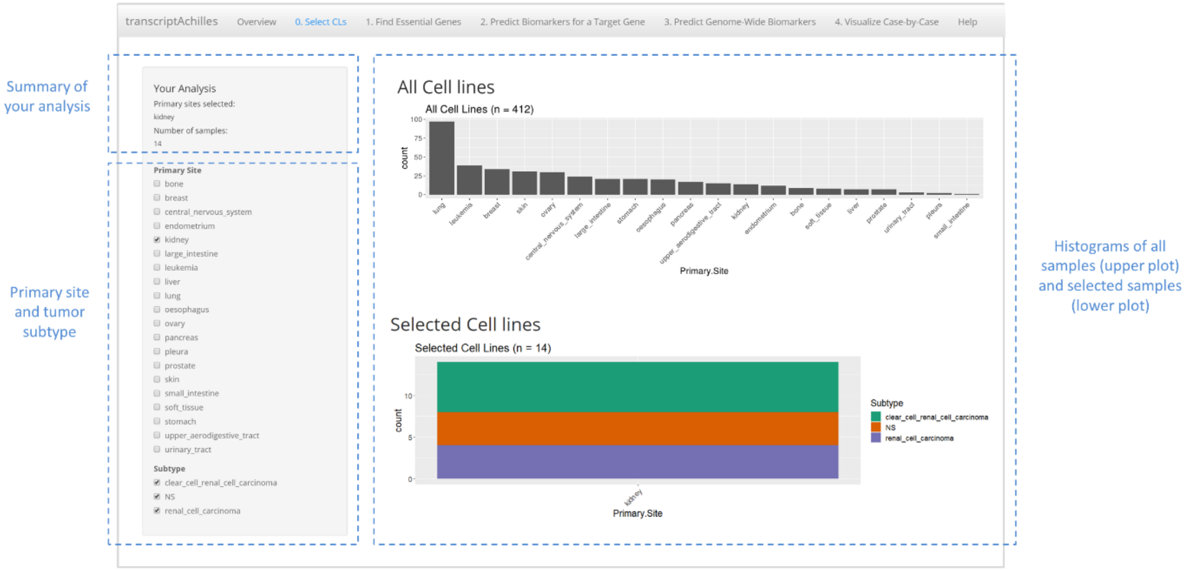

Figure S2. Quick start: selection of samples

1. Find essential genes

The second step of the analysis consists in extracting the essential gene list for the selected cell lines. Five tunable parameters allow the user to tailor the output. The toolbox provides a default value for each parameter. The assigned values correspond to our understanding of the minimum conditions which need to be satisfied by a gene to be essential.

The filters correspond to three criteria: essentiality, specificity and expression. Essentiality is a two-legged characteristic. It refers to the percentage of selected cell lines that have a DEMETER score lower than the essentiality cut-off. Specificity is represented by the enrichment ratio. This filter allows the user to define the minimum ratio between the proportion of selected cell lines for which a gene is essential and the proportion of the rest of the cell lines for which the same gene is essential.

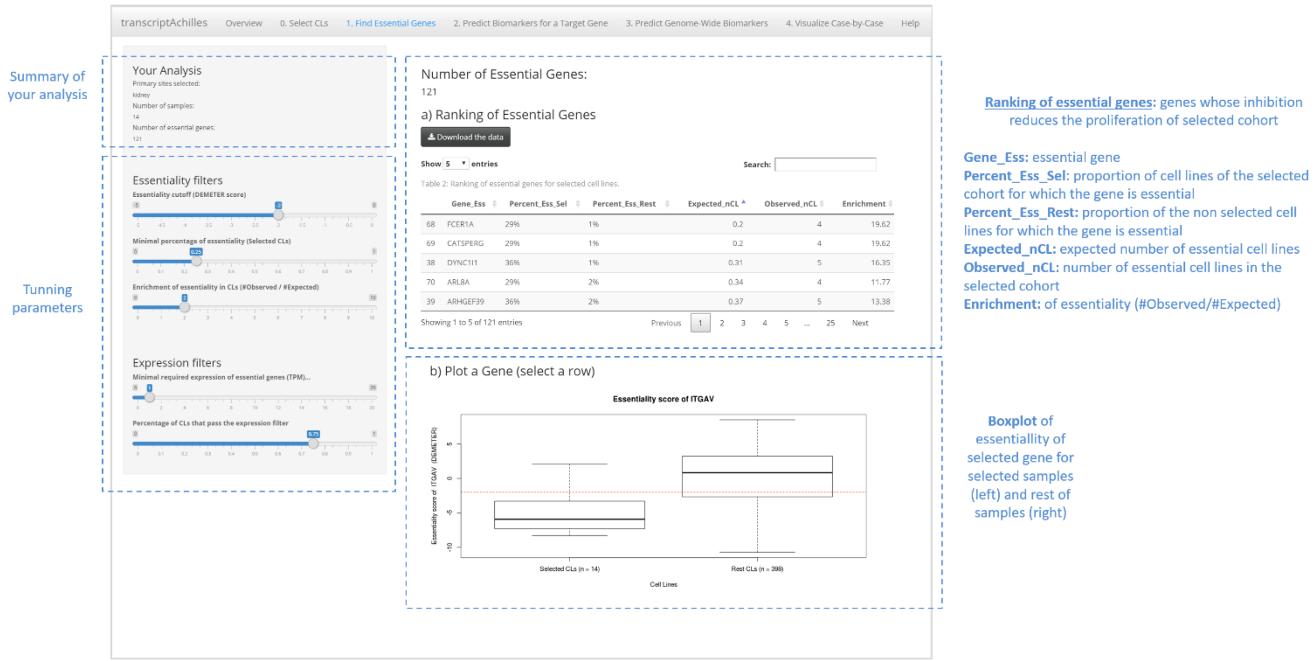

Figure S3. Quick start: essential genes

2. Predict biomarkers for a target gene

In this section, the user can select one or more genes of the previous step and predict putative biomarkers for their essentiality. In each case, the application decides whether genes or transcripts are the best markers.

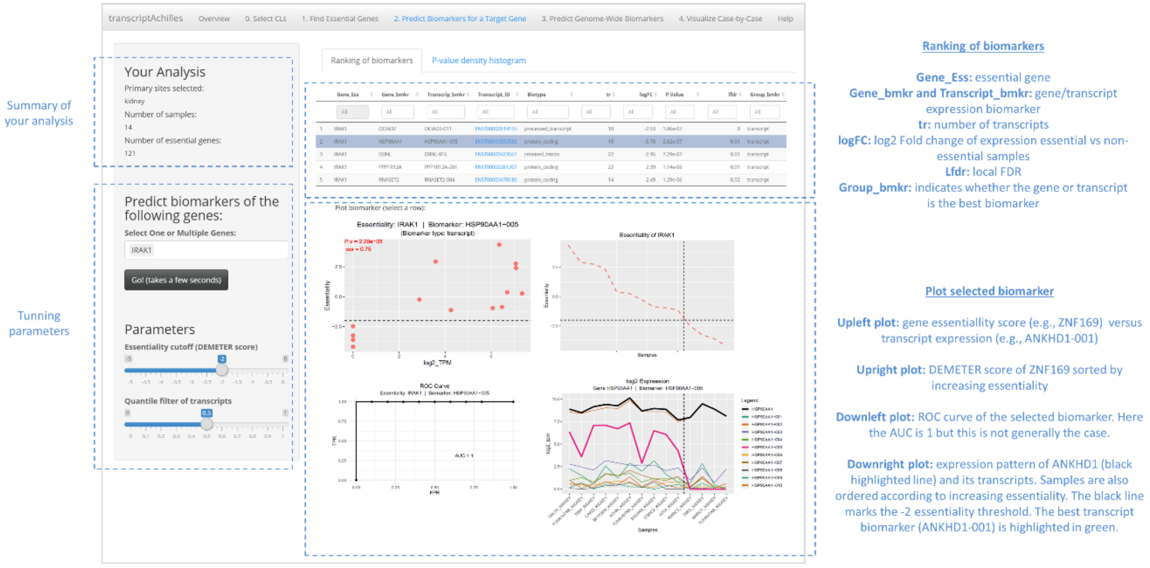

Figure S4. Quick start: prediction of transcript biomarkers

3. Predict Genome-Wide Biomarkers.

In this case the biomarkers are found for all the essential genes identified in the step *Find Essential Genes.*

4. Visualize Case-by-Case.

The user can also visualize the essentiality of any gene and transcript biomarker. This panel can be run once the cell lines are selected

SECTION 2. Other examples of TranscriptAchilles

Three examples of TranscriptAchilles in kidney carcinoma:

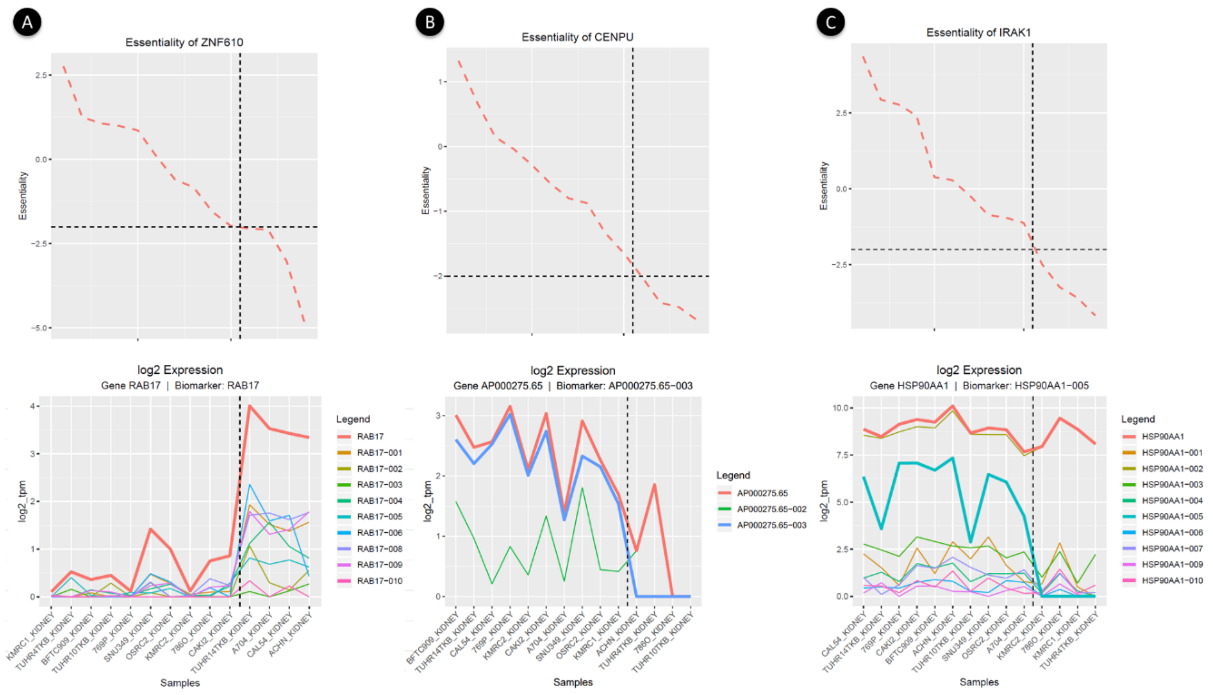

Figure S5. Three examples of TranscriptAchilles in kidney carcinoma (n = 14). In each example, the essentiality of a gene for every cell line and the log2 expression values of the gene biomarker are shown in the upper and lower plot, respectively. The cell lines are ordered according to increasing essentiality. The vertical dotted line separates the cell lines into resistant (left) and sensitive (right) to the inhibition of the essential gene (DEMETER score < -2). Gene expression is highlighted in red. The best transcript biomarker is also highlighted. When the best biomarker is the gene, no transcript is highlighted. ***A)*** *Essentiality of ZNF610. The biomarker is the gene expression of RAB17.* ***B)*** *Essentiality of CENPU. The best biomarker is isoform AP000275.65-003.* ***C)*** *Essentiality of IRAK1. The isoform biomarker is not the most expressed isoform. Gene expression is not a good biomarker. However, there is a clear expression change in Isoform HSP90AA1-005.*

Once essential genes are identified the tool allows the prediction of companion biomarkers, as none of the putative target genes is essential for 100% of renal cell lines. These biomarkers (gene or transcript) are obtained by running the Predict Genome-Wide Biomarkers section of the tool.

PER3 belongs to the period circadian regulator family, which is reported to be dysregulated in kidney tumors [56]. SEC31A is expressed by all renal cell lines, but its transcript SEC31A-020 is absent in sensitive cell lines.

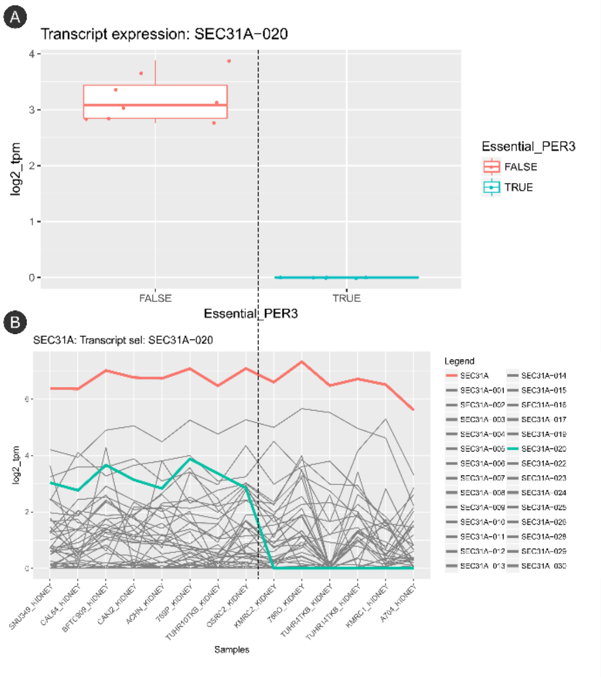

Figure S6. A) log2-expression boxplot of the predicted transcript biomarker (SEC31A-020) in renal cancer cell lines (n = 14). PER3 sensitive (red) and resistant (blue) cell lines are shown. B) Expression pattern of gene SEC31A (red highlighted line) and its transcripts. Samples are ordered according to increasing essentiality. The black line marks the -2 essentiality threshold. The best transcript biomarker (SEC31A-020) is highlighted in blue.

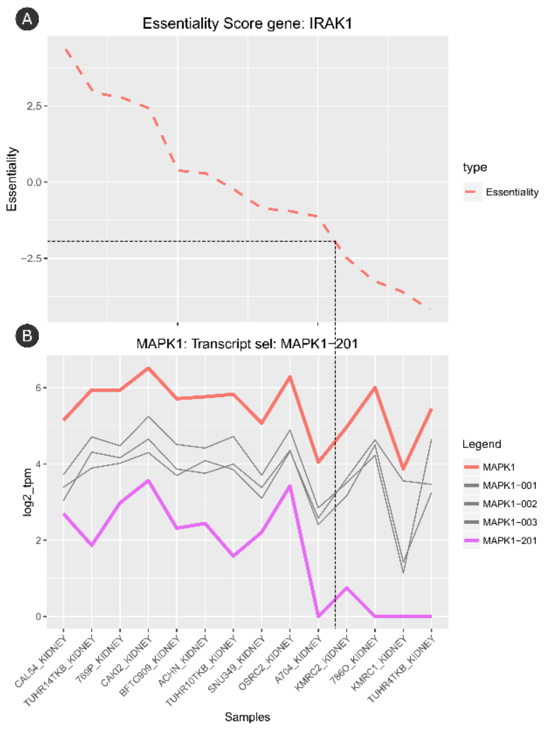

Figure S7. Predicted target gene (IRAK1) in renal carcinoma cell lines (n = 14) with its companion biomarker (transcript MAPK1-201). A) renal cell lines ordered by increasing essentiality of IRAK1.. The dotted black line marks the default essentiality score of -2. B) Expression pattern of gene MAPK1 (red highlighted line) and its transcripts. Samples are ordered according to increasing essentiality of IRAK1. The dotted black line marks the -2 essentiality threshold dividing cell lines into resistant (left side) and sensitive (right side). The best transcript biomarker (MAPK1-201) is highlighted in purple. In this case, transcript expression is a better marker of essentiality than gene expression.

SECTION 3. Positive controls of DEMETER

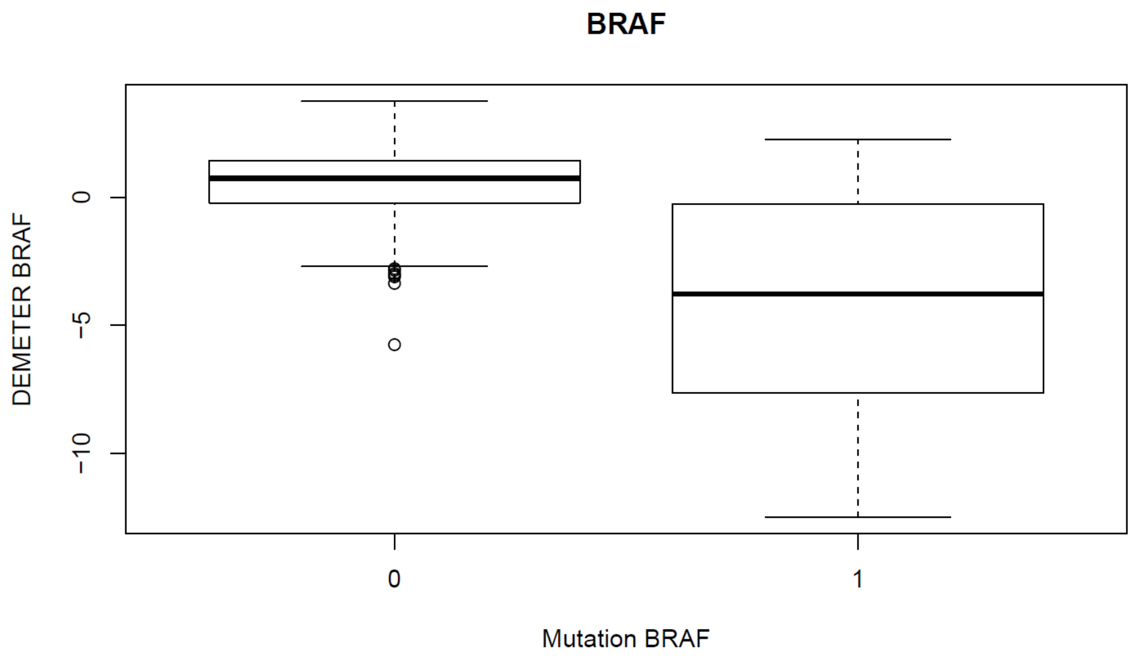

Figure S8. BRAF oncogene. Essentiality of BRAF for BRAF wt (0) and BRAF mut (1) in 412 samples.

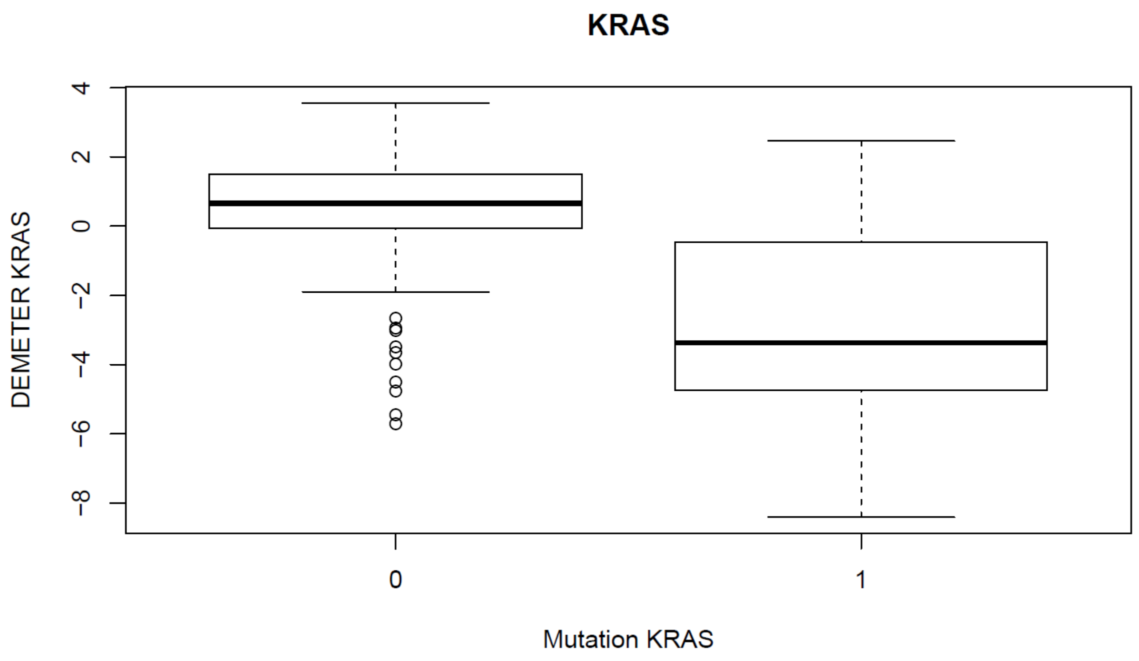

Figure S9. KRAS oncogene. Essentiality of KRAS for KRAF wt (0) and KRAS mut (1) in 412 samples.

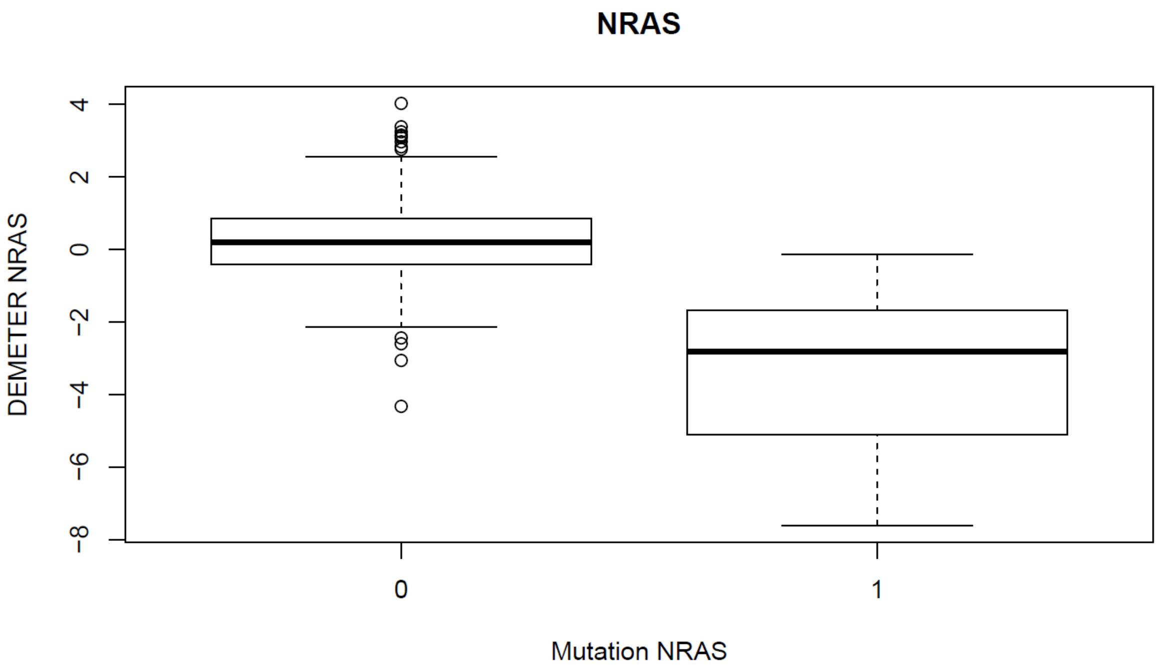

Figure S10. NRAS oncogene. Essentiality of NRAS for NRAS wt (0) and NRAS mut (1) in 412 samples.

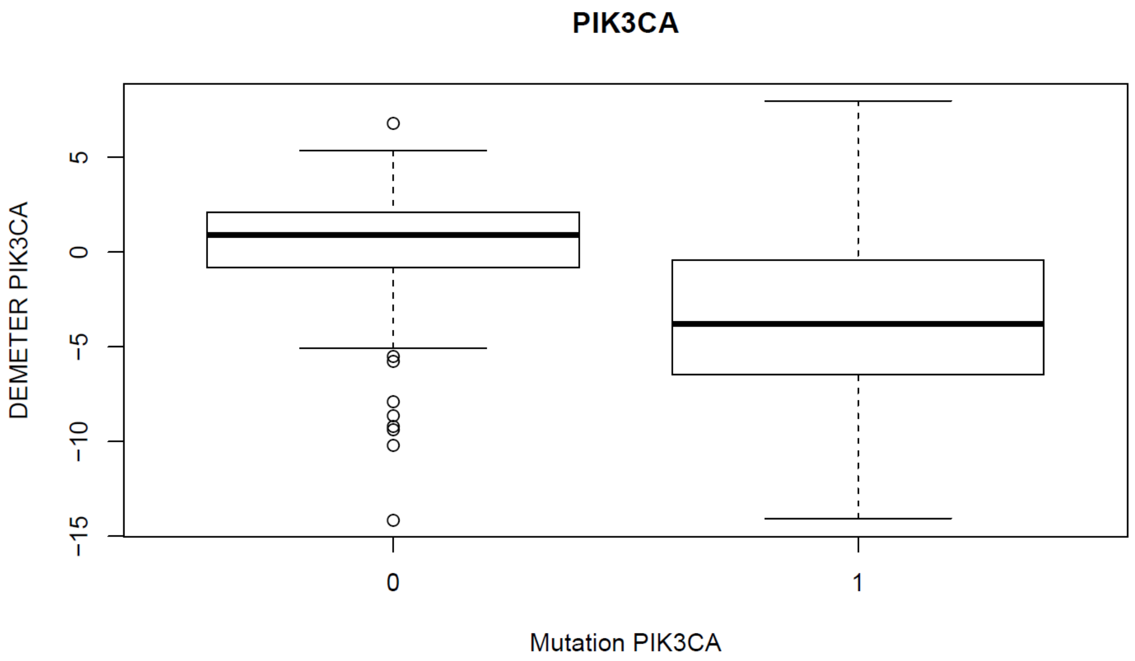

Figure S11. PIK3CA oncogene. Essentiality of PIK3CA for PIK3CA wt (0) and PIK3CA mut (1) in 412 samples.

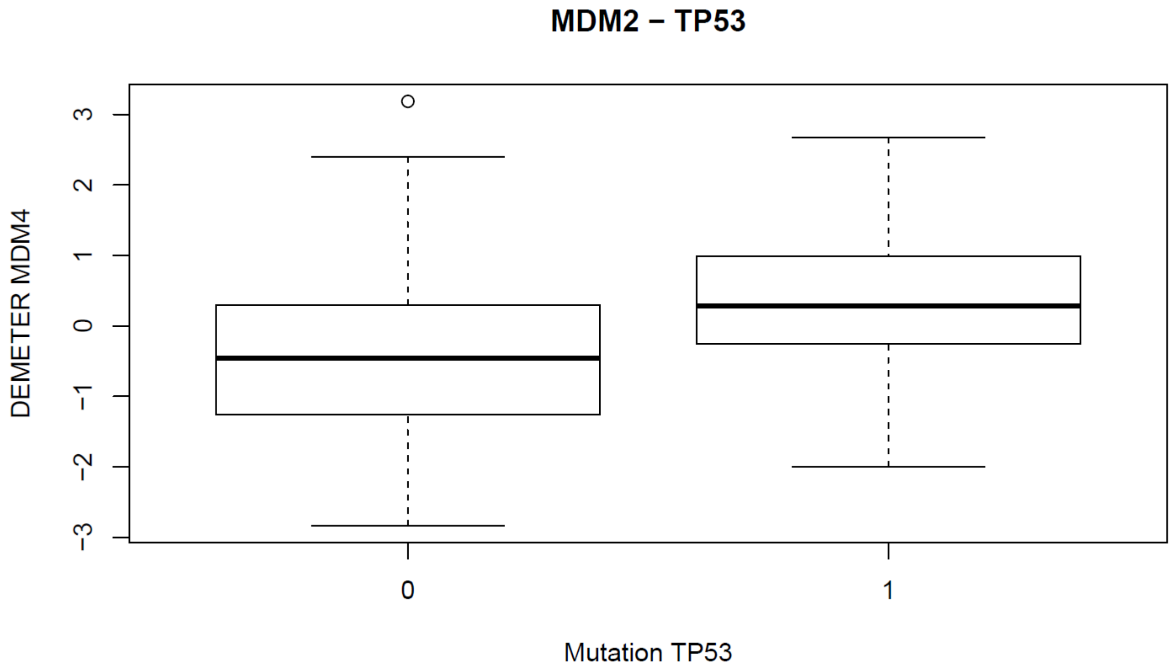

Figure S12. TP53 mutation and MDM2. Essentiality of MDM2 for TP53 wt (0) and MDM2 mut (1) in 412 samples. MDM2 is known to be essential if TP53 is functional -TP53 wt (0).

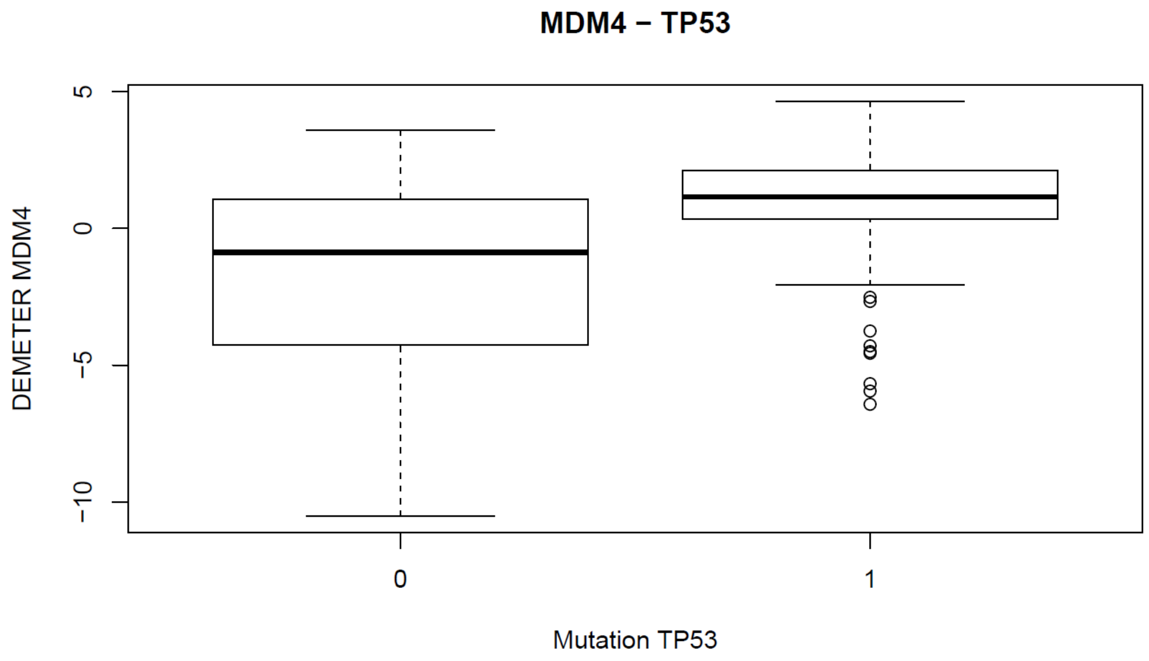

Figure S13. TP53 mutation and MDM4. Essentiality of MDM4 for TP53 wt (0) and MDM4 mut (1) in 412 samples. MDM4 is known to be essential if TP53 is functional -TP53 wt (0)

References (supplementary material)

1. Nilsen TW, Graveley BR. Expansion of the eukaryotic proteome by alternative splicing. Nature [Internet]. 2010;463:457–63.
2. Park E, Pan Z, Zhang Z, Lin L, Xing Y. The expanding landscape of alternative splicing variation in human populations. Am J Hum Genet. The Authors; 2018;102:11–26. doi:10.1016/j.ajhg.2017.11.002
3. Sebestyén E, Singh B, Miñana B, Pagès A, Mateo F, Pujana MA, et al. Large-scale analysis of genome and transcriptome alterations in multiple tumors unveils novel cancer-relevant splicing networks. Genome Res. 2016;26:732–44.
4. Sveen A, Kilpinen S, Ruusulehto A, Lothe R a, Skotheim RI. Aberrant RNA splicing in cancer; expression changes and driver mutations of splicing factor genes. Oncogene [Internet]. Nature Publishing Group; 2015;35:1–15. doi:10.1038/onc.2015.318%5Cn
5. Ladomery M. Aberrant alternative splicing is another hallmark of cancer. Int J Cell Biol. Hindawi Publishing Corporation; 2013;2013.
6. Oltean S, Bates DO. Hallmarks of alternative splicing in cancer. Oncogene [Internet]. Nature Publishing Group; 2014;33:5311–8. doi:10.1038/onc.2013.533
7. Garcia-Blanco MA, Baraniak AP, Lasda EL. Alternative splicing in disease and therapy. Nat Biotechnol. 2004;22:535–46.
8. Safikhani Z, Smirnov P, Thu KL, Silvester J, El-Hachem N, Quevedo R, et al. Gene isoforms as expression-based biomarkers predictive of drug response in vitro. Nat Commun. 2017;8.
9. Carazo F, Romero JP, Rubio Á. Upstream analysis of alternative splicing: a review of computational approaches to predict context-dependent splicing factors. Brief Bioinform. 2018;50.
10. Vitting-Seerup K, Sandelin A. The Landscape of Isoform Switches in Human Cancers. Mol Cancer Res [Internet]. 2017;15:1206–21. doi:10.1158/1541-7786.MCR-16-0459
11. Climente-Gonzalez H, Porta-Pardo E, Godzik A, Eyras E. The Functional Impact of Alternative Splicing in Cancer. 2017;2215–26.
12. Cowley GS, Weir BA, Vazquez F, Tamayo P, Scott J, Rusin S, et al. Parallel genome-scale loss of function screens in 216 cancer cell lines for the identification of context-specific genetic dependencies. Sci data. 2014;1:140035.
13. Tsherniak A, Vazquez F, Montgomery PG, Weir BA, Kryukov G, Cowley GS, et al. Defining a Cancer Dependency Map. Cell. Elsevier Inc.; 2017;170:564–576.e16. doi:10.1016/j.cell.2017.06.010
14. Shao DD, Tsherniak A, Gopal S, Weir BA, Tamayo P, Stransky N, et al. ATARiS: Computational quantification of gene suppression phenotypes from multisample RNAi screens. Genome Res. 2013;23:665–78.
15. Hart T, Brown KR, Sircoulomb F, Rottapel R, Moffat J. Measuring error rates in genomic perturbation screens: gold standards for human functional genomics. Mol Syst Biol [Internet]. 2014;10:733–733. Available from: http://msb.embopress.org/cgi/doi/10.15252/msb.20145216
16. Mcfarland JM, Ho Z V, Kugener G, Dempster JM, Montgomery PG, Bryan JG, et al. Improved estimation of cancer dependencies from large-scale RNAi screens using model- based normalization and data integration. bioRxiv [Internet]. 2018;305656. https://www.biorxiv.org/content/early/2018/04/24/305656.abstract%0Apapers3://publication/doi/10.1101/305656
17. Aguirre AJ, Meyers RM, Weir BA, Vazquez F, Zhang CZ, Ben-David U, et al. Genomic copy number dictates a gene-independent cell response to CRISPR/Cas9 targeting. Cancer Discov. 2016;6:914–29.
18. Ritchie ME, Phipson B, Wu D, Hu Y, Law CW, Shi W, et al. Limma powers differential expression analyses for RNA-sequencing and microarray studies. Nucleic Acids Res. 2015;43:e47.
19. Weinstein IB, Joe A. Oncogene addiction. Cancer Res. 2008;68:3077–80.
20. Toledo F, Wahl GM. MDM2 and MDM4: p53 regulators as targets in anticancer therapy. Int J Biochem Cell Biol. 2007;39:1476–82.
21. R Development Core Team. R: a language and environment for statistical computing. [http://wwwRproject.org]. 2003.
22. Chang W, Cheng J, Allaire J, Xie Y, McPherson J. shiny: Web application framework for R. [http://CRAN R-project org/package= shiny]. 2017;
23. Verbeke T, Michielssen F. ShinyProxy--open source enterprise deployment for shiny. GitHub Repos. 2016;
24. Zerbino DR, Achuthan P, Akanni W, Amode MR, Barrell D, Bhai J, et al. Ensembl 2018. Nucleic Acids Res. 2018;46:D754–61.

25. Dvinge H, Bradley RK. Widespread intron retention diversifies most cancer transcriptomes. Genome Med. Genome Medicine; 2015;7:1–13. doi:10.1186/s13073-015-0168-9
26. Xi X, Li T, Huang Y, Sun J, Zhu Y, Yang Y, et al. RNA Biomarkers: Frontier of Precision Medicine for Cancer. Non-Coding RNA [Internet]. 2017;3:9. Available from: http://www.mdpi.com/2311-553X/3/1/9
27. Smart AC, Margolis CA, Pimentel H, He MX, Miao D, Adeegbe D, et al. Intron retention as a novel source of cancer neoantigens. bioRxiv. 2018;309450. https://www.biorxiv.org/content/early/2018/04/27/309450
28. Braunschweig U, Barbosa-Morais NL, Pan Q, Nachman EN, Alipanahi B, Gonatopoulos-Pournatzis T, et al. Widespread intron retention in mammals functionally tunes transcriptomes. Genome Res. 2014;24:1774–86.
29. Liu X, Wang J, Sun G. Identification of key genes and pathways in renal cell carcinoma through expression profiling data. Kidney Blood Press Res. 2015;40:288–97.
30. Abbott KL, Nyre ET, Abrahante J, Ho YY, Vogel RI, Starr TK. The candidate cancer gene database: A database of cancer driver genes from forward genetic screens in mice. Nucleic Acids Res. 2015;43:D844–8.
31. Clissold RL, Hamilton AJ, Hattersley AT, Ellard S, Bingham C. HNF1B-associated renal and extra-renal disease—an expanding clinical spectrum. Nat Rev Nephrol. 2014;11:102–12. doi:10.1038/nrneph.2014.232
32. Chang A, Brimo F, Montgomery EA, Epstein JI. Use of PAX8 and GATA3 in diagnosing sarcomatoid renal cell carcinoma and sarcomatoid urothelial carcinoma. Hum Pathol. Elsevier Inc.; 2013;44:1563–8. doi:10.1016/j.humpath.2012.12.012
33. Robson EJD, He SJ, Eccles MR. A PANorama of PAX genes in cancer and development. Nat Rev Cancer. 2006;6:52–62.
34. Dressler GR, Wilkinson JE, Rothenpieler UW, Patterson LT, Williams-Simons L, Westphal H. Deregulation of Pax-2 expression in transgenic mice generates severe kidney abnormalities. Nature. Nature Publishing Group; 1993;362:65.
35. Bouchard M, Souabni A, Mandler M, Neubüser A, Busslinger M. Nephric lineage specification by Pax2 and Pax8. Genes Dev. 2002;16:2958–70.
36. Rebouissou S, Vasiliu V, Thomas C, Bellanné-Chantelot C, Bui H, Chrétien Y, et al. Germline hepatocyte nuclear factor 1α and 1β mutations in renal cell carcinomas. Hum Mol Genet. 2005;14:603–14.
37. Rhyasen GW, Starczynowski DT. IRAK signalling in cancer. Br J Cancer [Internet]. Nature Publishing Group; 2015;112:232–7. doi:10.1038/bjc.2014.513
38. De Nardo D, Masendycz P, Ho S, Cross M, Fleetwood AJ, Reynolds EC, et al. A central role for the Hsp90·Cdc37 molecular chaperone module in interleukin-1 receptor-associated-kinase-dependent signaling by Toll-like receptors. J Biol Chem. 2005;280:9813–22.
39. Roberts PJ, Der CJ. Targeting the Raf-MEK-ERK mitogen-activated protein kinase cascade for the treatment of cancer. Oncogene. 2007;26:3291–310.
40. McDermott EP, O'Neill LAJ. Ras participates in the activation of p38 MAPK by interleukin-1 by associating with IRAK, IRAK2, TRAF6, and TAK-1. J Biol Chem. 2002;277:7808–15.
41. Barretina J, Caponigro G, Stransky N, Venkatesan K, Margolin A a, Kim S, et al. The Cancer Cell Line Encyclopedia enables predictive modelling of anticancer drug sensitivity Supp. Nature [Internet]. 2012;483:603–7.
42. Tatlow PJ, Piccolo SR. A cloud-based workflow to quantify transcript-expression levels in public cancer compendia. Sci Rep [Internet]. 2016;6:39259.
43. Bray NL, Pimentel H, Melsted P, Pachter L. Near-optimal probabilistic RNA-seq quantification. Nat Biotechnol [Internet]. 2016;34:525–7. doi:10.1038/nbt.3519
44. Harrow J, Frankish A, Gonzalez JM, Tapanari E, Diekhans M, Kokocinski F. GENCODE: The Reference Human Genome Annotation for The ENCODE Project. Genome Res. 2012;22:1760–74. Available from: doi:10.1101/gr.135350.111
45. Brown P, Hastie T, Tibshirani R, Botstein D, Altman RB. Missing value estimation methods for DNA microarrays. Bioinformatics. 2001;17:520–5.
46. Ignatiadis N, Klaus B, Zaugg JB, Huber W. Data-driven hypothesis weighting increases detection power in genome-scale multiple testing. Nat Methods. 2016;13:577–80.
47. Efron B, Efron B, Tibshirani R, Tibshirani R. Empirical bayes method and false discovery rates for microarrays. Genet Epidemiol. 2002;23:70–86.
48. Storey JD. A direct approach to false discovery rates. J R Stat Soc Ser B Stat Methodol. 2002;64:479–

98.
49. Jaiswal A, Peddinti G, Akimov Y, Wennerberg K, Kuznetsov S, Tang J, et al. Seed-effect modeling improves the consistency of genome-wide loss-of-function screens and identifies synthetic lethal vulnerabilities in cancer cells. Genome Med. Genome Medicine; 2017;9:51. Available from: doi:10.1186/s13073-017-0440-2
50. Meyers RM, Bryan JG, McFarland JM, Weir BA, Sizemore AE, Xu H, et al. Computational correction of copy number effect improves specificity of CRISPR-Cas9 essentiality screens in cancer cells. Nat Genet. 2017;49:1779–84.
51. Ritchie W, Granjeaud S, Puthier D, Gautheret D. Entropy measures quantify global splicing disorders in cancer. PLoS Comput Biol. 2008;4:1–9.
52. Pertea M, Pertea GM, Antonescu CM, Chang T-C, Mendell JT, Salzberg SL. StringTie enables improved reconstruction of a transcriptome from RNA-seq reads. Nat Biotechnol. 2015;33:290–5.
53. Trapnell C, Williams BA, Pertea G, Mortazavi A, Kwan G, van Baren MJ, et al. Transcript assembly and quantification by RNA-Seq reveals unannotated transcripts and isoform switching during cell differentiation. Nat Biotechnol. Nature Publishing Group; 2010;28:511–5. Available from: doi:10.1038/nbt.1621
54. Trapnell C, Hendrickson DG, Sauvageau M, Goff L, Rinn JL, Pachter L. Differential analysis of gene regulation at transcript resolution with RNA-seq. Nat Biotechnol. Nature Publishing Group; 2013;31:46–53.
55. Steijger T, Abril JF, Engström PG, Kokocinski F, Hubbard TJ, Guigó R, et al. Assessment of transcript reconstruction methods for RNA-seq. Nat Methods. Nature Publishing Group; 2013;10:1177–84.
56. Mazzoccoli G, Piepoli A, Carella M, Panza A, Pazienza V, Benegiamo G, et al. Altered expression of the clock gene machinery in kidney cancer patients. Biomed Pharmacother [Internet]. Elsevier Masson SAS; 2012;66:175–9. doi:10.1016/j.biopha.2011.11.007
