# Supplementary material for: TranscriptAchilles: a genome-wide platform to predict isoform biomarkers of gene essentiality in cancer
Source: Gigascience. 2019 Apr 3;8(4):giz021. doi: 10.1093/gigascience/giz021 (PMC6446222; doi:10.1093/gigascience/giz021)
Supplement: Supplement_File.pdf [file giz021_supplement_file.pdf]

Supplementary Material

TranscriptAchilles: a genome-wide platform to predict transcript biomarkers and drug target genes in cancer

Fernando Carazo[1], Lucía Campuzano[2], Xabier Cendoya[1], Francisco J. Planes[1] and Angel Rubio[1]*

1 Tecnun (University of Navarra), Paseo Manuel Lardizábal 15, 20018 San Sebastián, SPAIN

2 University of Luxembourg, 2, avenue de l'Université, 4365 Esch-sur-Alzette, LUXEMBOURG

** Corresponding author: Angel Rubio, e-mail: arubio@tecnun.es*

SECTION 1. Quick start

The page of the web-app contains an *Overview* panel with detailed information about the tool and the pipeline for performing an analysis with the app. In the *Help* panel, further details of the different features can be found. The functionalities of TranscriptAchilles are presented in a set of panels in the app. Figure S1 shows the pipeline of TranscriptAchilles.

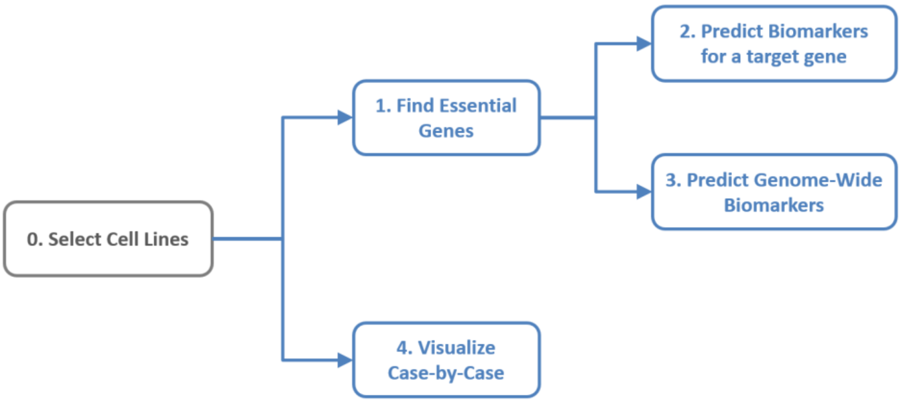

Figure S1. Quick start: pipeline

The main panels of the platform are:

0. Select cell lines. The user can select the cohort of cell lines to be analyzed. Several primary sites and subtypes can be selected at the same time. The application is pre-loaded with all the necessary data, so that the user does not need to upload any file.

1. Find Essential Genes. TranscriptAchilles identifies putative drug targets for the selected cell lines. Essential genes are required to meet several criteria: 1) they must be essential for a minimum percentage of samples in the selected subtype, 2) they must be specific for the subtype under study and 3) they must be expressed. In order to achieve these three requirements, the user must set several thresholds. The first one is the percentage of cell lines that are sensitive to the gene knockdown of interest. The second one is an odds ratio, which can be illustrated with an example: if the enrichment is set to 2, the percentage of cell lines sensitive to the gene knockdown must be two times larger for the cell lines under study than for the rest of cell lines in the DEMETER dataset. Finally, a threshold on minimum TPM (transcripts per million) expression can be set to ensure that the gene is expressed.

2. Predict Biomarkers for a Target Gene. In this section, the user can select one or more genes from the previous step and predict putative biomarkers of their essentiality. The statistical model estimates the local false discovery rate for both genes and transcripts and decides whether genes or transcripts are the best markers for each case (see Methods section of the main manuscript).

3. Predict Genome-Wide Biomarkers. In this case the biomarkers are found for all the essential genes identified in the step *Find Essential Genes.*

4. Visualize Case-by-Case. The user can visualize the essentiality of any gene and transcript biomarker. This panel can be run once the cell lines are selected.

0. Select cell lines

The user is required to select the cohort of cell lines to be analyzed. Several primary sites and subtypes can be selected at the same time. The application is pre-loaded with all the necessary data, so that the user does not need to upload any data.

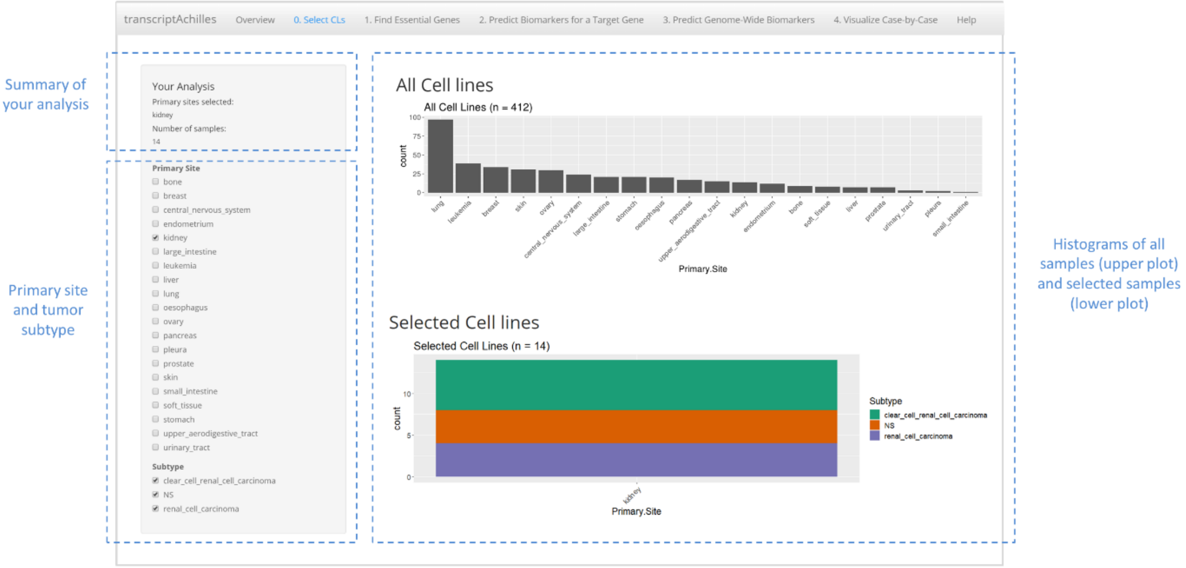

Figure S2. Quick start: selection of samples

1. Find essential genes

The second step of the analysis consists in extracting the essential gene list for the selected cell lines. Five tunable parameters allow the user to tailor the output. The toolbox provides a default value for each parameter. The assigned values correspond to our understanding of the minimum conditions which need to be satisfied by a gene to be essential.

The filters correspond to three criteria: essentiality, specificity and expression. Essentiality is a two-legged characteristic. It refers to the percentage of selected cell lines that have a DEMETER score lower than the essentiality cut-off. Specificity is represented by the enrichment ratio. This filter allows the user to define the minimum ratio between the proportion of selected cell lines for which a gene is essential and the proportion of the rest of the cell lines for which the same gene is essential.

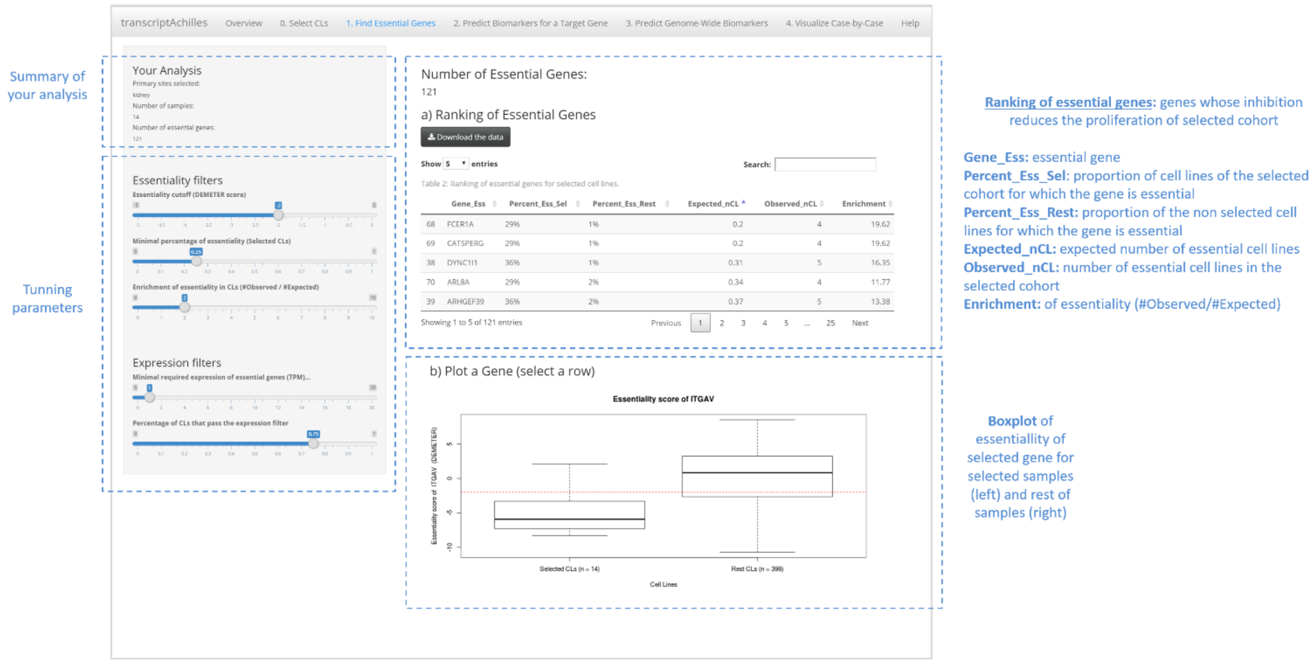

Figure S3. Quick start: essential genes

2. Predict biomarkers for a target gene

In this section, the user can select one or more genes of the previous step and predict putative biomarkers for their essentiality. In each case, the application decides whether genes or transcripts are the best markers.

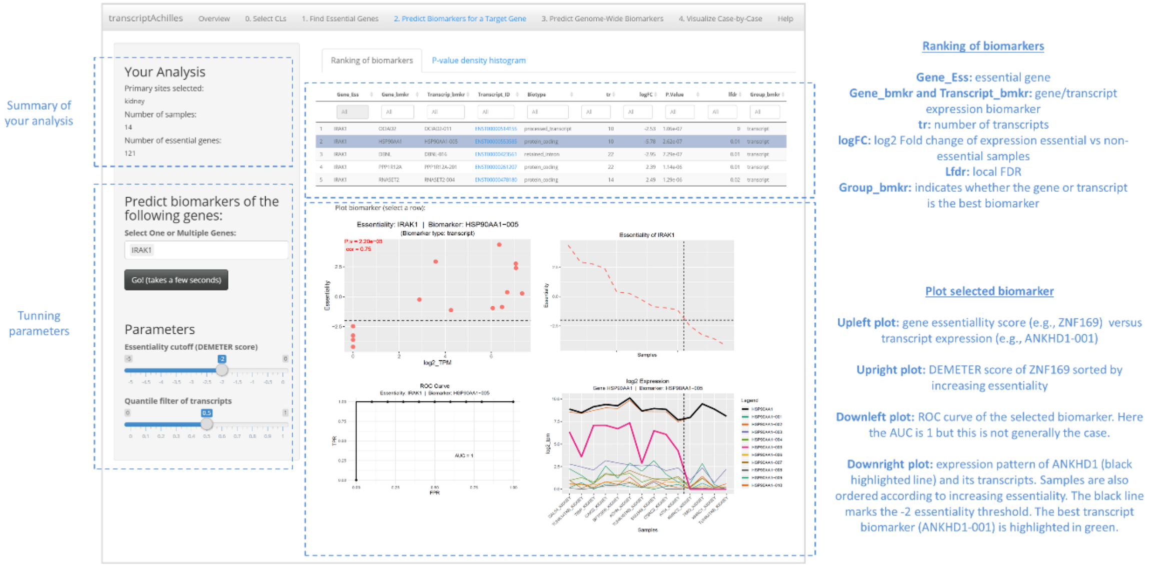

Figure S4. Quick start: prediction of transcript biomarkers

3. Predict Genome-Wide Biomarkers.

In this case the biomarkers are found for all the essential genes identified in the step *Find Essential Genes.*

4. Visualize Case-by-Case.

The user can also visualize the essentiality of any gene and transcript biomarker. This panel can be run once the cell lines are selected

SECTION 2. Other examples of TranscriptAchilles

Three examples of TranscriptAchilles in kidney carcinoma:

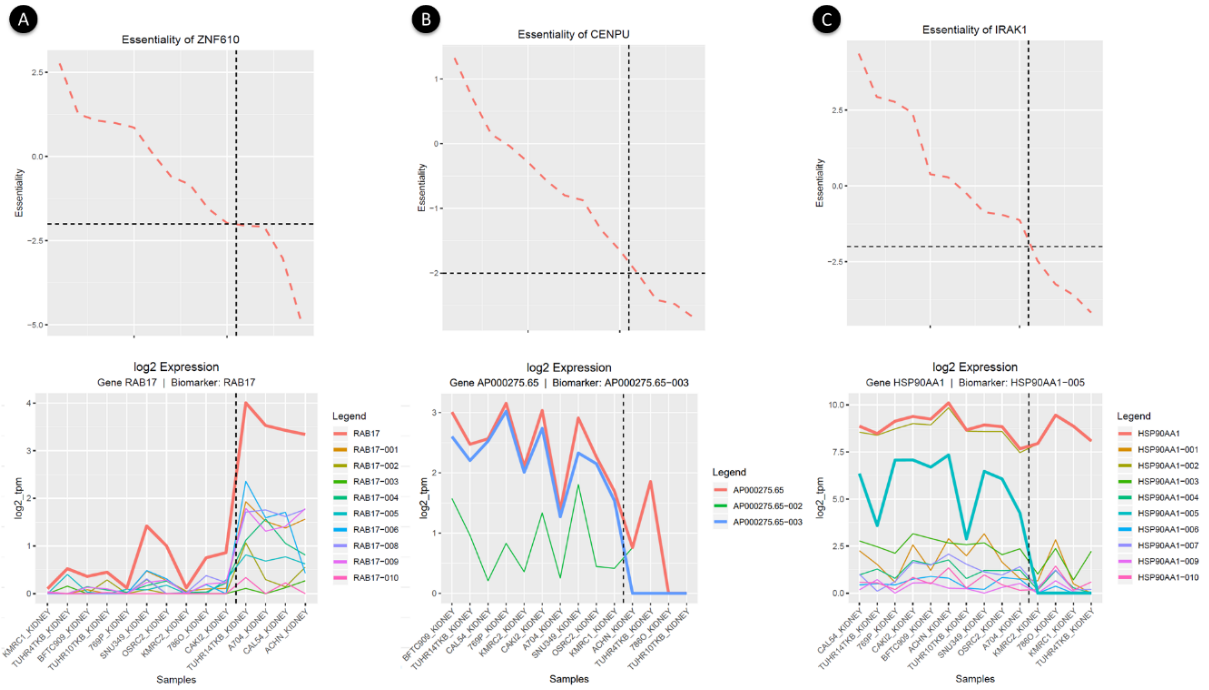

Figure S5. Three examples of TranscriptAchilles in kidney carcinoma (n = 14). In each example, the essentiality of a gene for every cell line and the log2 expression values of the gene biomarker are shown in the upper and lower plot, respectively. The cell lines are ordered according to increasing essentiality. The vertical dotted line separates the cell lines into resistant (left) and sensitive (right) to the inhibition of the essential gene (DEMETER score < -2). Gene expression is highlighted in red. The best transcript biomarker is also highlighted. When the best biomarker is the gene, no transcript is highlighted. ***A)*** *Essentiality of ZNF610. The biomarker is the gene expression of RAB17.* ***B)*** *Essentiality of CENPU. The best biomarker is isoform AP000275.65-003.* ***C)*** *Essentiality of IRAK1. The isoform biomarker is not the most expressed isoform. Gene expression is not a good biomarker. However, there is a clear expression change in Isoform HSP90AA1-005.*

Once essential genes are identified the tool allows the prediction of companion biomarkers, as none of the putative target genes is essential for 100% of renal cell lines. These biomarkers (gene or transcript) are obtained by running the Predict Genome-Wide Biomarkers section of the tool.

PER3 belongs to the period circadian regulator family, which is reported to be dysregulated in kidney tumors [56]. SEC31A is expressed by all renal cell lines, but its transcript SEC31A-020 is absent in sensitive cell lines.

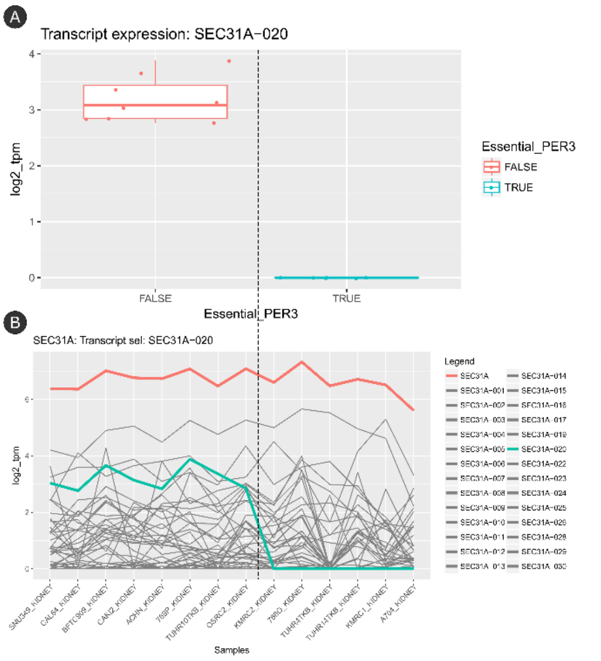

Figure S6. A) log2-expression boxplot of the predicted transcript biomarker (SEC31A-020) in renal cancer cell lines (n = 14). PER3 sensitive (red) and resistant (blue) cell lines are shown. B) Expression pattern of gene SEC31A (red highlighted line) and its transcripts. Samples are ordered according to increasing essentiality. The black line marks the -2 essentiality threshold. The best transcript biomarker (SEC31A-020) is highlighted in blue.

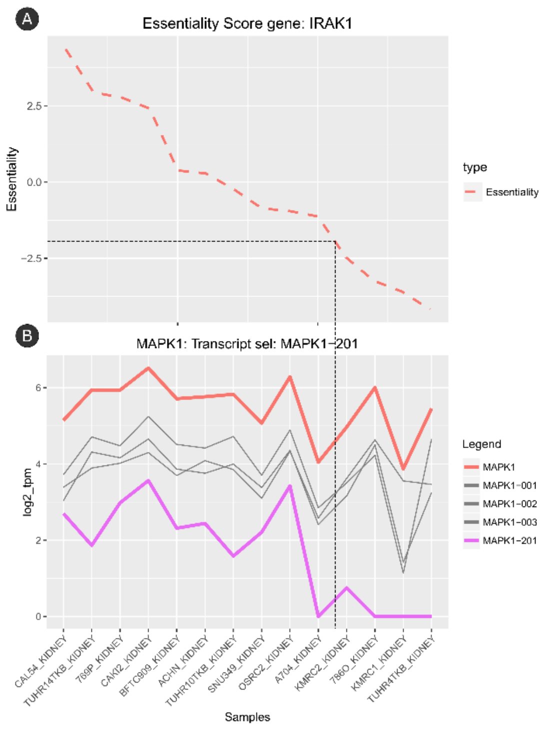

Figure S7. Predicted target gene (IRAK1) in renal carcinoma cell lines (n = 14) with its companion biomarker (transcript MAPK1-201). A) renal cell lines ordered by increasing essentiality of IRAK1.. The dotted black line marks the default essentiality score of -2. B) Expression pattern of gene MAPK1 (red highlighted line) and its transcripts. Samples are ordered according to increasing essentiality of IRAK1. The dotted black line marks the -2 essentiality threshold dividing cell lines into resistant (left side) and sensitive (right side). The best transcript biomarker (MAPK1-201) is highlighted in purple. In this case, transcript expression is a better marker of essentiality than gene expression.

SECTION 3. Positive controls of DEMETER

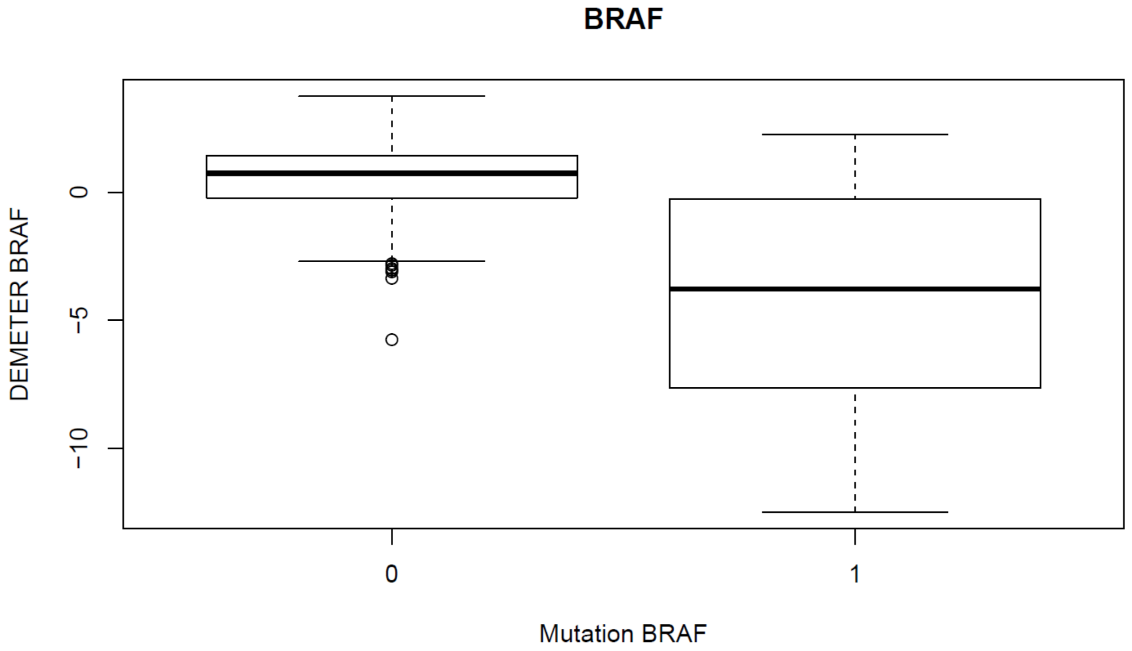

Figure S8. BRAF oncogene. Essentiality of BRAF for BRAF wt (0) and BRAF mut (1) in 412 samples.

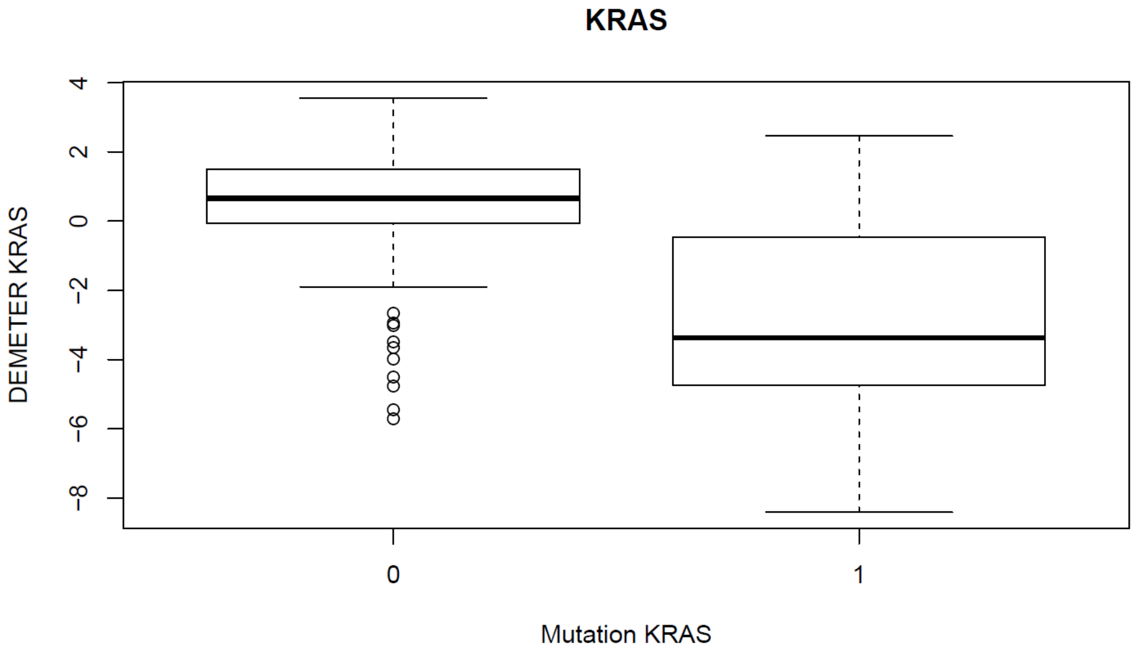

Figure S9. KRAS oncogene. Essentiality of KRAS for KRAF wt (0) and KRAS mut (1) in 412 samples.

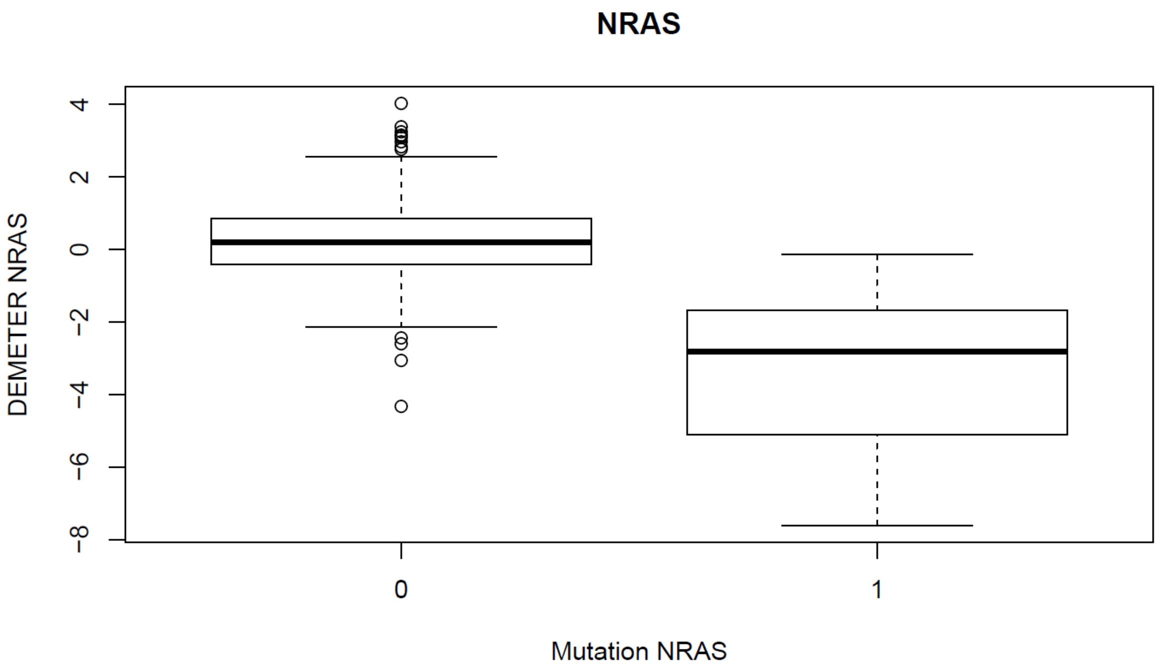

Figure S10. NRAS oncogene. Essentiality of NRAS for NRAS wt (0) and NRAS mut (1) in 412 samples.

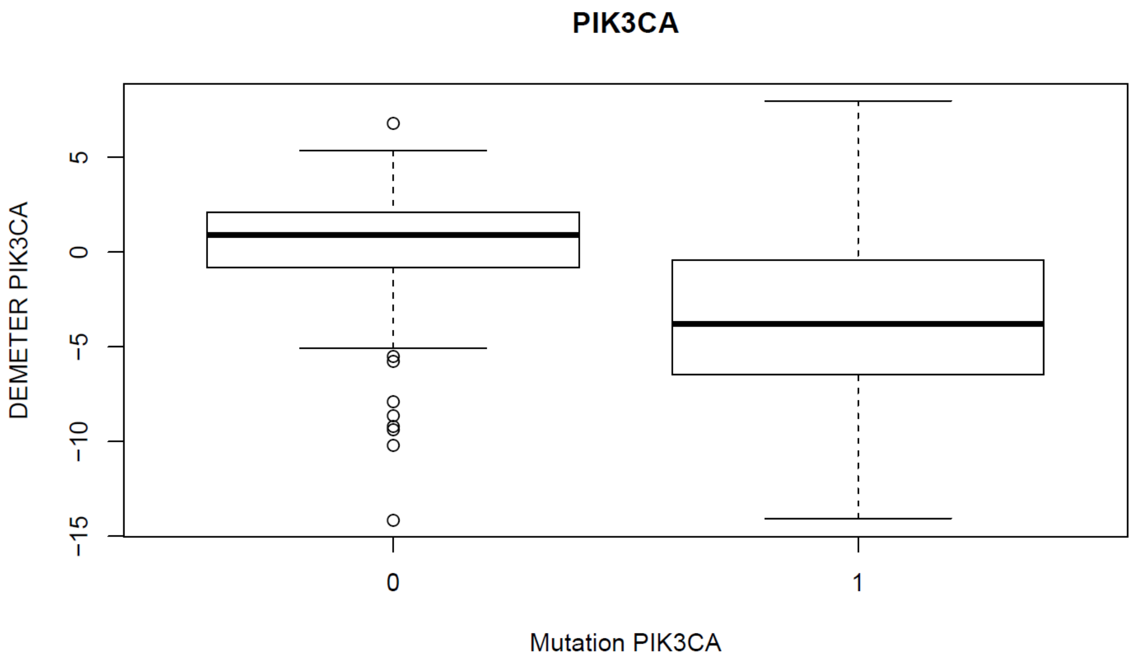

Figure S11. PIK3CA oncogene. Essentiality of PIK3CA for PIK3CA wt (0) and PIK3CA mut (1) in 412 samples.

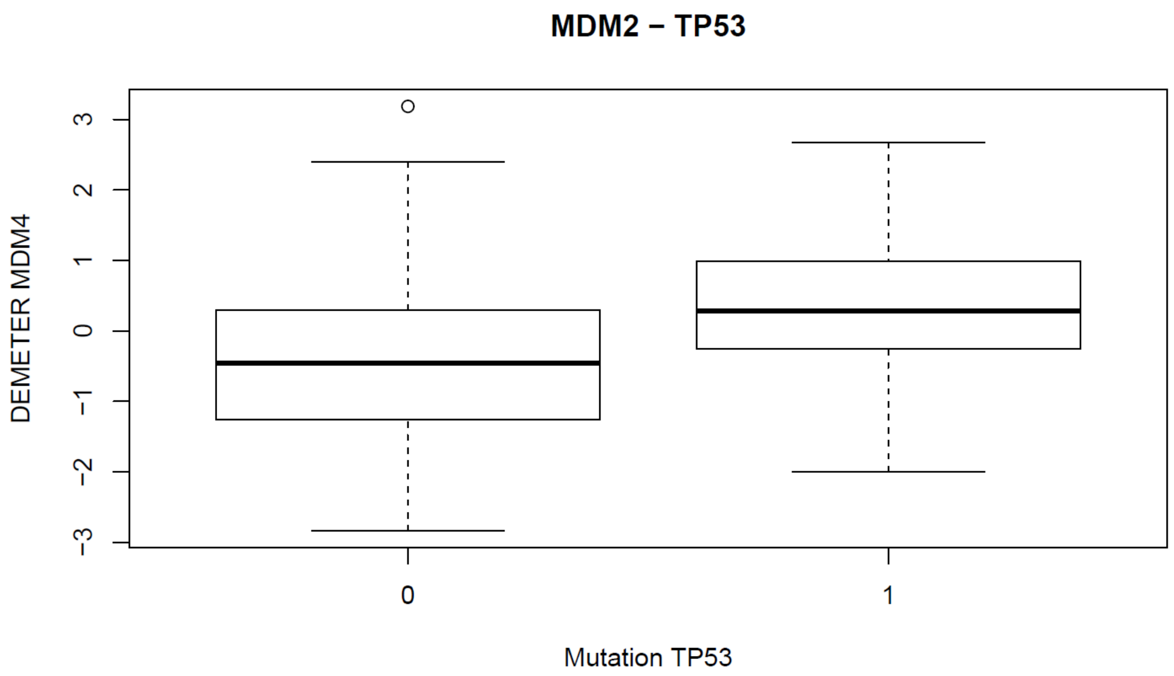

Figure S12. TP53 mutation and MDM2. Essentiality of MDM2 for TP53 wt (0) and MDM2 mut (1) in 412 samples. MDM2 is known to be essential if TP53 is functional -TP53 wt (0).

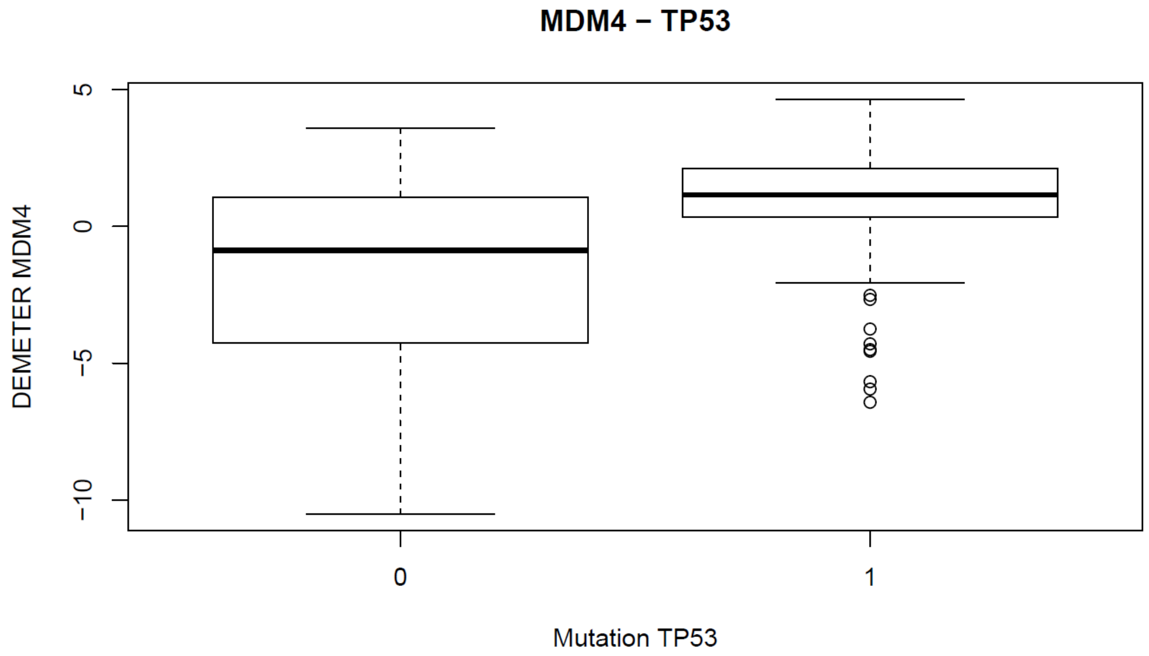

Figure S13. TP53 mutation and MDM4. Essentiality of MDM4 for TP53 wt (0) and MDM4 mut (1) in 412 samples. MDM4 is known to be essential if TP53 is functional -TP53 wt (0)

References (supplementary material)

1. Nilsen TW, Graveley BR. Expansion of the eukaryotic proteome by alternative splicing. Nature [Internet]. 2010;463:457–63.

2. Park E, Pan Z, Zhang Z, Lin L, Xing Y. The expanding landscape of alternative splicing variation in human populations. Am J Hum Genet. The Authors; 2018;102:11–26. doi:10.1016/j.ajhg.2017.11.002

3. Sebestyén E, Singh B, Miñana B, Pagès A, Mateo F, Pujana MA, et al. Large-scale analysis of genome and transcriptome alterations in multiple tumors unveils novel cancer-relevant splicing networks. Genome Res. 2016;26:732–44.

4. Sveen A, Kilpinen S, Ruusulehto A, Lothe R a, Skotheim RI. Aberrant RNA splicing in cancer; expression changes and driver mutations of splicing factor genes. Oncogene [Internet]. Nature Publishing Group; 2015;35:1–15. doi:10.1038/onc.2015.318%5Cn

5. Ladomery M. Aberrant alternative splicing is another hallmark of cancer. Int J Cell Biol. Hindawi Publishing Corporation; 2013;2013.

6. Oltean S, Bates DO. Hallmarks of alternative splicing in cancer. Oncogene [Internet]. Nature Publishing Group; 2014;33:5311–8. doi:10.1038/onc.2013.533

7. Garcia-Blanco MA, Baraniak AP, Lasda EL. Alternative splicing in disease and therapy. Nat Biotechnol. 2004;22:535–46.

8. Safikhani Z, Smirnov P, Thu KL, Silvester J, El-Hachem N, Quevedo R, et al. Gene isoforms as expression-based biomarkers predictive of drug response in vitro. Nat Commun. 2017;8.

9. Carazo F, Romero JP, Rubio Á. Upstream analysis of alternative splicing: a review of computational approaches to predict context-dependent splicing factors. Brief Bioinform. 2018;50.

10. Vitting-Seerup K, Sandelin A. The Landscape of Isoform Switches in Human Cancers. Mol Cancer Res [Internet]. 2017;15:1206–21. doi:10.1158/1541-7786.MCR-16-0459

11. Climente-Gonzalez H, Porta-Pardo E, Godzik A, Eyras E. The Functional Impact of Alternative Splicing in Cancer. 2017;2215–26.

12. Cowley GS, Weir BA, Vazquez F, Tamayo P, Scott J, Rusin S, et al. Parallel genome-scale loss of function screens in 216 cancer cell lines for the identification of context-specific genetic dependencies. Sci data. 2014;1:140035.

13. Tsherniak A, Vazquez F, Montgomery PG, Weir BA, Kryukov G, Cowley GS, et al. Defining a Cancer Dependency Map. Cell. Elsevier Inc.; 2017;170:564–576.e16. doi:10.1016/j.cell.2017.06.010

14. Shao DD, Tsherniak A, Gopal S, Weir BA, Tamayo P, Stransky N, et al. ATARiS: Computational quantification of gene suppression phenotypes from multisample RNAi screens. Genome Res. 2013;23:665–78.

15. Hart T, Brown KR, Sircoulomb F, Rottapel R, Moffat J. Measuring error rates in genomic perturbation screens: gold standards for human functional genomics. Mol Syst Biol [Internet]. 2014;10:733–733. Available from: http://msb.embopress.org/cgi/doi/10.15252/msb.20145216

16. Mcfarland JM, Ho Z V, Kugener G, Dempster JM, Montgomery PG, Bryan JG, et al. Improved estimation of cancer dependencies from large-scale RNAi screens using model- based normalization and data integration. bioRxiv [Internet]. 2018;305656. https://www.biorxiv.org/content/early/2018/04/24/305656.abstract%0Apapers3://publication/doi/10.1101/305656

17. Aguirre AJ, Meyers RM, Weir BA, Vazquez F, Zhang CZ, Ben-David U, et al. Genomic copy number dictates a gene-independent cell response to CRISPR/Cas9 targeting. Cancer Discov. 2016;6:914–29.

18. Ritchie ME, Phipson B, Wu D, Hu Y, Law CW, Shi W, et al. Limma powers differential expression analyses for RNA-sequencing and microarray studies. Nucleic Acids Res. 2015;43:e47.

19. Weinstein IB, Joe A. Oncogene addiction. Cancer Res. 2008;68:3077–80.

20. Toledo F, Wahl GM. MDM2 and MDM4: p53 regulators as targets in anticancer therapy. Int J Biochem Cell Biol. 2007;39:1476–82.

21. R Development Core Team. R: a language and environment for statistical computing. [http://wwwRproject.org]. 2003.

22. Chang W, Cheng J, Allaire J, Xie Y, McPherson J. shiny: Web application framework for R. [http://CRAN R-project org/package= shiny]. 2017;

23. Verbeke T, Michielssen F. ShinyProxy--open source enterprise deployment for shiny. GitHub Repos. 2016;

24. Zerbino DR, Achuthan P, Akanni W, Amode MR, Barrell D, Bhai J, et al. Ensembl 2018. Nucleic Acids Res. 2018;46:D754–61.

25. Dvinge H, Bradley RK. Widespread intron retention diversifies most cancer transcriptomes. Genome Med. Genome Medicine; 2015;7:1–13. doi:10.1186/s13073-015-0168-9
26. Xi X, Li T, Huang Y, Sun J, Zhu Y, Yang Y, et al. RNA Biomarkers: Frontier of Precision Medicine for Cancer. Non-Coding RNA [Internet]. 2017;3:9. Available from: http://www.mdpi.com/2311-553X/3/1/9
27. Smart AC, Margolis CA, Pimentel H, He MX, Miao D, Adeegbe D, et al. Intron retention as a novel source of cancer neoantigens. bioRxiv. 2018;309450. https://www.biorxiv.org/content/early/2018/04/27/309450
28. Braunschweig U, Barbosa-Morais NL, Pan Q, Nachman EN, Alipanahi B, Gonatopoulos-Pournatzis T, et al. Widespread intron retention in mammals functionally tunes transcriptomes. Genome Res. 2014;24:1774–86.
29. Liu X, Wang J, Sun G. Identification of key genes and pathways in renal cell carcinoma through expression profiling data. Kidney Blood Press Res. 2015;40:288–97.
30. Abbott KL, Nyre ET, Abrahante J, Ho YY, Vogel RI, Starr TK. The candidate cancer gene database: A database of cancer driver genes from forward genetic screens in mice. Nucleic Acids Res. 2015;43:D844–8.
31. Clissold RL, Hamilton AJ, Hattersley AT, Ellard S, Bingham C. HNF1B-associated renal and extra-renal disease—an expanding clinical spectrum. Nat Rev Nephrol. 2014;11:102–12. doi:10.1038/nrneph.2014.232
32. Chang A, Brimo F, Montgomery EA, Epstein JI. Use of PAX8 and GATA3 in diagnosing sarcomatoid renal cell carcinoma and sarcomatoid urothelial carcinoma. Hum Pathol. Elsevier Inc.; 2013;44:1563–8. doi:10.1016/j.humpath.2012.12.012
33. Robson EJD, He SJ, Eccles MR. A PANorama of PAX genes in cancer and development. Nat Rev Cancer. 2006;6:52–62.
34. Dressler GR, Wilkinson JE, Rothenpieler UW, Patterson LT, Williams-Simons L, Westphal H. Deregulation of Pax-2 expression in transgenic mice generates severe kidney abnormalities. Nature. Nature Publishing Group; 1993;362:65.
35. Bouchard M, Souabni A, Mandler M, Neubüser A, Busslinger M. Nephric lineage specification by Pax2 and Pax8. Genes Dev. 2002;16:2958–70.
36. Rebouissou S, Vasiliu V, Thomas C, Bellanné-Chantelot C, Bui H, Chrétien Y, et al. Germline hepatocyte nuclear factor 1α and 1β mutations in renal cell carcinomas. Hum Mol Genet. 2005;14:603–14.
37. Rhyasen GW, Starczynowski DT. IRAK signalling in cancer. Br J Cancer [Internet]. Nature Publishing Group; 2015;112:232–7. doi:10.1038/bjc.2014.513
38. De Nardo D, Masendycz P, Ho S, Cross M, Fleetwood AJ, Reynolds EC, et al. A central role for the Hsp90·Cdc37 molecular chaperone module in interleukin-1 receptor-associated-kinase-dependent signaling by Toll-like receptors. J Biol Chem. 2005;280:9813–22.
39. Roberts PJ, Der CJ. Targeting the Raf-MEK-ERK mitogen-activated protein kinase cascade for the treatment of cancer. Oncogene. 2007;26:3291–310.
40. McDermott EP, O'Neill LAJ. Ras participates in the activation of p38 MAPK by interleukin-1 by associating with IRAK, IRAK2, TRAF6, and TAK-1. J Biol Chem. 2002;277:7808–15.
41. Barretina J, Caponigro G, Stransky N, Venkatesan K, Margolin A a, Kim S, et al. The Cancer Cell Line Encyclopedia enables predictive modelling of anticancer drug sensitivity Supp. Nature [Internet]. 2012;483:603–7.
42. Tatlow PJ, Piccolo SR. A cloud-based workflow to quantify transcript-expression levels in public cancer compendia. Sci Rep [Internet]. 2016;6:39259.
43. Bray NL, Pimentel H, Melsted P, Pachter L. Near-optimal probabilistic RNA-seq quantification. Nat Biotechnol [Internet]. 2016;34:525–7. doi:10.1038/nbt.3519
44. Harrow J, Frankish A, Gonzalez JM, Tapanari E, Diekhans M, Kokocinski F. GENCODE: The Reference Human Genome Annotation for The ENCODE Project. Genome Res. 2012;22:1760–74. Available from: doi:10.1101/gr.135350.111
45. Brown P, Hastie T, Tibshirani R, Botstein D, Altman RB. Missing value estimation methods for DNA microarrays. Bioinformatics. 2001;17:520–5.
46. Ignatiadis N, Klaus B, Zaugg JB, Huber W. Data-driven hypothesis weighting increases detection power in genome-scale multiple testing. Nat Methods. 2016;13:577–80.
47. Efron B, Efron B, Tibshirani R, Tibshirani R. Empirical bayes method and false discovery rates for microarrays. Genet Epidemiol. 2002;23:70–86.
48. Storey JD. A direct approach to false discovery rates. J R Stat Soc Ser B Stat Methodol. 2002;64:479–

98.
49. Jaiswal A, Peddinti G, Akimov Y, Wennerberg K, Kuznetsov S, Tang J, et al. Seed-effect modeling improves the consistency of genome-wide loss-of-function screens and identifies synthetic lethal vulnerabilities in cancer cells. Genome Med. Genome Medicine; 2017;9:51. Available from: doi:10.1186/s13073-017-0440-2
50. Meyers RM, Bryan JG, McFarland JM, Weir BA, Sizemore AE, Xu H, et al. Computational correction of copy number effect improves specificity of CRISPR-Cas9 essentiality screens in cancer cells. Nat Genet. 2017;49:1779–84.
51. Ritchie W, Granjeaud S, Puthier D, Gautheret D. Entropy measures quantify global splicing disorders in cancer. PLoS Comput Biol. 2008;4:1–9.
52. Pertea M, Pertea GM, Antonescu CM, Chang T-C, Mendell JT, Salzberg SL. StringTie enables improved reconstruction of a transcriptome from RNA-seq reads. Nat Biotechnol. 2015;33:290–5.
53. Trapnell C, Williams BA, Pertea G, Mortazavi A, Kwan G, van Baren MJ, et al. Transcript assembly and quantification by RNA-Seq reveals unannotated transcripts and isoform switching during cell differentiation. Nat Biotechnol. Nature Publishing Group; 2010;28:511–5. Available from: doi:10.1038/nbt.1621
54. Trapnell C, Hendrickson DG, Sauvageau M, Goff L, Rinn JL, Pachter L. Differential analysis of gene regulation at transcript resolution with RNA-seq. Nat Biotechnol. Nature Publishing Group; 2013;31:46–53.
55. Steijger T, Abril JF, Engström PG, Kokocinski F, Hubbard TJ, Guigó R, et al. Assessment of transcript reconstruction methods for RNA-seq. Nat Methods. Nature Publishing Group; 2013;10:1177–84.
56. Mazzoccoli G, Piepoli A, Carella M, Panza A, Pazienza V, Benegiamo G, et al. Altered expression of the clock gene machinery in kidney cancer patients. Biomed Pharmacother [Internet]. Elsevier Masson SAS; 2012;66:175–9. doi:10.1016/j.biopha.2011.11.007
