# Supplementary material for: TranscriptAchilles: a genome-wide platform to predict isoform biomarkers of gene essentiality in cancer
Source: Gigascience. 2019 Apr 3;8(4):giz021. doi: 10.1093/gigascience/giz021 (PMC6446222; doi:10.1093/gigascience/giz021)
Supplement: Response-to-Reviewer-Comments_Original-Submission.pdf [file giz021_response-to-reviewer-comments_original-submission.pdf]

Author's Response To Reviewer Comments

Close

Dear Dr. Rubio,
Apologies for the delay but we now have the final late reviewers report in. Your manuscript "TranscriptAchilles: a genome-wide platform to predict transcript biomarkers and drug target genes in cancer" (GIGA-D-18-00356) has been assessed by our reviewers. Although it is of interest, we are unable to consider it for publication in its current form without some additional work on the reproducibility. The reviewers have raised a number of points which we believe would improve the manuscript and should allow a revised version to be published in GigaScience.
Their reports, together with any other comments, are below. Please also take a moment to check our website at https://giga.editorialmanager.com/ for any additional comments that were saved as attachments.
In addition, please register any new software application in the SciCrunch.org database to receive a RRID (Research Resource Identification Initiative ID) number, and include this in your manuscript. This will facilitate tracking, reproducibility and re-use of your tool.
If you are able to fully address these points, we would encourage you to submit a revised manuscript to GigaScience. Once you have made the necessary corrections, please submit online at:
https://giga.editorialmanager.com/
If you have forgotten your username or password please use the "Send Login Details" link to get your login information. For security reasons, your password will be reset.
Please include a point-by-point within the 'Response to Reviewers' box in the submission system. Please ensure you describe additional experiments that were carried out and include a detailed rebuttal of any criticisms or requested revisions that you disagreed with. Please also ensure that your revised manuscript conforms to the journal style, which can be found in the Instructions for Authors on the journal homepage.
The due date for submitting the revised version of your article is 02 Mar 2019.
I look forward to receiving your revised manuscript soon.
Best wishes,
Scott Edmunds, Ph.D.
GigaScience
www.gigasciencejournal.com

RESPONSE TO THE EDITOR.

Thank you for your comments. We have included a point-by-point response to the reviewers. TranscriptAchilles has been registered in the SciCrunch.org (TranscriptAchilles, RRID: SCR_016849). We have also checked that the manuscript conforms to the journal style.

Best regards,

All the authors.

RESPONSE TO THE REVIEWERS.

Reviewer #1: The authors describe a new software tool for examining relationships between gene essentiality and transcript/gene expression in cell lines. This tool has the potential to help researchers without computational skills to sift through the Achilles data and make novel correlations, potentially to aid in identifying candidate drug biomarkers. The authors have placed an emphasis on ensuring the software is easy to install and use. The software is freely available. Below are some additional comments and

suggestions for improving the manuscript. I have divided these into Major and Minor points.

Major

1. Installation: dependencies change over time, so the exact install code may need to change. It would be better to put this install code on the GitHub site (already there) rather than in the paper.

We agree with the reviewer. The installation code has been moved to the GitLab repository (https://gitlab.com/fcarazo.m/transcriptachilles) and removed from the main paper.

2. The paper has no instructions for executing the app locally within Docker (which can help to avoid R package installation issues).

Thank you for pointing this out. The instructions for running the app locally within Docker have been included in the repository (https://gitlab.com/fcarazo.m/transcriptachilles). It has also been indicated in the main paper.

3. How long will the AWS site be up? Perhaps better is to provide more detailed instructions for running it locally within Docker (see comments above).

In compliance with the standard policy of algorithm publication, we will ensure that the web application is online for at least 2 years following the publication date. In addition, the GitLab repository will not be removed, so users will be able to download it and run it locally.

The instructions to run the algorithm locally within Docker have been detailed in the repository (https://gitlab.com/fcarazo.m/transcriptachilles).

4. I am not convinced that there is statistical evidence that the differences among cancer types (Figure 2) are significant. Also, if such high proportions of genes are potential biomarkers of essentiality, are we narrowing things down enough (maybe it is sensitive but not specific)? Please clarify.

- Regarding the statistical evidence of the differences among cancer types shown in Figure 2, we verified whether the differences within tumor types are significant, and indeed they are (Kruskal-Wallis p-value = 3.18e-16). This result has been included in the main paper.

We also mentioned three conclusions. The first one is that “Kidney carcinoma and colon adenocarcinoma were found to have the highest and the fewest number of driver isoform switches, respectively.” This is simply a fact: in both the reference [1] and in our analysis these two types of cancer are the top and the lowest ranked cancers according to number of switches. Of course, this fact may occur by chance and its probability is $(1/4)^2=1/16$ (four is the number of cancer types shared by both studies).

The second one is that “Lung squamous carcinoma was more affected by splicing switches than lung adenocarcinoma”. Applying a Wilcoxon test, the corresponding p-value is 0.051. This result is on the verge of significance.

The third one is that “within hematological tumors, acute lymphoid leukemia (ALL) has the lowest proportion of transcript biomarkers.” Running a Wilcoxon test between ALL in the other hematological malignancies, the p-value is 2.767e-13

[1] Climente-Gonzalez H, Porta-Pardo E, Godzik A, Eyras E. The Functional Impact of Alternative Splicing in Cancer. 2017;2215–26.

- Regarding the proportion of genes/transcripts, it is important to highlight that the mentioned proportions are not relative to the entire list of genes/transcripts, but to the predicted genes/transcripts biomarkers

(i.e., if certain essential gene has 10 potential biomarkers and 9 out of which are “transcripts” we would say that 90% of its biomarkers are transcripts).

We are aware that this concept could be misunderstood, so we have rewritten this part of the results section.

5. The manuscript (especially toward the beginning is not clear enough on how it defines predictive biomarkers. What exactly is being predicted? It appears it is drug sensitivity, but it also mentions predicting essentiality. Is there an assumption that these are necessarily linked? If so, on what basis. Maybe I am just slow to understand, but please clarify.

We would like to thank the reviewer for this comment. After re-reading the manuscript, we noticed that the terms we used weren’t always clear throughout the paper, which could have been an obstacle for comprehension. We have clarified these concepts in the manuscript and adapted the manuscript’s title.

Our algorithm predicts biomarkers of gene essentiality (i.e., it finds biomarkers -either genes or transcripts- that indicate whether a cell line is able to survive after knock-down a specific gene). The knocked-down genes are selected to be specific to different cancer cell line types.

This knowledge can be used to infer drug sensitivity of targeted inhibitors. These compounds inhibit a specific protein and therefore, gene essentiality is closely related to drug sensitivity (see for example [1] and [2]). The difference between gene knock-down and targeted inhibition is that, in the first case, the target is the RNA and, in the second, the compound targets the protein directly.

[1]. Awad MM, Chu QSC, Gandhi L, Stephenson JJ, Govindan R, Bradford DS, et al. An open-label, phase II study of the polo-like kinase-1 (Plk-1) inhibitor, BI 2536, in patients with relapsed small cell lung cancer (SCLC). Lung Cancer [Internet]. Elsevier Ireland Ltd; 2017;104:126–30. Available from: http://dx.doi.org/10.1016/j.lungcan.2016.12.019

[2]. Iorns E, Lord CJ, Turner N, Ashworth A. Utilizing RNA interference to enhance cancer drug discovery. Nat Rev Drug Discov. 2007;6:556–68.

6. In the case study, the authors used a minimum of 1 TPM. This is an extremely low threshold (likely well within error bounds of no expression). Do the authors have evidence to support that this threshold should be used?

The default threshold of 1 TPM can be considered too low for certain biological processes. For example, in the case of metabolic analyses, this cutoff can be set to 5 TPM for genes that code enzymes.

However, in other biological processes -such as transcription factors or signaling- genes with 1 TPM can trigger their function. Other portals such as EBI-CCLE (https://www.ebi.ac.uk/gxa/experiments/E-MTAB-2770/Results#) or The Human Protein Atlas (https://www.proteinatlas.org/) set the default cutoff as 0.5 TPM and 1TPM respectively.

Nevertheless, in our application this threshold can be modified by the user in case he or she wants to focus on functions that require more expressed genes.

7. In the case study, the authors found 73/121 known cancer genes, including two that are already known to play a role in renal carcinoma. That is a long list, and you are bound to find known cancer genes any time you study cell lines. Is there any experimental evidence that these genes actually drive cancer resistance or sensitivity in renal carcinoma?

Our list of 121 genes is agnostic of whether it influences cancer or not. DEMETER provides scores for 17,098 genes. The Candidate Cancer Gene Database includes 8,024 genes within the DEMETER list. The

proportion of cancer-related genes in the essential genes of the case study (73/121) is significantly larger than the initial list (8,024/17,098) (Fisher hypergeometric test P-value = 1.1e-3). In spite of this low p-value, we agree with the reviewer that a study on cancer cell lines tends to be enriched in genes related to cancer.

Regarding the evidences of PAX8 and HNF1B in resistance or sensitivity, different works have shown the influence of these genes in the development of renal carcinoma. The following paragraph details this relation and it has been included in the main manuscript:

"PAX proteins are transcription factors that regulate cell proliferation and migration of embryonic precursor cells [1]. It has been demonstrated that the depletion of PAX2 by RNAi induces apoptosis in kidney carcinoma [2]. In addition, PAX2 and PAX8 double mutants cells do not exhibit mesenchymal–epithelial transition and in turn lack mesonephric tubules [3]. On the other hand, HNF1B is a transcription factor that acts as a tumor suppressor in renal carcinoma through control of PKHD1 expression [4]."

[1]. Robson EJD, He SJ, Eccles MR. A PANorama of PAX genes in cancer and development. Nat Rev Cancer. 2006;6:52–62.
[2] Dressler GR, Wilkinson JE, Rothenpieler UW, Patterson LT, Williams-Simons L, Westphal H. Deregulation of Pax-2 expression in transgenic mice generates severe kidney abnormalities. Nature 1993;362(6415):65–7
[3] Bouchard M, Souabni A, Mandler M, Neubuser A, Busslinger M. Nephric lineage specification by Pax2 and Pax8. Genes Dev 2002;16(22):2958–70.
[4] Rebouissou, Sandra et al. "Germline Hepatocyte Nuclear Factor 1α and 1β Mutations in Renal Cell Carcinomas." Human Molecular Genetics 14.5 (2005): 603–614. Web.

8. In describing the case study, the authors state that, "expression is not capable of distinguishing between sensitive and resistant groups of CLs." But no data is provided to support this claim.

Fixed. The P-value, the corrected local false discovery rate and the area under the roc curve (AUC) using gene expression in the case study have been incorporated to the main manuscript. As it is said (and shown in Figure 4), gene expression is not a good biomarker (P-value = 0.37; local fdr = 0.7; AUC = 0.63). In contrast, the proposed transcript is a good biomarker of essentiality in this case (P-value = 2.62e-07; local fdr = 0.01; AUC = 1).

9. I am not sure I understand at what point the lfdr values are calculated. Is it for each combination of "essential gene" and potential biomarker? If so, you are bound to have lots of false positives because there is such a high number of combinations for which you could perform this test. You would need some kind of global correction for multiple tests.

Thanks for the feedback. We realized that the statistical methods were not clear enough, so we have rewritten this section and incorporated a new figure.

For each knocked down gene, we consider the following null hypothesis: "the mean expression of a biomarker is identical in resistant and in sensitive cell lines to a gene knock-down". We consider a cell line to be sensitive to a knock-down if DEMETER score < threshold. The default threshold is -2: the threshold proposed by the DEMETER's authors.

To test this hypothesis, we used a moderated t-test using limma. We applied this test for each RNAi target and all the expressed genes and transcripts to get the corresponding p-values.

The advantage of local false discovery rate is that it directly provides the probability of the null hypothesis to be true conditioned on the observed p-values and therefore, we don't need to perform a global correction for the tests. We have completely rewritten the statistical methods of the manuscript to reflect this fact.

10. How are sensitivity and resistance defined?

In our context of essentiality, we defined a cell line to be sensitive to a knock-down gene if its DEMETER score is below the selected threshold (the default threshold is -2), and to be resistant when it is above it. See the answer to question 5 for more details.

11. How many cases did the authors see where genes were more significant than transcripts? In other words, is this method biased toward claiming that significance will occur with transcripts rather than genes?

It depends on the tumor type. As an overall percentage, 52% of essential genes -considering all tumor types as a whole- are better pinpointed by genes rather than by transcripts. Since the number of transcripts is much larger than the number of genes, it does not seem to be biased to select transcripts. Some tumor types show a larger proportion of transcript biomarkers and these results are in concordance with the bibliography -as shown in the results section.

12. The Discussion section presents results that had not been discussed before that point. A Discussion section should provide perspective on material from Results but not introduce new results.

Thanks for the comment. We fixed it as shown in the following answers.

13. The authors state that, "We found that essential genes were expressed in more than 80% of cases—much more than expected by chance." Where is this evidence? What is expected by chance?

We have provided the supporting data of this evidence. We also moved this paragraph to the Results section:

“Although DEMETER’s authors performed some validations of their essentiality score, we did two simple tests to confirm its reliability. First, we checked that genes are expressed when they are essentials (DEMETER score < -2). We found that genes are expressed more than 1 TPM in 85% of the cases when they are essential versus 70% when they are non-essential. This difference is strongly significant (Wilcoxon test P-value < 2.2 e-16).”

14. The Discussion mentions findings for KRAS, BRAS (should that be BRAF?), NRAS, and PIK2CA, but there is no prior mention of this.

The paragraph where we discussed KRAS, BRAF (thanks for pointing it out), NRAS and PIK2CA tried to provide a critical view of DEMETER. They are not strictly results of our work: we used them to double-check DEMETER results. After performing these analyses, we felt quite confident about the DEMETER scores and we kept on working with these data.
We have moved this paragraph to the result section.

15. How did the authors decide what is an activating mutation or not?

We did a manual case-by-case revision of the bibliography and identified that, for example, KRAS mutations are activating.

16. The Discussion mentions confirming relationships between TP53 and MDM2 and that these relationships are in accordance with the bibliography. This statement is vague.

Thanks for the comment. We agree with the reviewer. We have detailed the statement and cited the supporting reference:

“We confirmed that the mutation status of TP53 affects the essentiality of MDM2 and MDM4 as expected, since MDM4 regulates the activity of TP53 and MDM2 regulates its stability of TP53 [19]. “

[19] Toledo F, Wahl GM. MDM2 and MDM4: p53 regulators as targets in anticancer therapy. Int J Biochem Cell Biol. 2007;39:1476–82.

17. The Discussion mentions an analysis of mutations in EIF4E -> IRAK1 essentiality, but no mention is made in Results.

Using the data in CCLE, we coarsely evaluated the ability of mutations to predict IRAK1 essentiality. For this particular gene, the best mutations were outperformed by the proposed isoform as a biomarker. This case can be considered anecdotal so we decided to remove it from the paper: a thorough analysis would require deriving an equivalent algorithm to TranscriptAchilles for mutations. The derivation of this statistical framework is not trivial since the summarization of the mutations that appear in a gene requires a deep analysis of their types, aminoacid residues affected, presence of hypermutated cell lines, etc.

Minor:
18 In the app, it appears there are two places to change the DEMETER cutoff. This seems redundant.

Fixed. Thanks for pointing it out.

19 In the manuscript, I strongly recommend that you spell out "cell lines" rather than use an acronym. You're not saving many letters, and it can be confusing to readers.

Fixed. Thanks for pointing it out.

20. Because some people are color blind, use a color-blind safe color palette in the bar graphs and to distinguish the isoforms. Colorbrewer2.org has useful advice on this.

Thanks for the advice. We have used the Colorbrewer's colorblind safe palette to re-plot Figures both in the manuscript and in the application.

21. "TranscriptAchilles has been developed using the Docker container framework." This wording should be more precise. Docker is not a development tool.

Thanks for pointing this out. The cited wording has been modified to:
"The application can also be run locally within Docker to avoid installation problems and to facilitate reproducibility."

22. Figure 2: Use colorblind-friendly color scheme.

Fixed. Thanks for pointing it out.

23. Figure 4b: Has no x-axis label.

Fixed. Thank you for the comment. The x-axis label is shared between panels B and C. We have added this indication to the legend: "Samples in panels B and C are sorted in the same order. The x-axes are shared by both panels."

24. "We used Kallisto to quantify annotated isoforms." This wording should be modified because you didn't do the actual quantification.

The reviewer is right. The sentence "We used Kallisto to quantify annotated isoforms." has been replaced with the following: "The quantification of isoforms was done in a previous work using Kallisto".

The reference of this work was already cited in the Methods section: "The transcriptome profiles of these samples were calculated in a previous study [41] from raw RNA sequencing data [...] ".

[41] Tatlow PJ, Piccolo SR. A cloud-based workflow to quantify transcript-expression levels in public cancer compendia. Sci Rep

25. Many people will not know what AWS is.

The entire name “Amazon Web Services” has been used instead of the acronym AWS. Thanks for pointing this out.

Reviewer #2: In this manuscript, the authors describe a bioinformatics tool, TranscriptAchilles, for identifying gene targets and transcript biomarkers in cancer. To do that, they have developed a pipeline to integrate the RNAi screening data from Project Achilles and CCLE RNASeq data, a statistical model for identifying gene and transcript biomarkers, and a website for accessing and analyzing the data.

Overall, the question is interesting and the implementation in terms of pipeline building and software development was also solid. Making the software open source is also great for sharing with the community. The statistical methods should be rigorously reviewed.

Although the authors intended to build a tool for users without bioinformatics background, the tool still requires a lot of background in statistics in order to understand the results. For example, it would be informative to list the three criteria for select essential genes in the results page. For users who are not familiar with the scores used (e.g. DEMETER score), it would be helpful to provide some explanation.

We have completely reviewed the statistical methods and included a new figure to state more clearly the approach that we followed.

We have also included a user’s guide throughout the application. This guide provides more info of the different concepts (e.g., DEMETER score, steps to perform the different analyses, criteria for selecting essential genes, the meaning of the sliders and a basic explanations of the statistical methodology). We also refer the user to the reference publication, where he or she will be able to find more information about the statistical analysis.

In addition, since the application may be too complex to someone with no bioinformatics or statistical background, we have “relaxed” our claim of not needing bioinformatics background with the following sentence: “TranscriptAchilles provides a user-friendly web interface to identify transcript or gene biomarkers of gene essentiality.”

Close
