# Supplementary material for: TranscriptAchilles: a genome-wide platform to predict isoform biomarkers of gene essentiality in cancer
Source: Gigascience. 2019 Apr 3;8(4):giz021. doi: 10.1093/gigascience/giz021 (PMC6446222; doi:10.1093/gigascience/giz021)
Supplement: Response-to-Reviewer-Comments_Revision-1.pdf [file giz021_response-to-reviewer-comments_revision-1.pdf]

Author's Response To Reviewer Comments

Close

Thank you for your comments. We have included a point-by-point response to the reviewers.

“Reviewer reports:
Reviewer #1: The authors addressed my comments. I have a few additional comments.
1. Maybe I am missing it, but I could not find where I can download the data files used in the app. It is important to make these easily and openly accessible for reproducibility and for data sharing reasons.”

Thank you for pointing it out. We have added a “download data” section in the user’s guide of GitLab (https://gitlab.com/fcarazo.m/transcriptachilles/). In this section, we have included the paths where data can be downloaded including both the integrated data and the original data [1].

[1] Tatlow PJ, Piccolo SR. A cloud-based workflow to quantify transcript-expression levels in public cancer compendia. Sci Rep

“2. I was able to build the Docker container successfully. But I wasn't sure what command to run after that to start the web server. Please clarify this.”

Thanks. We have included the command to start the web server in the user’s guide of GitLab (https://gitlab.com/fcarazo.m/transcriptachilles/) and referred the reader to the “Getting Started” page of ShinyProxy to get more information about the configuration. In order to test the Docker image, the most straightforward method is to download the docker from Docker hub ("Build Docker from Docker Hub") and run the container ("Run TranscriptAchilles in a Docker App").

“3. You might consider pushing your Docker image to Docker Hub so that others don't need to build it before running it.”

Thank for the comment. We have pushed the docker image to Docker Hub and included the instructions to build it in the documentation of GitLab (https://gitlab.com/fcarazo.m/transcriptachilles/). We tested that it works in Windows, Linux Ubuntu and Mac OSX.

“Nice paper and tool!”

We really appreciated that!

Close
