# Supplementary material for: TranscriptAchilles: a genome-wide platform to predict isoform biomarkers of gene essentiality in cancer
Source: Gigascience. 2019 Apr 3;8(4):giz021. doi: 10.1093/gigascience/giz021 (PMC6446222; doi:10.1093/gigascience/giz021)
Supplement: Reviewer-1-Report-Original-Submission -- Stephen R Piccolo, Ph.D. — 11/7/2018 Reviewed [file giz021_reviewer-1-report-original-submission.pdf]

Reviewer Report

Title: TranscriptAchilles: a genome-wide platform to predict isoform biomarkers of gene essentiality in cancer

Version: Original Submission **Date:** 11/7/2018

Reviewer name: Stephen R Piccolo, Ph.D.

Reviewer Comments to Author:

The authors describe a new software tool for examining relationships between gene essentiality and transcript/gene expression in cell lines. This tool has the potential to help researchers without computational skills to sift through the Achilles data and make novel correlations, potentially to aid in identifying candidate drug biomarkers. The authors have placed an emphasis on ensuring the software is easy to install and use. The software is freely available. Below are some additional comments and suggestions for improving the manuscript. I have divided these into Major and Minor points.
Major
* Installation: dependencies change over time, so the exact install code may need to change. It would be better to put this install code on the GitHub site (already there) rather than in the paper.
* The paper has no instructions for executing the app locally within Docker (which can help to avoid R package installation issues).
* How long will the AWS site be up? Perhaps better is to provide more detailed instructions for running it locally within Docker (see comments above).
* I am not convinced that there is statistical evidence that the differences among cancer types (Figure 2) are significant. Also, if such high proportions of genes are potential biomarkers of essentiality, are we narrowing things down enough (maybe it is sensitive but not specific)? Please clarify.
* The manuscript (especially toward the beginning is not clear enough on how it defines predictive biomarkers. What exactly is being predicted? It appears it is drug sensitivity, but it also mentions predicting essentiality. Is there an assumption that these are necessarily linked? If so, on what basis. Maybe I am just slow to understand, but please clarify.
* In the case study, the authors used a minimum of 1 TPM. This is an extremely low threshold (likely well within error bounds of no expression). Do the authors have evidence to support that this threshold should be used?
* In the case study, the authors found 73/121 known cancer genes, including two that are already known to play a role in renal carcinoma. That is a long list, and you are bound to find known cancer genes any time you study cell lines. Is there any experimental evidence that these genes actually drive cancer resistance or sensitivity in renal carcinoma?
* In describing the case study, the authors state that, "expression is not capable of distinguishing between sensitive and resistant groups of CLs." But no data is provided to support this claim.
* I am not sure I understand at what point the ldfr values are calculated. Is it for each combination of "essential gene" and potential biomarker? If so, you are bound to have lots of false positives because there is such a high number of combinations for which you could perform this test. You would need

some kind of global correction for multiple tests.
* How are sensitivity and resistance defined?
* How many cases did the authors see where genes were more significant than transcripts? In other words, is this method biased toward claiming that significance will occur with transcripts rather than genes?
* The Discussion section presents results that had not been discussed before that point. A Discussion section should provide perspective on material from Results but not introduce new results.
* The authors state that, "We found that essential genes were expressed in more than 80% of casesâ€”much more than expected by chance." Where is this evidence? What is expected by chance?
* The Discussion mentions findings for KRAS, BRAS (should that be BRAF?), NRAS, and PIK2CA, but there is no prior mention of this.
* How did the authors decide what is an activating mutation or not?
* The Discussion mentions confirming relationships between TP53 and MDM2 and that these relationships are in accordance with the bibliography. This statement is vague.
* The Discussion mentions an analysis of mutations in EIF4E -> IRAK1 essentiality, but no mention is made in Results.
Minor:
* In the app, it appears there are two places to change the DEMETER cutoff. This seems redundant.
* In the manuscript, I strongly recommend that you spell out "cell lines" rather than use an acronym. You're not saving many letters, and it can be confusing to readers.
* Because some people are color blind, use a color-blind safe color palette in the bar graphs and to distinguish the isoforms. Colorbrewer2.org has useful advice on this.
* "TranscriptAchilles has been developed using the Docker container framework." This wording should be more precise. Docker is not a development tool.
* Figure 2: Use colorblind-friendly color scheme.
* Figure 4b: Has no x-axis label.
* "We used Kallisto to quantify annotated isoforms." This wording should be modified because you didn't do the actual quantification.
* Many people will not know what AWS is.

Level of Interest

Please indicate how interesting you found the manuscript: Choose an item.

Quality of Written English

Please indicate the quality of language in the manuscript: Choose an item.

Declaration of Competing Interests

Please complete a declaration of competing interests, considering the following questions:

- Have you in the past five years received reimbursements, fees, funding, or salary from an organisation that may in any way gain or lose financially from the publication of this manuscript, either now or in the future?
- Do you hold any stocks or shares in an organisation that may in any way gain or lose financially from the publication of this manuscript, either now or in the future?
- Do you hold or are you currently applying for any patents relating to the content of the manuscript?
- Have you received reimbursements, fees, funding, or salary from an organization that holds or has applied for patents relating to the content of the manuscript?
- Do you have any other financial competing interests?
- Do you have any non-financial competing interests in relation to this paper?

If you can answer no to all of the above, write 'I declare that I have no competing interests' below. If your reply is yes to any, please give details below.

I declare that I have no competing interests

I agree to the open peer review policy of the journal. I understand that my name will be included on my report to the authors and, if the manuscript is accepted for publication, my named report including any attachments I upload will be posted on the website along with the authors' responses. I agree for my report to be made available under an Open Access Creative Commons CC-BY license (http://creativecommons.org/licenses/by/4.0/). I understand that any comments which I do not wish to be included in my named report can be included as confidential comments to the editors, which will not be published.

I agree to the open peer review policy of the journal

To further support our reviewers, we have joined with Publons, where you can gain additional credit to further highlight your hard work (see: https://publons.com/journal/530/gigascience). On publication of this paper, your review will be automatically added to Publons, you can then choose whether or not to claim your Publons credit. I understand this statement.

Yes Choose an item.
